# The Effect of Heat Sterilization on Key Filtration Performance Parameters of a Commercial Polymeric (PVDF) Hollow-Fiber Ultrafiltration Membrane

**DOI:** 10.3390/membranes12080725

**Published:** 2022-07-22

**Authors:** Alexandra Nastouli, Asimina Tsirigka, Michael Harasek, Anastasios J. Karabelas, Sotiris I. Patsios

**Affiliations:** 1 Laboratory of Natural Resources and Renewable Energies, Chemical Process & Energy Resources Institute (CPERI), Centre for Research and Technology-Hellas (CERTH), GR 57001 Thessaloniki, Greece; a.nastouli@certh.gr (A.N.); tsirigka@certh.gr (A.T.); karabaj@certh.gr (A.J.K.); 2 Institute of Chemical, Environmental and Bioscience Engineering, TU Wien, AU 1040 Vienna, Austria; michael.harasek@tuwien.ac.at

**Keywords:** ultrafiltration, polyvinylidene difluoride (PVDF), molecular weight cut-off (MWCO), membrane permeance, steam sterilization, fermentation

## Abstract

Membrane processes can be integrated with fermentation for the selective separation of the products from the fermentation broth. Sterilization with saturated steam under pressure is the most widely used method; however, data concerning heat sterilization applicability to polymeric ultrafiltration (UF) membranes are scarcely available. In this study, the effect of the sterilization process on the filtration performance of a commercial polyvinylidene difluoride (PVDF) hollow fiber UF membrane was evaluated. Membrane modules were constructed and sterilized several times in an autoclave. Pure water flux tests were performed, to assess the effect of heat sterilization on the membrane’s pure water permeance. Dextran rejection tests were performed for the characterization of membrane typical pore size and its fouling propensity. Filtration performance was also assessed by conducting filtration tests with real fermentation broth. After repeated sterilization cycles, pure water permeance remained quite constant, varying between approx. 830 and 990 L·m^−2^·h^−1^·bar^−1^, while the molecular weight cut-off (MWCO) was estimated to be in the range of 31.5–98.0 kDa. Regarding fouling behavior, the trans-membrane pressure increase rate was stable and quite low (between 0.5 and 7.0 mbar/min). The results suggest that commercial PVDF UF membranes are a viable alternative to high-cost ceramic UF membranes for fermentation processes that require heat sterilization.

## 1. Introduction

Biotechnological processes routinely require sterile environments, which means that all equipment, materials, and substrates used should be free of any microbial contamination. The process of destroying, inactivating, or permanently removing all microorganisms by physicochemical methods, is defined as sterilization [1]. Sterilization processes are divided into four main categories, i.e., heat, radiation, chemical, and sterile filtration. The selection of the applied sterilization method may differ based on many parameters, such as the material to be sterilized (e.g., solid/liquid/gas), the industrial field (e.g., pharmaceutical, biotechnology, etc.), the final product, the cost, and the equipment used [2]. In biotechnological processes, heat sterilization is the most commonly applied method to achieve sterile conditions with relatively low cost [3]. Under high temperature, saturated steam leads to denaturation of components with high importance for the microbial life, such as proteins; exposure to these conditions for sufficiently long periods results in microbial death [1]. However, boiling temperature of water, i.e., 100 °C, is not high enough to inactivate endospores. Thus, higher temperatures are achieved under pressure (e.g., in autoclaves), while the duration of the sterilization process may be shortened; i.e., a typical sterilization process is carried out at 120 °C for 20 min. In the case of liquid culture media in a bioreactor, in-place steam sterilization or a continuous sterilizer are usually employed at industrial scale [2,4], whereas for lab-scale fermentation experiments sterilization in autoclaves is the most common method [5,6,7,8].

Fermentation is a well-known process for the production of a variety of biotechnological products, through the biological conversion of the substrate to different products by specific fungi, yeast, and bacteria species. Sterile conditions in the bioreactor are necessary for the success of the fermentation process [8,9,10,11]. Fermentation may be combined with a membrane process for the selective separation of the products from the fermentation broth, or for recycling the microorganism cells [12]. Depending on the scope of the membrane separation process and the characteristics of the final product, different types of membranes can be chosen, such as microfiltration (MF), ultrafiltration (UF), or nanofiltration (NF) [11,12,13]. The membrane separation process can be either integrated with the fermentation, or it can be employed as a separate process step [10,11,12,13]. In the former case, the integrated bioreactor-membrane separation process is defined as a membrane bioreactor (MBR) [14], which is an advancement of conventional fermentation technology for the production of energy, biofuels, and biomaterials.

A variety of membrane materials have been used in MBR processes. Depending on the materials used, their chemical, mechanical, and physical properties, such as thermal stability, chemical resistance, molecular heterogeneity/homogeneity, etc., differ considerably [15]. UF membranes, most commonly used in biotechnological applications [16], are usually made of ceramic or polymeric materials such as polysulfone (PS), polyethersulfone (PES), polyvinylidene difluoride (PVDF), aromatic polyamide (PA), cellulose acetate (CA), etc. [11,15,17]. Whilst ceramic membranes are more robust in terms of resistance to fouling, chemical compatibility, and thermal stability (i.e., during the sterilization process), their use remains limited to niche applications, primarily due to their relatively high cost compared to polymeric membranes [18].

In MBR systems used for fermentation processes, the membranes also have to be sterilized, especially in the case of a continuous process or when the membrane module is submerged in the bioreactor. A variety of sterilization methods, depending on the type of membrane used, have been described in the literature, while autoclave sterilization remains the most common method for the fermentation process [8,10,13,19,20]. Most of the reported studies for MBRs in fermentation processes employ chemical sterilization, using mainly a NaClO solution; however, there are a handful of studies that employ autoclave sterilization for both polymeric and ceramic membranes. For instance, Mimitsuka et al. [21] set up a membrane-integrated fermentation reactor, where a PVDF flat sheet membrane was used, autoclaved in situ through heat sterilization (121 °C, 20 min). The authors report that the pure water permeance of the membrane was unaffected before and after autoclaving; however, no relevant data are presented. It is also unclear whether, or how many times, the PVDF flat sheet membrane was reused after repeated sterilization cycles. Krige and Nicol [13] employed a side-stream hollow fiber membrane to recycle *E. coli* cells and to increase the productivity of succinic acid fermentation. The membrane, made of PS, was thermally sterilized (121 °C, 40 min); however, there are no reported data on the effect of the sterilization process to the membrane filtration performance. The authors also failed to mention any results regarding the filtration efficiency or the fouling propensity of the membrane, which led to higher volumetric productivity of the integrated system compared to batch fermentation. Ramchandran et al. [22] applied the recommended pretreatment protocol (1% sodium hypochlorite for 18 h) of the membrane manufacturer to the membrane modules used in a fermentation process. Then, they rinsed the membranes with Milli-Q water, and finally immersed them in sterile water. The membrane modules were single-use, which may explain the adoption of a rather mild sterilization protocol.

Table 1 summarizes the membrane type used and the sterilization methods applied in MBR systems; i.e., bioreactor fermentation coupled with membrane separation. In most cases, experiments took place in bench-scale fermenters of 1.5–5 L volume [6,13,21,23,24], while a few researchers focused on studying the fermentation process at higher volumes, with fermenters up to 15 L [11,20]. Membrane modules were re-used in the majority of the studies, although it is not clear how many times the membranes could be reused. In other cases (e.g., [22]), single-use membrane modules were employed in each experiment, which is apparently unsustainable from an economical point of view. Concerning the effect of the sterilization process on the membrane separation efficiency and the filtration performance (i.e., permeance, fouling rate, etc.), there are hardly any data on the aforementioned studies.

Sawai et al. [27] developed an MBR process for pyruvic acid production in continuous culture. The membrane employed (flat-sheet UF, PVDF, 0.08 μm) was thermally sterilized and its filtration performance was excellent, when operated at sub-critical flux values, i.e., below 17 L·m^−2^·h^−1^ (LMH). The trans-membrane pressure (TMP) gradually increased during the first 20–30 h of fermentation, and it stabilized to a value of approx. 4–5 kPa, throughout the continuous fermentation that lasted 400 h. These results indicate that the PVDF membrane filtration performance is stable despite the heat sterilization process. However, there are no data concerning the performance of the non-sterilized membrane, nor any data for the membrane filtration performance after repeated sterilization cycles. The same research group [26] reported the production of D-lactic acid continuously obtained in an MBR through the cultivation of three different bacteria strains—*S. inulinus*, *S. laevolacticus*, and *S. terrae*. The same UF membrane (i.e., flat-sheet, PVDF, 0.08 μm) was used to separate the cultivated broth from the bacteria cells at subcritical flux operation which varied from 7.2 to 14.4 LMH. The thermally sterilized membrane exhibited excellent filtration performance since the TMP remained below 5 kPa during the whole fermentation process that lasted approx. 300 h. Again, no data were reported for the effect of repeating sterilization cycles on the membrane filtration performance.

PVDF polymeric membranes are also widely used for wastewater treatment and for water filtration; both flat-sheet and hollow-fiber configurations are commercially available. PVDF hollow-fiber membranes are commercially available since the early 2000s [32,33] and it is estimated that such membranes comprise more than half (i.e., 55%) of the total membrane area offered for submerged MBRs, covering most of the pore-size ranges of interest (i.e., between 0.03 and 0.4 μm) for wastewater treatment applications [34]. PVDF membranes provide high performance properties such as high mechanical strength, thermal stability, chemical resistance [35], a rather short and controllable pore-size range, and reasonable pricing compared to high-cost ceramic UF membranes. PVDF hollow-fiber membranes are generally comprised of a thin surface layer (active layer), which provides the required selectivity, on top of a thicker, porous support, usually made from polyester or other non-woven polymeric fabrics, which affords mechanical stability.

Concerning thermal stability of hollow-fiber polymeric membranes for MBRs, PVDF is a highly non-reactive thermoplastic fluoropolymer, which has been widely investigated due to its thermal degradation resistance. Thermogravimetric analysis and pyrolysis gas chromatography are some of the methods used for the thermal degradation study and assessment. The high thermal stability is explained by the high electronegativity that fluorine atoms provide, as well as by the strong C-F bond. According to the literature, degradation of PVDF, which is used in the form of powder for membrane fabrication [36], can differ depending on its spherulitic forms [37]. Table 2 summarizes the thermal characteristics of PVDF according to [37]. Polyester, used as support layer for the construction of commercial hollow fiber membranes, is a well-known thermosetting polymer. Bastiurea et al. and Low and Bakar [38,39] investigated the thermal behavior of polyester composites. According to Low and Bakar [39], glass transition temperature (Tg) of polyester composites is 64.9 °C and thermal decomposition starts at temperature higher than 219 °C. Bastiurea et al. [38] shows that mass loss of pure polyester increases at quite high temperatures (i.e., T = 320 °C), while the glass transition temperature (Tg) of pure polyester composites is 55.9 °C. The aforementioned data indicate that PVDF and polyester polymers are expected to exhibit increased thermal resistance properties at commonly applied thermally sterilization conditions.

Non-supported flat-sheet PVDF membranes, commercially available for sterile filtration applications, are reported to withstand either in-line steam sterilization (135 °C, 30 min) for 150 cycles or autoclaving (130 °C, 30 min) for 400 cycles [40]. The preceding short review (Table 1) also indicates that non-supported flat-sheet PVDF membranes withstand common heat sterilization procedures employed during fermentation applications. However, there are no studies to assess the effect of heat sterilization on the performance of supported hollow-fiber PVDF membranes, which are commercially available. Moreover, reported data from previous studies do not clearly specify whether the membranes can be reused through repeated heat sterilization for multiple fermentation cycles.

Therefore, the objective of this study is to evaluate the effect of a standard heat sterilization protocol (e.g., at 121 °C temperature for 20 min), on the membrane characteristics and the filtration performance of a commercial supported PVDF/polyester hollow-fiber UF membrane (PURON MBR, Koch Separation Solutions, MA, USA). Different methods can be employed to characterize the membrane’s properties and assess its filtration performance under specific conditions. Morphology, pore size distribution, permeance, and selectivity are some of the membrane characteristics that define important membrane properties and can potentially be affected during a heat sterilization process [16,41]. Molecular weight cut-off (MWCO) is another important parameter that characterizes porous membranes [16,42], that is commonly estimated through the performance of dextran rejection tests [16,43,44].

In this study, the pure water permeance, the typical MWCO, the rejection efficiency, and the fouling behavior of the commercial PVDF/polyester hollow-fiber UF membrane were the main parameters selected to assess the membrane characteristics and filtration performance after repeated heat sterilization cycles (10 cycles in total) with saturated steam under pressure. Pure water flux tests were performed to assess the effect of heat sterilization on the permeance of the membrane. To characterize the typical membrane pore size as well as its filtration performance, dextran rejection tests were performed. Concerning the fouling rate during the dextran rejection tests, the TMP temporal profile was monitored and its increase rate (i.e., ΔTMP/Δt) was calculated as a representative fouling index. Finally, short filtration tests with real fermentation broth of a yeast (i.e., *Yarrowia lipolytica*) cultivation were performed to assess the membrane filtration performance (after repeated heat sterilization cycles) under relevant operational environment.

## 2. Materials and Methods

### 2.1. Experimental Set-Up/Analytical Methods

#### 2.1.1. Membranes

Commercial PVDF hollow fiber UF membranes (PURONR MBR, Koch Separation Solutions, Massachusetts, USA) were assessed in this work. The handmade membrane modules were designed to be suitable for the head-plate and the height of a 3 L lab-scale bioreactor (BioFlo 120, Eppendorf, Hamburg, Germany). The main characteristics of the membranes used are summarized in Table 3. Stainless steel fittings and 2-component epoxy adhesive were used for the construction of the module. The handmade membrane module consisted of 16 hollow fibers of 18 mm length, and it had a total filtration area of 235 cm^2^. Three similar modules were constructed.

#### 2.1.2. Filtration Set-Up

**Figure 1:**
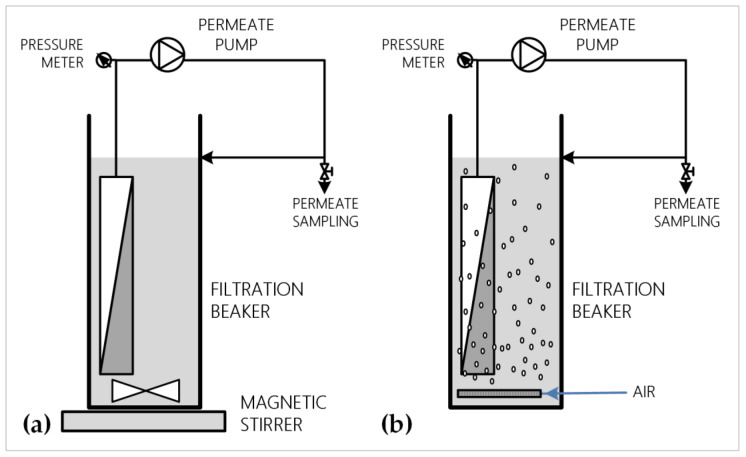
Experimental set-ups used in this study: (**a**) filtration set-up for pure water and dextran solutions filtration tests; (**b**) filtration set-up for fermentation broth filtration tests.

Filtration tests were performed at the experimental set-ups are presented in Figure 1. The filtration tests were performed in beakers of 1 L volume. The membrane module was submerged in the filtration solution, which was continuously stirred with a magnetic stirrer during the pure water and dextran solutions filtration tests (Figure 1a). For the filtration tests of the fermentation broth, air (0.23 vvm) was employed to agitate the fermentation broth and to maintain favorable aerobic conditions for the yeast cells (Figure 1b). A piston pump (Fluid Metering Inc., New York, USA), controlled through a programmable logic controller (PLC) system (LG Glofa LS Programmable Logic Controller G7M-DR40U, LS Electric, Anyang, South Korea), was used to maintain constant flux operation. Permeate flow was periodically paused to control membrane fouling. A pressure transducer (Tecsis GmbH, Offenbach, Germany), connected at the outlet of the membrane module, enabled monitoring of the TMP as an indicator of membrane fouling.

#### 2.1.3. Strain and Media

*Yarrowia lipolytica MUCL 28849*, an oleaginous yeast, was used for this study. The strain stocks were stored in 25% *v/v* glycerol at −80 °C. The strain was grown in a glass tube of total volume 5 mL yeast extract–peptone–glycerol (YPG) with 2% *v/v* glycerol, for 6 h at 30 °C. The preculture media was composed of: 53 g glycerol, 3.0 g (NH_4_)_2_SO_4_, 2.0 g KH_2_PO_4_, 2.6 g Na_2_HPO_4_ × 2 H_2_O, 1.0 g MgSO_4_ × 7 H_2_O, 0.2 g CaCl_2_ × 2 H_2_O, 0.5 g citric acid, 20 mg FeCl_3_, 1 mg thiamin-HCl, 0.5 mg H_3_BO_3_, 0.06 mg CuSO_4_ × 5 H_2_O, 0.1 mg KI, 0.45 mg MnSO_4_ × H_2_O, 0.71 mg ZnSO_4_ × 7 H_2_O, and 0.23 mg Na_2_MoO_4_ × 2 H_2_O, per liter of deionized water. The same media was used for the fermentation process.

#### 2.1.4. Dextrans

A buffered solution (pH = 7.0) of a mixture of polydisperse dextran fractions in 50 mM potassium phosphate monobasic (KH_2_PO_4_) was used as the dextran filtration solution. A mixture of dextran polymers (150 kDa and 500 kDa) within the molecular weight (MW) size range of the UF membrane was used. Dextran was purchased from Alfa Aesar (Haverhill, MA, USA). The relative concentration of the different MW fractions of the dextran mixture was analyzed using a gel permeation chromatography (GPC) method.

#### 2.1.5. High Performance Liquid Chromatography (HPLC)

The GPC method was set up in an HPLC, Shimadzu LC-10AT VP Liquid Chromatograph (Shimadzu Europa GmbH, Duisburg, Germany), using a GPC column (PL aquagel-OH Mixed 8 μm, 300 × 7.5 mm, Agilent Technologies Inc., Santa Clara, CA, USA) and a Shimadzu Refractive Index Detector RID-10A (Shimadzu Europa GmbH, Duisburg, Germany). PL polysaccharide standards (Polymer Laboratories Ltd., Church Stretton, UK) of different narrow-range MWs (between 22.8 and 788.0 kDa) were used for the calibration curve. As mobile phase, 50 mM potassium phosphate monobasic (KH_2_PO_4_) was used, and the flow rate was set to 1.0 mL/min. In the same HPLC system, samples from the fermentation broth (after centrifugation at 4500 rpm for 6 min, and filtration through 0.45 μm filters) and from its UF permeate were analyzed by a Shodex Sugar SH1011 (8.0 mm I.D. × 300 mm) column and detected by a Shimadzu SPD-M20A UV detector (Shimadzu Europa GmbH, Duisburg, Germany), to analyze the concentration of organic acids that are major metabolites of the *Y. lipolytica* yeast cultivation. Furthermore, 14 mM H_2_SO_4_ was used as the eluent. The flow rate was set to 0.4 mL/min, and the oven temperature to 40 °C.

#### 2.1.6. Total Organic Carbon (TOC)

Total organic carbon was measured using a Shimadzu TOC-5000A (Shimadzu Europa GmbH, Duisburg, Germany), total organic carbon (TOC) analyzer during the cleaning protocol of each new membrane module, to verify the efficiency of the employed protocol. A calibration curve of 0.1–1.0 ppm was used for the feed and the permeate pure water samples.

#### 2.1.7. Optical Density (OD_600_) and Cell Dry Weight (CDW)

Optical density was measured for cell growth determination at 600 nm by a photometer (UV1700 Pharmaspec Shimadzu UV-VIS Spectrophotometer, Shimadzu Europa GmbH, Duisburg, Germany). Furthermore, cell dry weight (CDW) was determined by drying of double washed cell pellets obtained from 1 mL culture broth. Samples were centrifuged at 4500 rpm for 6 min by a centrifuge (Thermo Scientific Heraeus Megafuge 16R, Thermo Fisher Scientific Inc., Waltham, MA, USA), and dried at 60 °C until reaching constant weight.

#### 2.1.8. Scanning Electron Microscope (SEM) Images

Small sections of the membrane hollow fibers (approx. 2 cm long) were cut from the pristine membrane (Sample 1), after the 10^th^ sterilization cycle (Sample 2), and after the respective filtration test of the fermentation broth (Sample 3). The membrane samples were left to dry overnight under ambient conditions and were gold sputtered to be analyzed using an SEM (JSM-IT500, JEOL Ltd., Tokyo, Japan).

### 2.2. Experimental Procedures

To study the filtration characteristics of the membrane after the heat sterilization (T = 121 °C, t = 20 min), three main parameters were chosen to be assessed; i.e., the membrane permeance, the membrane typical pore size, and the filtration performance (regarding fouling and rejection characteristics). Before each test, a specific cleaning protocol was employed. The membranes were first rinsed with clean DI water for at least 4 h to remove chemical preservatives, followed by cleaning with 200 ppm NaOCl solution for 1.0 h. A second rinsing with DI water was applied to remove any traces of the NaOCl cleaning solution. The total organic carbon (TOC) concentration of both the feed and permeate was periodically analyzed to verify that the difference between the TOC concentration of the feed and permeate did not exceed 5%.

#### 2.2.1. Membrane Permeance Assessment

Pure water flux tests were performed to assess the effect of the sterilization process on the pure water membrane permeance. Ten cycles of sterilization took place in an autoclave (Raypa Steam Sterilizer, Raypa, Barcelona, Spain) at 121 °C for 20 min, and pure water flux tests were performed before the first, and after each sterilization cycle. Filtration tests took place in beakers of volume 1 L. During filtration, the permeate was disposed of and new DI water was added to keep the filtration volume constant. The TMP was measured automatically every 15 s during each test. The temperature was not regulated but it was recorded during the test, and it fluctuated between 19 and 26 °C, mainly affected by the ambient conditions and the pump operating parameters. All measurements were corrected to a reference temperature of 25 °C, according to the following equation:

L_25_ = L_T_ × η_Τ/_η_25_ (1)

where: L_25_ and L_T_ are the permeabilities at 25 °C and the real test temperature, respectively, and η_25_ and η_T_ are the dynamic viscosities of water at 25 °C and the real test temperature, respectively. The TMP was recorded in triplicate for fluxes (J) between 10 and 50 LMH, and the permeance (L_T_) was calculated for each flux value as follows:

L_T_ = J/TMP (2)

#### 2.2.2. Membrane Typical Pore Size

A 2nd module was used for the estimation of the typical membrane pore size. Ten cycles of sterilization were performed again, and dextran rejection tests were performed, before the first and after each subsequent sterilization cycle. A mixture of dextran polymers within the size range of interest for the particular membrane was used. Each rejection test lasted for 30 min in a continuously stirred beaker of 1 L volume. Samples from the feed and permeate were collected during each rejection test. The results were used for the estimation of the MWCO of the membrane by calculating the R90 rejection factor (90% rejection) for each sample. Moreover, the TMP was recorded and the TMP increase rate (during the dextran rejection tests) was calculated as an estimate of the membrane fouling propensity during the dextran solution filtration. The TMP increase rate (R_TMP_) was calculated (Figure S1) using the following equation for Δt = 1 min time steps, excluding the recordings from the first 1.5 min (shaded area in Figure S1a), when transient pump operation occurred.

R_TMP_ = ΔTMP/Δt (3)

After each filtration test, the typical cleaning protocol was employed to restore the initial clean membrane permeance.

#### 2.2.3. Fermentation/Membrane Filtration Tests

A 3rd module was used for the assessment of the membrane filtration performance through three membrane filtration tests with real yeast fermentation broth before the first, and after the 5th and the 10th sterilization cycles. Three similar batch cultivations were carried out in a 3 L bioreactor (BioFlo 120, Eppendorf, Hamburg, Germany) of working volume 1.75 L with sterile defined medium, using an inoculum size of 100 mL preculture, and fermented for 20 h. The bioreactor was sterilized by autoclaving at 121 °C for 20 min. The temperature was set at 30 °C and the pH was adjusted to 4 by automated addition of 5 N H_2_SO_4_ and 5 N NaOH. The aeration rate and agitation speed were, respectively, adjusted to 1.0 vvm and 800 rpm, while the level of DO was continuously monitored. The cultivation time was 24 h, reaching 19.8 ± 0.87 g/L average biomass concentration. The fermentation broth (800 mL) was collected after each batch fermentation and used for the membrane filtration tests (3 in total). Each test lasted for 40 min (4 cycles of 8 min filtration—2 min relaxation), in a beaker of 1 L volume, where aeriation was applied (0.23 vvm) to maintain favorable aerobic conditions for the yeast cells. The TMP was continuously monitored to assess the fouling propensity of the membrane. After each filtration test, the typical cleaning protocol was applied, as already described, to restore the initial clean permeance.

## 3. Results and Discussion

### 3.1. Membrane Permeance

**Figure 2:**
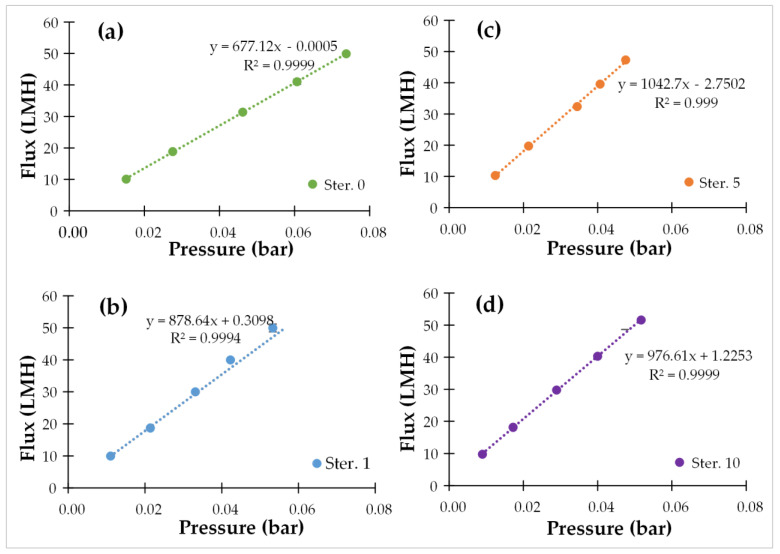
Pure water flux test results: (**a**) before sterilization (ster. 0); (**b**) after the 1st sterilization cycle; (**c**) after the 5th sterilization cycle, and (**d**) after the 10th sterilization cycle.

Pure water membrane permeance was assessed as a critical parameter concerning the effect of the heat sterilization process on the membrane characteristics. Typical results of the pure water flux tests are presented in Figure 2, before any sterilization, and after the 1st, 5th and 10th sterilization cycles. The flux increases linearly (R^2^ ≥ 0.99) with the applied pressure; therefore, the permeance is accurately determined through the slope of the respective lines.

The initial pure water permeance of the membrane module (i.e., before any sterilization cycle) was 677 LMH/bar, while after the 1st, 5th, and the 10th cycles of sterilization, pure water permeance was 878, 1043, and 977 LMH/bar, respectively. These values correspond to permeance before correction according to Equation (1). Data of temperature-corrected pure water permeabilities are reported in Table 4, where it is observed that the temperature-corrected pure water permeance increases to 879 LMH/bar after the first sterilization and fluctuates with a mean 906 ± 49 LMH/bar after the ensuing sterilization cycles. The SD of the 10 pure water permeances after each sterilization cycle is approx. 5.4% of the mean value, which indicates that the permeance is quite constant, varying between 829 (min. value) and 993 (max. value) LMH/bar. The average permeance after each sterilization cycle is approx. 33.8% higher than the initial permeance. This difference between the pristine and the sterilized membrane could be attributed either to the conditioning of the membrane after its first use or the slight increase of the membrane pore size. In general, the sterilization process does not seem to negatively affect the permeance of the membrane, since there are no high fluctuations, or a systematic trend of permeance increase or reduction. This conclusion is also supported by the fact that pure water permeance after sterilization is similar to the reference values of the commercial membrane [45].

### 3.2. Membrane Rejection

**Figure 3:**
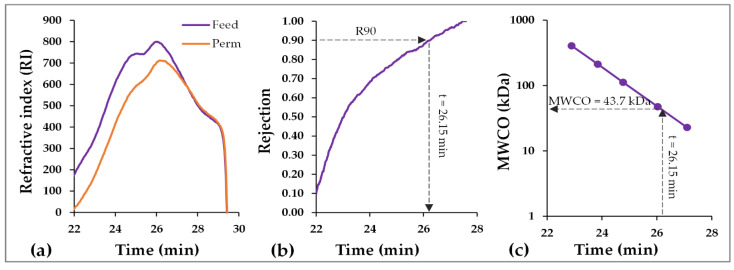
Typical data processing methodology for estimation of molecular weight cut-off (MWCO) from dextran rejection test results (data from the test after the 2nd sterilization cycle): (**a**) typical high performance liquid chromatography (HPLC) chromatogram from feed and permeate sample; (**b**) plot of the membrane rejection efficiency, determined by taking the ratio of RI time-series data of permeate and feed; (**c**) calibration curve with polysaccharide standards.

Membrane rejection performance was assessed by estimating its typical pore size. Dextran rejection tests were employed, involving samples from both the feed and the permeate which were analyzed using GPC-HPLC. Figure 3 depicts typical chromatograms of the feed and the permeate as well as the graph of the calculated R90 rejection factor. Each point corresponds to a specific MW according to the GPC calibration curve. When the rejection factor reaches 90%, rejection of the specific MW size (e.g., t = 26.15 min → 43.7 kDa) corresponds to the MWCO, thus providing an estimate of the typical pore size of the membrane. According to Table 4, MWCO fluctuates from 31.5 to 98.0 kDa, but again there is no clear trend related to the sterilization cycles. The initial MWCO is around 42.0 kDa, whereas the mean MWCO after each sterilization cycle is approx. 58.4 ± 22.1 kDa. The SD of the measurement is quite high (i.e., approx. 37.8% of the mean value); however, the typical experimental uncertainties and accuracy in MWCO measurements are quite high [16]. The typical pore size of the commercial UF membrane is 0.03 μm, which corresponds to approx. 60–80 kDa [46]. Therefore, both the initial and the estimated mean MWCO, after the sterilization cycles, are slightly lower than the nominal MWCO.

### 3.3. Membrane Fouling

**Figure 4:**
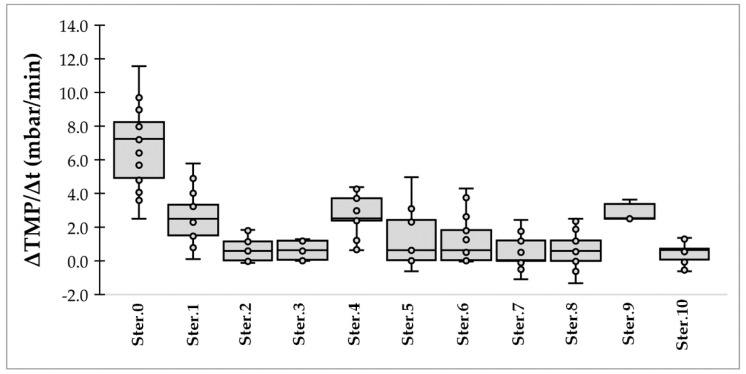
Variation of trans-membrane pressure (TMP) increase rate during the dextran rejection tests. The boxplot displays the minimum, lower quartile (Q25), the median, the upper quartile (Q75), and the maximum of the data set after each sterilization cycle; *n* > 16 for each filtration test.

The membrane filtration performance was assessed by estimating the fouling tendency during both the dextran rejection tests, and the filtration test with real fermentation broth. By collecting the data of TMP during dextran rejection tests, the distribution of the TMP increase rate is shown in Figure 4. The mean TMP increase rate (ΔTMP/Δt) for the thermally sterilized membrane varied between 0.5 and 3.0 mbar/min, whereas for the pristine membrane the mean TMP increase rate was 7.0 mbar/min. Therefore, the sterilization process does not seem to practically affect the membrane regarding its fouling behavior. In fact, the mean fouling rate for the pristine membrane (7.0 ± 2.2 mbar/min) is slightly higher compared to the membrane after being sterilized (Table 4); however, the fluctuation does not follow a trend that could correlate with the increasing number of sterilizations. The presented data (Figure 4) also correlate well with the slightly higher MWCO of the sterilized membrane compared to the pristine one.

**Figure 5:**
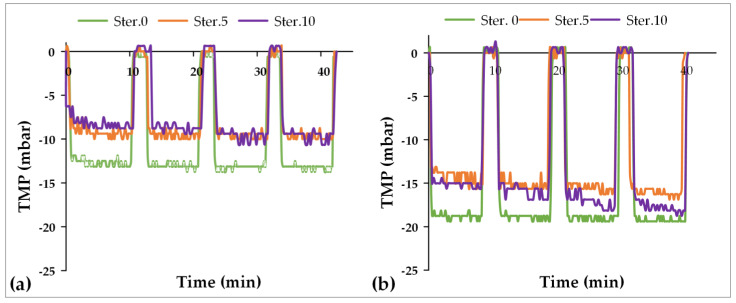
Temporal variation of TMP in the filtration tests performed using *Yarrowia lipolytica MUCL 28849* fermentation broth for the pristine membrane and the same membrane after the 5th and the 10th sterilization cycles: (**a**) filtration test at 10 LMH flux; (**b**) filtration test at 15 LMH flux.

The membrane fouling tendency was further assessed through filtration tests of real fermentation broth. Figure 5 depicts the results of the TMP temporal variation during the filtration tests for the pristine membrane as well as after the 5th and 10th cycles of sterilization at two different operating fluxes, i.e., 10 and 15 LMH. As expected, the TMP is higher when 15 LMH flux is applied compared to 10 LMH. Concerning 10 LMH flux (Figure 5a), the TMP, after the initial sharp pressure drop when the filtration test starts (pump on), varied between 12 and 14 mbar before the first sterilization of the membrane module, while after being sterilized 5 and 10 times, the TMP was practically constant at 9–10 mbar. The difference of the TMP values of the pristine membrane, compared to the sterilized one, agrees with the difference of the pure water permeance as well as the estimated MWCO. Similar behavior is observed during the filtration at 15 LMH (Figure 5b). Concerning the pristine membrane, the TMP reaches nearly 20 mbar and then remains practically constant, while after the 5th and 10th cycles of sterilization, the TMP is again slightly lower, reaching approx. 15–19 mbar. However, after the initial transitional pressure drop, a slight but continuous increase of the TMP is observed, which is more pronounced after the 10th sterilization cycle and at longer filtration time (i.e., after t = 20 min). From 0 to 10 min of filtration, TMP is rather stable around 15 mbar, while from the 30th to 40th min of filtration, the TMP increases by approx. 2.0 mbar. Although this increase is quite low, it is a trend that should be further studied in longer filtration tests. Overall, the membrane seems to be slightly affected by the sterilization process, especially when the number of sterilization cycles is increased, and when the applied fluxes are relatively higher.

**Figure 6:**
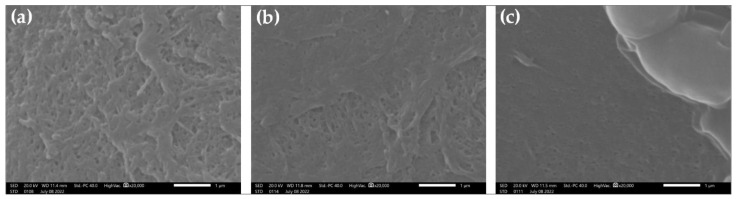
Scanning electron microscopy images of: (**a**) the pristine membrane; (**b**) the clean membrane after the 10th sterilization cycle; and (**c**) the same membrane after the fermentation filtration test.

SEM images from the pristine membrane, the clean membrane after the 10th sterilization cycle, and the same membrane after the fermentation broth filtration test are presented in Figure 6. The membrane pores and the structure of the membrane active layer are clearly visible in Figure 6a,b. Upon first inspection it seems that there is no visible alteration in the membrane (e.g., pore size) or surface characteristics between the pristine membrane and the membrane after 10 sterilization cycles. On the contrary, the membrane surface in Figure 6c seems to be lightly fouled, since its surface is partially covered by a slimy fouling layer. Some membrane pores are still visible, which agrees with the fouling rate that is rather low (Figure 5). Furthermore, some yeast cells (at the upper right corner of the image) seem to be attached to the membrane surface. Their size (typically 2–3 μm) is many times larger than the membrane’s pores, a fact that is also obvious by the excellent rejection rate of the yeast cells and the highly transparent filtration permeate. No apparent mechanical failure (e.g., cracks or pilling-off) is observed for the (multiple times) sterilized membrane, confirming the thermal resistance properties of the membrane’s active layer material (PVDF).

Samples from the feed and permeate were collected during fermentation broth filtration tests for (OD_600_) measurements. The mean OD_600_ from the three filtration tests was 18.11 ± 0.80 for the feed, whereas for the permeate, OD_600_ was 0.06 ± 0.00. This means that the microbial cells have been totally retained by the membrane and its separation efficiency has not been compromised by the sterilization cycles. The samples were also analyzed by HPLC and the results are presented in Table 5. The composition of the feed is similar to that of the permeate concerning oxalic, malic, acetic, and fumaric acid. However, the mean citric acid concentration was reduced from 6.44 g/L at the feed to 3.73 g/L in the permeate. The difference between the mean values of citric acid concentration between the feed and the permeate are statistically significant, although the molecule size of the citric acid (192 Da) is much smaller than the UF mean pore size; thus, rejection would not have been expected. Adsorption of citric acid on the membrane and its support layer may be a probable explanation for the observed decrease of the citric acid concentration in the permeate sample. However, further investigation is needed to support this hypothesis.

All the aforementioned results suggest that PVDF/polyester hollow-fiber UF membranes, commercially employed in wastewater treatment applications, may also be used in biotechnological applications when heat sterilization is a prerequisite process step. Heat sterilization under typical conditions (i.e., 120 °C, 20 min) does not seem to significantly affect key membrane properties, such as pure water permeance and MWCO, even after repeated (i.e., *n* = 10) sterilization cycles. Although a slight increase of the membrane pore size (and consequently of pure water permeance) is evident in the 1st sterilization cycle, further loosening of the membrane pores does not seem to take place. Moreover, preliminary results from filtration of real fermentation broth verified the rather stable membrane filtration performance regarding both rejection of active cells and membrane fouling propensity.

## 4. Conclusions

Fermentation processes, under sterile conditions, are commonly used for the production of a variety of chemicals. In the case of hybrid biotechnological/membrane processes, the membranes should also be sterilized, commonly through heat sterilization. This study deals comprehensively with the effect of the heat sterilization process (i.e., 120 °C, 20 min) on the filtration performance of a commercial PVDF (active layer)/polyester (supporting layer) hollow-fiber UF membrane. The handmade membrane module was slightly affected by repeated heat sterilization cycles (10 in total), exhibiting practically stable pure water permeance, typical pore size, and fouling performance in filtration tests of both dextran solution and real fermentation broth. The assessment of the commercial PVDF membrane in this work strongly suggests that these membranes can be treated in an autoclave. Therefore, these membranes could be used as a low-cost alternative to high-cost ceramic UF membranes for fermentation processes that require heat sterilization. To the authors’ best knowledge, this is the first time that the effects of the heat sterilization process on commercial PVDF (active layer)/polyester (supporting layer) hollow-fiber UF membranes are systematically assessed, thus paving the way for its application in an integrated MBR system for the selective separation of the products from the fermentation broth as well as for developing a continuous MBR fermentation process.

## Figures and Tables

**Table 1:** A summary of hybrid biotechnological/membrane separation processes and the applied sterilization method. The dashes denote absence of relevant data.

Membrane Type and Configuration	Membrane Material	Membrane Sterilization Method	Bioreactor/Media Sterilization Method	Reference
Hollow fiber sidestream	Polysulfone (PS)	Autoclave sterilization (121 °C, 40 min)	Autoclave sterilization (121 °C, 40 min)	[13]
Microfiltration (MF) hollow fiber sidestream	-	200 ppm NaClO solution	Autoclave sterilization (121 °C, 20 min)	[25]
MF hollow fiber sidestream	Polyethersulfone (PES)	0.2 g/L NaClO solution	Autoclave sterilization (121 °C, 20 min)	[23]
Ultrafiltration (UF) sidestream	Cellulose acetate (CA)	-	Autoclave sterilization (115 °C, 30 min)	[11]
Hollow fiber sidestream	-	-	Autoclave sterilization (121 °C, 15 or 30 min)	[6]
Flat sheet submerged	Polyvinylidene Difluoride (PVDF)	Autoclave sterilization (121 °C, 20 min)	Autoclave sterilization (121 °C, 20 min)	[21,24,26,27]
MF hollow fiber submerged	Polypropylene (PP) PES	In situ sterilization *	Sterilization *	[10,20]
MF hollow fiber sidestream	-	0.2 g/L NaClO solution	Autoclave sterilization (121 °C)	[28]
UF hollow fiber sidestream	PS	0.1 M NaOH and 200 ppm NaOCl	Autoclave sterilization (121 °C, 15 min)	[29]
hollow fiber sidestream	PS	-	Sterilization *	[30]
UF sidestream	Ceramic	-	Sterilization *	[12]
UF hollow fiber submerged	PVDF	1% NaOCl for 18 h	Sterilization *	[22]
MF sidestream	Ceramic	Steam sterilization (121 °C)	Steam sterilization (121 °C)	[7]
UF sidestream	Organic	200 ppm NaClO solution	Steam sterilization (121 °C)	[7]
MF flat sheet sidestream	PVDF	200 ppm NaClO solution + ultrapure water	Autoclave sterilization (121 °C, 15 min)	[31]
UF hollow fiber submerged	Polyamide	5% formaldehyde for 24 h	Sterilization *	[19]

* Sterile conditions are reported but no details are given regarding the sterilization method.

**Table 2:** PVDF thermal properties.

Property	Value
Melting point (°C)	140–170
Glass transition temperature (°C)	−41/−38
Thermal stability, 1% mass loss, in air (°C)	375
Linear thermal expansion coefficient (10−6/°C)	50–103 or 120–140

**Table 3:** Commercial PVDF hollow fiber UF membranes (PURONR MBR, Koch Separation Solutions).

Property	Value
Membrane Chemistry	Proprietary PVDF
Nominal Pore Size	0.03 μm
Outside Fiber Diameter	2.6 mm
Temperature Range	5–40 °C
pH Range for Cleaning	2.0–10.5
Maximum Filtration Trans-membrane Pressure (TMP)	0.6 bar

**Table 4:** Experimental data regarding permeance, molecular weight cut-off (MWCO), and TMP increase rate (ΔTMP/Δt) after each cycle of sterilization. Permeance was determined from clean water flux tests. MWCO and TMP increase rate were estimated from the dextran rejection tests.

No of Ster.	Permeance (LMH/Bar)	MWCO (kDa)	ΔTMP/Δt (Mbar/Min)
0 (initial)	677	42.0	7.0
1	878	98.0	2.7
2	916	43.7	0.7
3	874	44.0	0.7
4	913	31.5	3.0
5	993	36.2	1.2
6	888	49.1	1.0
7	829	79.2	0.7
8	968	76.1	0.8
9	941	86.0	1.1
10	848	40.3	0.5
Average (1–10)	906 ± 49	58.4 ± 22.1	1.3 ± 1.4

**Table 5:** Mean values of optical density (OD_600_) and high-performance liquid chromatography (HPLC) results of the feed and permeate from the three fermentation broth filtration tests at t = 24 h.

Parameter	Feed	Permeate
Oxalic Acid (g/L)	0.00 ± 0.00	0.02 ± 0.01
Citric Acid (g/L)	6.44 ± 0.92 *	3.73 ± 0.54 *
Malic Acid (g/L)	0.97 ± 0.12	0.89 ± 0.08
Acetic Acid (g/L)	0.79 ± 0.10	0.54 ± 0.74
Fumaric Acid (g/L)	0.06 ± 0.01	0.03 ± 0.11
OD (600 nm)	18.11 ± 0.80	0.06 ± 0.00

* The mean difference is significant at the 0.05 level.

## Data Availability

Data are contained as supplementary material; File S1: Figures_data.xlsx.

## Supplementary Materials

The following supporting information can be downloaded at: https://www.mdpi.com/article/10.3390/membranes12080725/s1, File S1: Figures_data.xlsx, Figure S1.pdf.
