# Supplementary material for: The Effect of Heat Sterilization on Key Filtration Performance Parameters of a Commercial Polymeric (PVDF) Hollow-Fiber Ultrafiltration Membrane
Source: Membranes (Basel). 2022 Jul 22;12(8):725. doi: 10.3390/membranes12080725 (PMC9394269; doi:10.3390/membranes12080725)
Supplement: Supplementary file 1 [file membranes-12-00725-s001.zip › membranes-1819687-supplementary.pdf]

membranes

Article

The Effect of Heat Sterilization on Key Filtration Performance Parameters of a Commercial Polymeric (PVDF) Hollow-Fiber Ultrafiltration Membrane

Alexandra Nastouli [1,2], Asimina Tsirigka [1], Michael Harasek [2], Anastasios J. Karabelas [1] and Sotiris I. Patsios [1,*]

[1] Laboratory of Natural Resources and Renewable Energies, Chemical Process & Energy Resources Institute (CPERI), Centre for Research and Technology-Hellas (CERTH), GR 57001 Thessaloniki, Greece; a.nastouli@certh.gr (A.N.); tsirigka@certh.gr (A.T.); karabaj@certh.gr (A.J.K.)

[2] Institute of Chemical, Environmental and Bioscience Engineering, TU Wien, AU 1040 Vienna, Austria; michael.harasek@tuwien.ac.at

* Correspondence: patsios@certh.gr; Tel.: +30 2310498183

Citation: Nastouli, A.; Tsirigka, A.; Harasek, M.; Karabelas, A.J.; Patsios, S.I. The Effect of Heat Sterilization on Key Filtration Performance Parameters of a Commercial Polymeric (PVDF) Hollow-Fiber Ultrafiltration Membrane. *Membranes* **2022**, *12*, x. https://doi.org/10.3390/xxxxx

Academic Editor(s): Xuezhong He

Received: 30 June 2022

Accepted: 13 July 2022

Published: date

Publisher's Note: MDPI stays neutral with regard to jurisdictional claims in published maps and institutional affiliations.

Copyright: © 2022 by the authors. Submitted for possible open access publication under the terms and conditions of the Creative Commons Attribution (CC BY) license (https://creativecommons.org/licenses/by/4.0/).

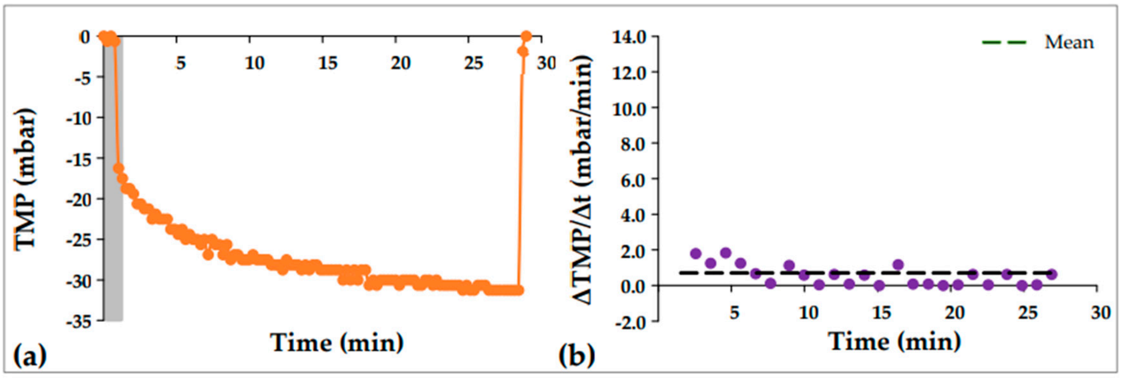

Figure S1. Representation of the RTMP calculation procedure: (**a**) Typical Trans-membrane Pressure (TMP) vs time profile (i.e. 2nd cycle of sterilization); (**b**) Calculated ΔTMP/Δt vs time profile with the mean ΔTMP/Δt value

(a)

Time (min)	ΔP (mbar)
0.00	0
0.25	-1
0.50	0
0.77	-1
1.02	-16
1.28	-18
1.53	-19
1.78	-19
2.03	-19
2.30	-21
2.55	-21
2.80	-21
3.07	-21
3.32	-23
3.57	-22
3.83	-23
4.08	-23
4.35	-23
4.60	-24
4.87	-24
5.12	-24
5.37	-24
5.62	-25
5.88	-24
6.13	-25
6.40	-25
6.65	-26
6.90	-25
7.17	-27
7.42	-25
7.67	-26
7.93	-26
8.18	-27
8.43	-26
8.70	-28
8.95	-27
9.20	-27
9.47	-28
9.72	-28
9.97	-28

(b)

Time (min)	ΔTMP/Δt (mbar/min)	|ΔTMP/Δt| (mbar/min)
1.53	-4.012	4.012
2.55	-1.798	1.798
3.57	-1.251	1.251
4.60	-1.837	1.837
5.62	-1.251	1.251
6.65	-0.664	0.664
7.67	0.117	-0.117
8.95	-1.133	1.133
9.97	-0.586	0.586
11.00	-0.039	0.039
12.02	-0.625	0.625
13.05	-0.078	0.078
14.08	-0.586	0.586
15.10	0.000	0.000
16.40	-1.172	1.172
17.42	-0.078	0.078
18.47	-0.074	0.074
19.48	0.000	0.000
20.50	0.039	-0.039
21.52	-0.625	0.625
22.55	0.039	-0.039
23.83	-0.625	0.625
24.87	0.000	0.000
25.88	-0.039	0.039
26.90	-0.625	0.625
	**Average**	0.7

10.23	-27
10.48	-28
10.73	-28
11.00	-28
11.25	-28
11.50	-28
11.77	-28
12.02	-28
12.27	-29
12.53	-28
12.78	-28
13.05	-28
13.32	-28
13.57	-29
13.82	-28
14.08	-29
14.33	-28
14.60	-28
14.85	-29
15.10	-29
15.37	-29
15.62	-29
15.88	-29
16.13	-29
16.40	-30
16.65	-29
16.90	-30
17.17	-29
17.42	-30
17.67	-29
17.93	-29
18.18	-31
18.47	-30
18.72	-31
18.97	-30
19.22	-30
19.48	-30
19.73	-30
19.98	-30
20.25	-31
20.50	-30
20.75	-30
21.02	-30

21.27	-30
21.52	-31
21.78	-31
22.03	-30
22.28	-30
22.55	-31
22.80	-30
23.05	-31
23.32	-31
23.57	-31
23.83	-31
24.08	-31
24.33	-31
24.60	-31
24.87	-31
25.12	-31
25.37	-31
25.63	-31
25.88	-31
26.13	-31
26.38	-31
26.65	-31
26.90	-31
27.15	-31
27.42	-31
27.67	-31
27.92	-31
28.17	-31
28.43	-31
28.70	-2
28.95	0

Table S2. Experimental data for Figure S1.

membranes MDPI

(a)

Flux (LMH)	STDV	Pressure (bar)	STDV
49.91	0.07	0.00	0.14
41.04	0.06	0.02	0.83
31.35	0.05	0.00	0.03
18.86	0.03	0.00	0.06
10.10	0.02	0.00	0.03

(b)

Flux (LMH)	STDV	Pressure (bar)	STDV
49.98	1.23	0.057	0.04
40.00	0.08	0.046	0.00
30.03	0.03	0.033	0.00
18.72	0.02	0.021	0.01
10.03	0.14	0.011	0.08

(c)

Flux (LMH)	STDV	Pressure (bar)	STDV
47.34	0.00	0.048	0.00
39.62	0.00	0.041	0.00
32.40	0.00	0.034	0.00
19.74	0.00	0.021	0.00
10.35	0.00	0.012	0.00

(d)

Flux (LMH)	STDV	Pressure (bar)	STDV
51.61	0.05	0.052	0.01
40.28	0.04	0.040	0.00
29.72	0.03	0.029	0.00
18.16	0.02	0.017	0.01
9.78	0.01	0.009	0.01

Table S3. Experimental data for Figure 2.

(a)

FEED		PERMEATE	
Time (min)	Intensity	Time (min)	Intensity
0.00	647	0.00	393
0.01	647	0.01	393
0.02	58	0.02	255
0.03	-691	0.03	-754
0.03	-691	0.03	-753
0.04	-691	0.04	-752
0.05	-691	0.05	-752
0.06	-691	0.06	-752
0.07	-691	0.07	-752
0.08	-690	0.08	-752
0.08	-690	0.08	-751
0.09	-690	0.09	-750
0.10	-690	0.10	-750
0.11	-689	0.11	-749
0.12	-689	0.12	-749
0.13	-689	0.13	-749
0.13	-689	0.13	-748
0.14	-690	0.14	-748
0.15	-690	0.15	-748
0.16	-690	0.16	-747
0.17	-689	0.17	-747
0.18	-689	0.18	-747
0.18	-689	0.18	-746
0.19	-688	0.19	-746
0.20	-688	0.20	-746
0.21	-687	0.21	-746
0.22	-686	0.22	-746
0.23	-686	0.23	-745
0.23	-686	0.23	-745
0.24	-685	0.24	-744
0.25	-685	0.25	-744
0.26	-685	0.26	-743
0.27	-685	0.27	-743
0.28	-685	0.28	-743
0.28	-685	0.28	-744
0.29	-685	0.29	-743

(b)

Time (min)	Rejection
22.00	0.10
22.01	0.11
22.02	0.11
22.03	0.12
22.03	0.12
22.04	0.13
22.05	0.13
22.06	0.14
22.07	0.14
22.08	0.15
22.08	0.14
22.09	0.15
22.10	0.15
22.11	0.15
22.12	0.16
22.13	0.16
22.13	0.16
22.14	0.16
22.15	0.17
22.16	0.17
22.17	0.17
22.18	0.18
22.18	0.18
22.19	0.19
22.20	0.19
22.21	0.20
22.22	0.20
22.23	0.21
22.23	0.21
22.24	0.21
22.25	0.22
22.26	0.21
22.27	0.21
22.28	0.22
22.28	0.22
22.29	0.23
22.30	0.23

(c)

MW (kDA)	t (min)
22.8	27.102
47.3	26.035
112	24.771
212	23.848
404	22.894

0.30	-684	0.30	-742	22.31	0.24
0.31	-683	0.31	-742	22.32	0.25
0.32	-683	0.32	-741	22.33	0.25
0.33	-682	0.33	-741	22.33	0.26
0.33	-682	0.33	-741	22.34	0.26
0.34	-682	0.34	-741	22.35	0.27
0.35	-682	0.35	-740	22.36	0.27
0.36	-682	0.36	-740	22.37	0.27
0.37	-681	0.37	-740	22.38	0.27
0.38	-680	0.38	-740	22.38	0.27
0.38	-679	0.38	-739	22.39	0.28
0.39	-679	0.39	-740	22.40	0.28
0.40	-679	0.40	-739	22.41	0.29
0.41	-678	0.41	-739	22.42	0.29
0.42	-678	0.42	-738	22.43	0.29
0.43	-677	0.43	-738	22.43	0.29
0.43	-676	0.43	-738	22.44	0.30
0.44	-676	0.44	-738	22.45	0.30
0.45	-677	0.45	-738	22.46	0.31
0.46	-677	0.46	-738	22.47	0.32
0.47	-678	0.47	-738	22.48	0.32
0.48	-678	0.48	-738	22.48	0.33
0.48	-678	0.48	-738	22.49	0.32
0.49	-677	0.49	-738	22.50	0.33
0.50	-677	0.50	-737	22.51	0.33
0.51	-676	0.51	-736	22.52	0.33
0.52	-676	0.52	-735	22.53	0.33
0.53	-676	0.53	-735	22.53	0.34
0.53	-675	0.53	-735	22.54	0.34
0.54	-675	0.54	-735	22.55	0.34
0.55	-675	0.55	-735	22.56	0.34
0.56	-675	0.56	-735	22.57	0.35
0.57	-675	0.57	-734	22.58	0.35
0.58	-674	0.58	-734	22.58	0.36
0.58	-674	0.58	-733	22.59	0.36
0.59	-674	0.59	-733	22.60	0.36
0.60	-673	0.60	-732	22.61	0.37
0.61	-673	0.61	-731	22.62	0.37
0.62	-672	0.62	-731	22.63	0.37
0.63	-672	0.63	-730	22.63	0.38
0.63	-671	0.63	-730	22.64	0.38
0.64	-671	0.64	-730	22.65	0.38
0.65	-671	0.65	-729	22.66	0.39

0.66	-671	0.66	-728	22.67	0.39
0.67	-671	0.67	-728	22.68	0.39
0.68	-671	0.68	-728	22.68	0.39
0.68	-671	0.68	-728	22.69	0.40
0.69	-671	0.69	-728	22.70	0.40
0.70	-671	0.70	-728	22.71	0.40
0.71	-671	0.71	-728	22.72	0.41
0.72	-671	0.72	-729	22.73	0.41
0.73	-671	0.73	-729	22.73	0.41
0.73	-671	0.73	-728	22.74	0.42
0.74	-670	0.74	-727	22.75	0.42
0.75	-670	0.75	-727	22.76	0.42
0.76	-670	0.76	-727	22.77	0.42
0.77	-669	0.77	-727	22.78	0.42
0.78	-668	0.78	-727	22.78	0.43
0.78	-668	0.78	-726	22.79	0.43
0.79	-668	0.79	-726	22.80	0.43
0.80	-668	0.80	-727	22.81	0.43
0.81	-668	0.81	-727	22.82	0.44
0.82	-668	0.82	-727	22.83	0.44
0.83	-668	0.83	-728	22.83	0.44
0.83	-668	0.83	-728	22.84	0.44
0.84	-668	0.84	-728	22.85	0.45
0.85	-667	0.85	-728	22.86	0.45
0.86	-667	0.86	-727	22.87	0.45
0.87	-667	0.87	-727	22.88	0.46
0.88	-666	0.88	-726	22.88	0.46
0.88	-666	0.88	-726	22.89	0.46
0.89	-666	0.89	-725	22.90	0.46
0.90	-666	0.90	-725	22.91	0.47
0.91	-667	0.91	-724	22.92	0.47
0.92	-667	0.92	-723	22.93	0.47
0.93	-667	0.93	-723	22.93	0.48
0.93	-667	0.93	-723	22.94	0.48
0.94	-667	0.94	-723	22.95	0.48
0.95	-668	0.95	-723	22.96	0.49
0.96	-668	0.96	-723	22.97	0.49
0.97	-669	0.97	-722	22.98	0.49
0.98	-668	0.98	-721	22.98	0.50
0.98	-668	0.98	-720	22.99	0.49
0.99	-668	0.99	-719	23.00	0.49
1.00	-667	1.00	-718	23.01	0.50
1.01	-666	1.01	-718	23.02	0.50

1.02	-665	1.02	-718	23.03	0.50
1.03	-665	1.03	-718	23.03	0.50
1.03	-664	1.03	-717	23.04	0.50
1.04	-664	1.04	-716	23.05	0.51
1.05	-664	1.05	-715	23.06	0.51
1.06	-664	1.06	-715	23.07	0.52
1.07	-663	1.07	-715	23.08	0.52
1.08	-662	1.08	-715	23.08	0.52
1.08	-661	1.08	-716	23.09	0.52
1.09	-660	1.09	-716	23.10	0.53
1.10	-660	1.10	-716	23.11	0.53
1.11	-659	1.11	-715	23.12	0.53
1.12	-659	1.12	-715	23.13	0.53
1.13	-658	1.13	-715	23.13	0.53
1.13	-658	1.13	-716	23.14	0.53
1.14	-657	1.14	-716	23.15	0.54
1.15	-657	1.15	-715	23.16	0.54
1.16	-658	1.16	-715	23.17	0.54
1.17	-658	1.17	-714	23.18	0.54
1.18	-658	1.18	-714	23.18	0.55
1.18	-657	1.18	-713	23.19	0.55
1.19	-656	1.19	-713	23.20	0.55
1.20	-656	1.20	-712	23.21	0.55
1.21	-656	1.21	-711	23.22	0.56
1.22	-656	1.22	-711	23.23	0.56
1.23	-656	1.23	-710	23.23	0.56
1.23	-655	1.23	-711	23.24	0.56
1.24	-655	1.24	-711	23.25	0.56
1.25	-655	1.25	-711	23.26	0.56
1.26	-654	1.26	-711	23.27	0.57
1.27	-654	1.27	-710	23.28	0.57
1.28	-654	1.28	-710	23.28	0.57
1.28	-653	1.28	-709	23.29	0.57
1.29	-653	1.29	-709	23.30	0.57
1.30	-653	1.30	-709	23.31	0.58
1.31	-653	1.31	-709	23.32	0.58
1.32	-653	1.32	-709	23.33	0.58
1.33	-654	1.33	-709	23.33	0.58
1.33	-654	1.33	-710	23.34	0.58
1.34	-654	1.34	-711	23.35	0.58
1.35	-653	1.35	-712	23.36	0.58
1.36	-653	1.36	-713	23.37	0.58
1.37	-653	1.37	-713	23.38	0.58

1.38	-652	1.38	-713	23.38	0.58
1.38	-652	1.38	-713	23.39	0.59
1.39	-652	1.39	-713	23.40	0.59
1.40	-652	1.40	-713	23.41	0.59
1.41	-652	1.41	-712	23.42	0.59
1.42	-652	1.42	-712	23.43	0.59
1.43	-651	1.43	-710	23.43	0.59
1.43	-651	1.43	-709	23.44	0.59
1.44	-650	1.44	-709	23.45	0.59
1.45	-650	1.45	-708	23.46	0.60
1.46	-650	1.46	-707	23.47	0.60
1.47	-649	1.47	-707	23.48	0.60
1.48	-648	1.48	-707	23.48	0.60
1.48	-648	1.48	-708	23.49	0.60
1.49	-647	1.49	-708	23.50	0.61
1.50	-646	1.50	-709	23.51	0.61
1.51	-646	1.51	-709	23.52	0.61
1.52	-645	1.52	-708	23.53	0.61
1.53	-645	1.53	-708	23.53	0.61
1.53	-644	1.53	-708	23.54	0.61
1.54	-644	1.54	-708	23.55	0.62
1.55	-644	1.55	-707	23.56	0.62
1.56	-644	1.56	-707	23.57	0.62
1.57	-644	1.57	-707	23.58	0.62
1.58	-644	1.58	-706	23.58	0.62
1.58	-644	1.58	-706	23.59	0.62
1.59	-644	1.59	-706	23.60	0.61
1.60	-644	1.60	-706	23.61	0.62
1.61	-644	1.61	-706	23.62	0.62
1.62	-644	1.62	-705	23.63	0.62
1.63	-644	1.63	-705	23.63	0.62
1.63	-644	1.63	-705	23.64	0.62
1.64	-643	1.64	-704	23.65	0.62
1.65	-643	1.65	-703	23.66	0.62
1.66	-642	1.66	-703	23.67	0.62
1.67	-642	1.67	-703	23.68	0.62
1.68	-642	1.68	-703	23.68	0.63
1.68	-641	1.68	-702	23.69	0.63
1.69	-641	1.69	-702	23.70	0.63
1.70	-640	1.70	-701	23.71	0.63
1.71	-640	1.71	-700	23.72	0.63
1.72	-640	1.72	-700	23.73	0.63
1.73	-641	1.73	-699	23.73	0.64

1.73	-641	1.73	-698	23.74	0.64
1.74	-641	1.74	-698	23.75	0.64
1.75	-641	1.75	-697	23.76	0.64
1.76	-641	1.76	-697	23.77	0.64
1.77	-640	1.77	-697	23.78	0.64
1.78	-640	1.78	-697	23.78	0.64
1.78	-641	1.78	-698	23.79	0.65
1.79	-640	1.79	-698	23.80	0.65
1.80	-639	1.80	-697	23.81	0.65
1.81	-638	1.81	-697	23.82	0.65
1.82	-638	1.82	-698	23.83	0.65
1.83	-637	1.83	-698	23.83	0.65
1.83	-637	1.83	-698	23.84	0.66
1.84	-637	1.84	-697	23.85	0.66
1.85	-637	1.85	-697	23.86	0.66
1.86	-636	1.86	-697	23.87	0.66
1.87	-636	1.87	-697	23.88	0.66
1.88	-636	1.88	-697	23.88	0.66
1.88	-635	1.88	-696	23.89	0.66
1.89	-635	1.89	-696	23.90	0.67
1.90	-635	1.90	-695	23.91	0.67
1.91	-635	1.91	-694	23.92	0.67
1.92	-634	1.92	-694	23.93	0.67
1.93	-633	1.93	-693	23.93	0.67
1.93	-633	1.93	-693	23.94	0.67
1.94	-632	1.94	-692	23.95	0.68
1.95	-631	1.95	-692	23.96	0.68
1.96	-631	1.96	-691	23.97	0.68
1.97	-630	1.97	-691	23.98	0.68
1.98	-630	1.98	-692	23.98	0.68
1.98	-630	1.98	-692	23.99	0.68
1.99	-630	1.99	-692	24.00	0.68
2.00	-630	2.00	-692	24.01	0.68
2.01	-629	2.01	-692	24.02	0.69
2.02	-629	2.02	-691	24.03	0.69
2.03	-629	2.03	-691	24.03	0.69
2.03	-629	2.03	-690	24.04	0.69
2.04	-629	2.04	-690	24.05	0.69
2.05	-629	2.05	-690	24.06	0.69
2.06	-629	2.06	-691	24.07	0.69
2.07	-629	2.07	-691	24.08	0.69
2.08	-629	2.08	-691	24.08	0.70
2.08	-629	2.08	-691	24.09	0.70

2.09	-629	2.09	-691	24.10	0.70
2.10	-628	2.10	-691	24.11	0.70
2.11	-628	2.11	-690	24.12	0.70
2.12	-628	2.12	-689	24.13	0.70
2.13	-628	2.13	-689	24.13	0.70
2.13	-628	2.13	-690	24.14	0.70
2.14	-627	2.14	-690	24.15	0.71
2.15	-626	2.15	-689	24.16	0.71
2.16	-626	2.16	-689	24.17	0.71
2.17	-625	2.17	-688	24.18	0.71
2.18	-625	2.18	-687	24.18	0.71
2.18	-624	2.18	-687	24.19	0.71
2.19	-624	2.19	-687	24.20	0.71
2.20	-624	2.20	-687	24.21	0.71
2.21	-624	2.21	-687	24.22	0.71
2.22	-623	2.22	-687	24.23	0.71
2.23	-624	2.23	-688	24.23	0.71
2.23	-624	2.23	-688	24.24	0.71
2.24	-624	2.24	-688	24.25	0.71
2.25	-624	2.25	-687	24.26	0.72
2.26	-624	2.26	-687	24.27	0.72
2.27	-624	2.27	-686	24.28	0.72
2.28	-623	2.28	-686	24.28	0.72
2.28	-623	2.28	-685	24.29	0.72
2.29	-623	2.29	-685	24.30	0.72
2.30	-623	2.30	-685	24.31	0.72
2.31	-622	2.31	-684	24.32	0.72
2.32	-623	2.32	-684	24.33	0.72
2.33	-623	2.33	-683	24.33	0.72
2.33	-622	2.33	-683	24.34	0.72
2.34	-621	2.34	-683	24.35	0.72
2.35	-620	2.35	-683	24.36	0.73
2.36	-620	2.36	-683	24.37	0.73
2.37	-619	2.37	-681	24.38	0.73
2.38	-618	2.38	-681	24.38	0.73
2.38	-618	2.38	-680	24.39	0.73
2.39	-619	2.39	-680	24.40	0.73
2.40	-619	2.40	-679	24.41	0.73
2.41	-619	2.41	-678	24.42	0.73
2.42	-619	2.42	-678	24.43	0.73
2.43	-618	2.43	-677	24.43	0.73
2.43	-618	2.43	-677	24.44	0.73
2.44	-618	2.44	-677	24.45	0.74

2.45	-618	2.45	-677	24.46	0.73
2.46	-617	2.46	-677	24.47	0.74
2.47	-617	2.47	-677	24.48	0.74
2.48	-616	2.48	-677	24.48	0.74
2.48	-615	2.48	-676	24.49	0.74
2.49	-615	2.49	-677	24.50	0.74
2.50	-614	2.50	-677	24.51	0.74
2.51	-614	2.51	-676	24.52	0.74
2.52	-614	2.52	-676	24.53	0.74
2.53	-613	2.53	-675	24.53	0.74
2.53	-612	2.53	-675	24.54	0.75
2.54	-612	2.54	-674	24.55	0.75
2.55	-612	2.55	-674	24.56	0.75
2.56	-612	2.56	-673	24.57	0.75
2.57	-612	2.57	-673	24.58	0.75
2.58	-612	2.58	-673	24.58	0.76
2.58	-612	2.58	-672	24.59	0.75
2.59	-612	2.59	-672	24.60	0.76
2.60	-612	2.60	-672	24.61	0.76
2.61	-612	2.61	-671	24.62	0.76
2.62	-612	2.62	-671	24.63	0.76
2.63	-611	2.63	-671	24.63	0.76
2.63	-611	2.63	-671	24.64	0.76
2.64	-610	2.64	-672	24.65	0.76
2.65	-610	2.65	-672	24.66	0.76
2.66	-610	2.66	-672	24.67	0.76
2.67	-610	2.67	-672	24.68	0.76
2.68	-609	2.68	-672	24.68	0.76
2.68	-609	2.68	-671	24.69	0.76
2.69	-608	2.69	-670	24.70	0.76
2.70	-608	2.70	-670	24.71	0.76
2.71	-607	2.71	-669	24.72	0.76
2.72	-607	2.72	-669	24.73	0.77
2.73	-607	2.73	-669	24.73	0.77
2.73	-607	2.73	-668	24.74	0.77
2.74	-607	2.74	-668	24.75	0.77
2.75	-607	2.75	-668	24.76	0.77
2.76	-606	2.76	-669	24.77	0.77
2.77	-606	2.77	-668	24.78	0.77
2.78	-606	2.78	-668	24.78	0.77
2.78	-605	2.78	-668	24.79	0.77
2.79	-605	2.79	-668	24.80	0.77
2.80	-604	2.80	-668	24.81	0.77

2.81	-604	2.81	-667	24.82	0.77
2.82	-604	2.82	-668	24.83	0.77
2.83	-604	2.83	-667	24.83	0.78
2.83	-604	2.83	-667	24.84	0.78
2.84	-604	2.84	-667	24.85	0.78
2.85	-604	2.85	-666	24.86	0.78
2.86	-604	2.86	-666	24.87	0.78
2.87	-604	2.87	-665	24.88	0.78
2.88	-604	2.88	-665	24.88	0.78
2.88	-604	2.88	-664	24.89	0.78
2.89	-603	2.89	-664	24.90	0.78
2.90	-602	2.90	-664	24.91	0.79
2.91	-601	2.91	-664	24.92	0.79
2.92	-601	2.92	-664	24.93	0.79
2.93	-600	2.93	-664	24.93	0.79
2.93	-600	2.93	-664	24.94	0.79
2.94	-600	2.94	-664	24.95	0.79
2.95	-599	2.95	-663	24.96	0.79
2.96	-599	2.96	-663	24.97	0.79
2.97	-599	2.97	-662	24.98	0.79
2.98	-598	2.98	-662	24.98	0.79
2.98	-598	2.98	-662	24.99	0.79
2.99	-597	2.99	-661	25.00	0.80
3.00	-597	3.00	-661	25.01	0.80
3.01	-597	3.01	-660	25.02	0.80
3.02	-598	3.02	-659	25.03	0.80
3.03	-598	3.03	-659	25.03	0.80
3.03	-598	3.03	-659	25.04	0.80
3.04	-598	3.04	-659	25.05	0.80
3.05	-598	3.05	-659	25.06	0.80
3.06	-598	3.06	-659	25.07	0.80
3.07	-598	3.07	-660	25.08	0.80
3.08	-597	3.08	-660	25.08	0.80
3.08	-597	3.08	-661	25.09	0.80
3.09	-597	3.09	-660	25.10	0.81
3.10	-597	3.10	-659	25.11	0.81
3.11	-596	3.11	-659	25.12	0.81
3.12	-595	3.12	-659	25.13	0.81
3.13	-595	3.13	-659	25.13	0.81
3.13	-595	3.13	-659	25.14	0.81
3.14	-595	3.14	-659	25.15	0.81
3.15	-595	3.15	-658	25.16	0.81
3.16	-594	3.16	-658	25.17	0.81

3.17	-594	3.17	-658	25.18	0.81
3.18	-593	3.18	-657	25.18	0.82
3.18	-592	3.18	-657	25.19	0.82
3.19	-592	3.19	-657	25.20	0.82
3.20	-591	3.20	-657	25.21	0.82
3.21	-591	3.21	-656	25.22	0.82
3.22	-591	3.22	-656	25.23	0.82
3.23	-591	3.23	-655	25.23	0.82
3.23	-591	3.23	-655	25.24	0.82
3.24	-592	3.24	-655	25.25	0.82
3.25	-592	3.25	-655	25.26	0.82
3.26	-592	3.26	-655	25.27	0.82
3.27	-592	3.27	-654	25.28	0.82
3.28	-592	3.28	-654	25.28	0.82
3.28	-592	3.28	-653	25.29	0.83
3.29	-592	3.29	-653	25.30	0.83
3.30	-591	3.30	-652	25.31	0.83
3.31	-591	3.31	-652	25.32	0.83
3.32	-591	3.32	-652	25.33	0.83
3.33	-590	3.33	-652	25.33	0.83
3.33	-591	3.33	-652	25.34	0.83
3.34	-590	3.34	-651	25.35	0.83
3.35	-589	3.35	-650	25.36	0.83
3.36	-588	3.36	-650	25.37	0.84
3.37	-588	3.37	-650	25.38	0.84
3.38	-588	3.38	-649	25.38	0.84
3.38	-588	3.38	-648	25.39	0.84
3.39	-588	3.39	-648	25.40	0.84
3.40	-588	3.40	-647	25.41	0.84
3.41	-588	3.41	-647	25.42	0.84
3.42	-588	3.42	-647	25.43	0.84
3.43	-588	3.43	-647	25.43	0.84
3.43	-588	3.43	-648	25.44	0.84
3.44	-588	3.44	-648	25.45	0.84
3.45	-588	3.45	-648	25.46	0.84
3.46	-587	3.46	-648	25.47	0.84
3.47	-587	3.47	-648	25.48	0.84
3.48	-587	3.48	-648	25.48	0.84
3.48	-586	3.48	-648	25.49	0.84
3.49	-586	3.49	-648	25.50	0.84
3.50	-585	3.50	-647	25.51	0.84
3.51	-584	3.51	-646	25.52	0.84
3.52	-584	3.52	-645	25.53	0.84

3.53	-584	3.53	-645	25.53	0.84
3.53	-584	3.53	-645	25.54	0.84
3.54	-583	3.54	-645	25.55	0.84
3.55	-583	3.55	-646	25.56	0.84
3.56	-582	3.56	-646	25.57	0.84
3.57	-581	3.57	-646	25.58	0.84
3.58	-581	3.58	-646	25.58	0.84
3.58	-581	3.58	-645	25.59	0.84
3.59	-580	3.59	-645	25.60	0.84
3.60	-580	3.60	-645	25.61	0.84
3.61	-580	3.61	-646	25.62	0.85
3.62	-579	3.62	-646	25.63	0.85
3.63	-579	3.63	-645	25.63	0.85
3.63	-579	3.63	-645	25.64	0.85
3.64	-579	3.64	-645	25.65	0.85
3.65	-578	3.65	-644	25.66	0.85
3.66	-579	3.66	-644	25.67	0.85
3.67	-579	3.67	-644	25.68	0.85
3.68	-580	3.68	-644	25.68	0.85
3.68	-580	3.68	-643	25.69	0.85
3.69	-580	3.69	-643	25.70	0.85
3.70	-579	3.70	-644	25.71	0.85
3.71	-579	3.71	-644	25.72	0.85
3.72	-578	3.72	-644	25.73	0.85
3.73	-577	3.73	-644	25.73	0.85
3.73	-577	3.73	-645	25.74	0.85
3.74	-577	3.74	-646	25.75	0.85
3.75	-577	3.75	-646	25.76	0.86
3.76	-576	3.76	-646	25.77	0.85
3.77	-576	3.77	-646	25.78	0.85
3.78	-575	3.78	-646	25.78	0.85
3.78	-575	3.78	-645	25.79	0.86
3.79	-574	3.79	-644	25.80	0.86
3.80	-574	3.80	-644	25.81	0.86
3.81	-575	3.81	-643	25.82	0.86
3.82	-574	3.82	-643	25.83	0.86
3.83	-574	3.83	-644	25.83	0.86
3.83	-573	3.83	-644	25.84	0.86
3.84	-572	3.84	-644	25.85	0.86
3.85	-573	3.85	-644	25.86	0.87
3.86	-573	3.86	-644	25.87	0.87
3.87	-573	3.87	-643	25.88	0.87
3.88	-573	3.88	-644	25.88	0.87

3.88	-572	3.88	-644	25.89	0.87
3.89	-572	3.89	-643	25.90	0.87
3.90	-572	3.90	-643	25.91	0.87
3.91	-572	3.91	-643	25.92	0.87
3.92	-571	3.92	-642	25.93	0.87
3.93	-571	3.93	-642	25.93	0.87
3.93	-571	3.93	-641	25.94	0.87
3.94	-570	3.94	-641	25.95	0.87
3.95	-570	3.95	-640	25.96	0.87
3.96	-570	3.96	-640	25.97	0.87
3.97	-569	3.97	-639	25.98	0.87
3.98	-569	3.98	-639	25.98	0.87
3.98	-569	3.98	-638	25.99	0.87
3.99	-569	3.99	-639	26.00	0.88
4.00	-569	4.00	-639	26.01	0.88
4.01	-568	4.01	-639	26.02	0.88
4.02	-568	4.02	-639	26.03	0.88
4.03	-568	4.03	-640	26.03	0.88
4.03	-567	4.03	-639	26.04	0.88
4.04	-567	4.04	-640	26.05	0.88
4.05	-568	4.05	-640	26.06	0.88
4.06	-568	4.06	-639	26.07	0.88
4.07	-568	4.07	-639	26.08	0.88
4.08	-567	4.08	-639	26.08	0.89
4.08	-567	4.08	-639	26.09	0.89
4.09	-567	4.09	-639	26.10	0.89
4.10	-567	4.10	-639	26.11	0.89
4.11	-567	4.11	-638	26.12	0.89
4.12	-567	4.12	-638	26.13	0.89
4.13	-566	4.13	-637	26.13	0.89
4.13	-566	4.13	-636	26.14	0.89
4.14	-565	4.14	-635	26.15	0.90
4.15	-565	4.15	-635	26.16	0.90
4.16	-565	4.16	-634	26.17	0.90
4.17	-564	4.17	-633	26.18	0.90
4.18	-564	4.18	-632	26.18	0.90
4.18	-564	4.18	-632	26.19	0.90
4.19	-564	4.19	-632	26.20	0.90
4.20	-563	4.20	-632	26.21	0.90
4.21	-563	4.21	-632	26.22	0.90
4.22	-563	4.22	-632	26.23	0.90
4.23	-563	4.23	-632	26.23	0.90
4.23	-563	4.23	-631	26.24	0.90

4.24	-563	4.24	-631	26.25	0.90
4.25	-563	4.25	-631	26.26	0.90
4.26	-563	4.26	-632	26.27	0.90
4.27	-562	4.27	-632	26.28	0.90
4.28	-561	4.28	-632	26.28	0.90
4.28	-561	4.28	-632	26.29	0.91
4.29	-561	4.29	-633	26.30	0.91
4.30	-561	4.30	-632	26.31	0.91
4.31	-562	4.31	-632	26.32	0.91
4.32	-561	4.32	-632	26.33	0.91
4.33	-561	4.33	-631	26.33	0.91
4.33	-560	4.33	-631	26.34	0.91
4.34	-560	4.34	-630	26.35	0.91
4.35	-560	4.35	-629	26.36	0.91
4.36	-559	4.36	-629	26.37	0.91
4.37	-559	4.37	-629	26.38	0.92
4.38	-558	4.38	-628	26.38	0.92
4.38	-558	4.38	-628	26.39	0.92
4.39	-558	4.39	-628	26.40	0.92
4.40	-558	4.40	-628	26.41	0.92
4.41	-557	4.41	-628	26.42	0.92
4.42	-557	4.42	-627	26.43	0.92
4.43	-557	4.43	-626	26.43	0.92
4.43	-558	4.43	-626	26.44	0.92
4.44	-558	4.44	-626	26.45	0.92
4.45	-558	4.45	-626	26.46	0.92
4.46	-557	4.46	-626	26.47	0.92
4.47	-557	4.47	-626	26.48	0.92
4.48	-557	4.48	-625	26.48	0.93
4.48	-557	4.48	-624	26.49	0.93
4.49	-557	4.49	-623	26.50	0.93
4.50	-557	4.50	-622	26.51	0.93
4.51	-557	4.51	-623	26.52	0.93
4.52	-556	4.52	-623	26.53	0.93
4.53	-557	4.53	-623	26.53	0.93
4.53	-556	4.53	-623	26.54	0.93
4.54	-556	4.54	-623	26.55	0.93
4.55	-556	4.55	-623	26.56	0.93
4.56	-556	4.56	-623	26.57	0.93
4.57	-556	4.57	-623	26.58	0.93
4.58	-556	4.58	-622	26.58	0.93
4.58	-555	4.58	-621	26.59	0.93
4.59	-555	4.59	-621	26.60	0.93

4.60	-555	4.60	-620	26.61	0.93
4.61	-555	4.61	-620	26.62	0.93
4.62	-553	4.62	-619	26.63	0.93
4.63	-552	4.63	-617	26.63	0.93
4.63	-551	4.63	-616	26.64	0.93
4.64	-550	4.64	-616	26.65	0.93
4.65	-549	4.65	-616	26.66	0.93
4.66	-549	4.66	-616	26.67	0.93
4.67	-548	4.67	-616	26.68	0.93
4.68	-548	4.68	-617	26.68	0.93
4.68	-548	4.68	-617	26.69	0.94
4.69	-548	4.69	-616	26.70	0.94
4.70	-548	4.70	-616	26.71	0.94
4.71	-547	4.71	-615	26.72	0.94
4.72	-546	4.72	-616	26.73	0.94
4.73	-545	4.73	-616	26.73	0.94
4.73	-545	4.73	-616	26.74	0.94
4.74	-545	4.74	-616	26.75	0.94
4.75	-545	4.75	-615	26.76	0.94
4.76	-544	4.76	-615	26.77	0.94
4.77	-544	4.77	-614	26.78	0.95
4.78	-543	4.78	-614	26.78	0.95
4.78	-542	4.78	-614	26.79	0.95
4.79	-541	4.79	-613	26.80	0.95
4.80	-541	4.80	-613	26.81	0.95
4.81	-540	4.81	-613	26.82	0.95
4.82	-540	4.82	-613	26.83	0.95
4.83	-540	4.83	-612	26.83	0.95
4.83	-540	4.83	-612	26.84	0.95
4.84	-540	4.84	-612	26.85	0.95
4.85	-540	4.85	-612	26.86	0.95
4.86	-541	4.86	-612	26.87	0.95
4.87	-541	4.87	-612	26.88	0.95
4.88	-540	4.88	-613	26.88	0.95
4.88	-539	4.88	-613	26.89	0.95
4.89	-538	4.89	-613	26.90	0.95
4.90	-538	4.90	-613	26.91	0.95
4.91	-538	4.91	-613	26.92	0.96
4.92	-538	4.92	-613	26.93	0.96
4.93	-538	4.93	-613	26.93	0.96
4.93	-538	4.93	-613	26.94	0.96
4.94	-537	4.94	-612	26.95	0.96
4.95	-537	4.95	-612	26.96	0.96

4.96	-536	4.96	-612	26.97	0.96
4.97	-536	4.97	-612	26.98	0.96
4.98	-536	4.98	-612	26.98	0.96
4.98	-535	4.98	-612	26.99	0.96
4.99	-535	4.99	-612	27.00	0.96
5.00	-535	5.00	-612	27.01	0.96
5.01	-535	5.01	-611	27.02	0.96
5.02	-534	5.02	-611	27.03	0.97
5.03	-534	5.03	-610	27.03	0.97
5.03	-534	5.03	-610	27.04	0.97
5.04	-534	5.04	-610	27.05	0.97
5.05	-534	5.05	-609	27.06	0.97
5.06	-533	5.06	-609	27.07	0.97
5.07	-533	5.07	-608	27.08	0.97
5.08	-533	5.08	-607	27.08	0.97
5.08	-532	5.08	-607	27.09	0.97
5.09	-532	5.09	-607	27.10	0.97
5.10	-531	5.10	-607	27.11	0.97
5.11	-531	5.11	-607	27.12	0.97
5.12	-530	5.12	-607	27.13	0.97
5.13	-530	5.13	-607	27.13	0.97
5.13	-530	5.13	-607	27.14	0.97
5.14	-531	5.14	-606	27.15	0.97
5.15	-530	5.15	-606	27.16	0.97
5.16	-530	5.16	-606	27.17	0.97
5.17	-529	5.17	-605	27.18	0.97
5.18	-528	5.18	-605	27.18	0.97
5.18	-527	5.18	-604	27.19	0.97
5.19	-526	5.19	-604	27.20	0.98
5.20	-526	5.20	-603	27.21	0.98
5.21	-526	5.21	-603	27.22	0.98
5.22	-526	5.22	-603	27.23	0.98
5.23	-526	5.23	-602	27.23	0.98
5.23	-526	5.23	-603	27.24	0.98
5.24	-527	5.24	-603	27.25	0.98
5.25	-527	5.25	-603	27.26	0.98
5.26	-527	5.26	-602	27.27	0.98
5.27	-527	5.27	-601	27.28	0.98
5.28	-527	5.28	-601	27.28	0.98
5.28	-527	5.28	-601	27.29	0.98
5.29	-526	5.29	-600	27.30	0.98
5.30	-526	5.30	-600	27.31	0.98
5.31	-526	5.31	-600	27.32	0.99

5.32	-525	5.32	-601	27.33	0.99
5.33	-524	5.33	-600	27.33	0.99
5.33	-524	5.33	-600	27.34	0.99
5.34	-523	5.34	-599	27.35	0.99
5.35	-524	5.35	-600	27.36	0.99
5.36	-524	5.36	-600	27.37	0.99
5.37	-523	5.37	-599	27.38	0.99
5.38	-523	5.38	-599	27.38	0.99
5.38	-522	5.38	-598	27.39	0.99
5.39	-522	5.39	-598	27.40	0.99
5.40	-522	5.40	-598	27.41	0.99
5.41	-522	5.41	-599	27.42	0.99
5.42	-521	5.42	-599	27.43	0.99
5.43	-521	5.43	-598	27.43	1.00
5.43	-521	5.43	-598	27.44	1.00
5.44	-522	5.44	-598	27.45	1.00
5.45	-521	5.45	-597	27.46	1.00
5.46	-521	5.46	-597	27.47	1.00
5.47	-521	5.47	-596	27.48	1.00
5.48	-521	5.48	-596	27.48	1.00
5.48	-521	5.48	-596	27.49	1.00
5.49	-521	5.49	-595	27.50	1.00
5.50	-521	5.50	-595	27.51	1.00
5.51	-520	5.51	-595	27.52	1.00
5.52	-519	5.52	-594	27.53	1.00
5.53	-519	5.53	-594	27.53	1.00
5.53	-519	5.53	-594	27.54	1.00
5.54	-518	5.54	-594	27.55	1.00
5.55	-519	5.55	-593	27.56	1.00
5.56	-518	5.56	-593	27.57	1.00
5.57	-518	5.57	-592	27.58	1.00
5.58	-517	5.58	-592	27.58	1.00
5.58	-517	5.58	-591	27.59	1.00
5.59	-517	5.59	-590	27.60	1.01
5.60	-517	5.60	-589	27.61	1.01
5.61	-516	5.61	-588	27.62	1.01
5.62	-516	5.62	-588	27.63	1.01
5.63	-515	5.63	-588	27.63	1.01
5.63	-514	5.63	-588	27.64	1.01
5.64	-513	5.64	-588	27.65	1.01
5.65	-513	5.65	-588	27.66	1.01
5.66	-513	5.66	-587	27.67	1.01
5.67	-513	5.67	-586	27.68	1.01

5.68	-513	5.68	-585	27.68	1.01
5.68	-513	5.68	-584	27.69	1.01
5.69	-513	5.69	-584	27.70	1.01
5.70	-513	5.70	-584	27.71	1.01
5.71	-513	5.71	-584	27.72	1.01
5.72	-513	5.72	-584	27.73	1.01
5.73	-513	5.73	-584	27.73	1.01
5.73	-513	5.73	-584	27.74	1.01
5.74	-513	5.74	-584	27.75	1.01
5.75	-513	5.75	-584	27.76	1.01
5.76	-513	5.76	-584	27.77	1.01
5.77	-512	5.77	-585	27.78	1.01
5.78	-512	5.78	-585	27.78	1.01
5.78	-511	5.78	-584	27.79	1.01
5.79	-511	5.79	-583	27.80	1.01
5.80	-511	5.80	-583	27.81	1.01
5.81	-510	5.81	-583	27.82	1.01
5.82	-509	5.82	-582	27.83	1.01
5.83	-508	5.83	-581	27.83	1.01
5.83	-508	5.83	-581	27.84	1.01
5.84	-507	5.84	-581	27.85	1.01
5.85	-506	5.85	-581	27.86	1.01
5.86	-505	5.86	-581	27.87	1.02
5.87	-505	5.87	-582	27.88	1.02
5.88	-504	5.88	-581	27.88	1.02
5.88	-504	5.88	-581	27.89	1.02
5.89	-504	5.89	-580	27.90	1.02
5.90	-504	5.90	-580	27.91	1.02
5.91	-503	5.91	-580	27.92	1.01
5.92	-503	5.92	-580	27.93	1.01
5.93	-503	5.93	-580	27.93	1.02
5.93	-503	5.93	-580	27.94	1.02
5.94	-503	5.94	-579	27.95	1.02
5.95	-503	5.95	-578	27.96	1.02
5.96	-502	5.96	-578	27.97	1.02
5.97	-502	5.97	-578	27.98	1.02
5.98	-502	5.98	-578	27.98	1.02
5.98	-502	5.98	-578	27.99	1.02
5.99	-501	5.99	-578	28.00	1.02
6.00	-501	6.00	-577		
6.01	-501	6.01	-577		
6.02	-501	6.02	-577		
6.03	-500	6.03	-577		

6.03	-500	6.03	-577
6.04	-499	6.04	-577
6.05	-498	6.05	-576
6.06	-498	6.06	-575
6.07	-497	6.07	-575
6.08	-496	6.08	-575
6.08	-496	6.08	-574
6.09	-496	6.09	-574
6.10	-496	6.10	-573
6.11	-496	6.11	-572
6.12	-496	6.12	-572
6.13	-495	6.13	-572
6.13	-495	6.13	-571
6.14	-496	6.14	-570
6.15	-496	6.15	-570
6.16	-495	6.16	-570
6.17	-495	6.17	-570
6.18	-494	6.18	-571
6.18	-494	6.18	-571
6.19	-493	6.19	-570
6.20	-493	6.20	-570
6.21	-493	6.21	-569
6.22	-493	6.22	-569
6.23	-493	6.23	-568
6.23	-493	6.23	-568
6.24	-493	6.24	-568
6.25	-493	6.25	-568
6.26	-493	6.26	-568
6.27	-493	6.27	-568
6.28	-493	6.28	-567
6.28	-493	6.28	-567
6.29	-493	6.29	-567
6.30	-492	6.30	-567
6.31	-492	6.31	-567
6.32	-491	6.32	-568
6.33	-491	6.33	-568
6.33	-491	6.33	-568
6.34	-491	6.34	-567
6.35	-491	6.35	-567
6.36	-490	6.36	-567
6.37	-490	6.37	-567
6.38	-490	6.38	-566
6.38	-489	6.38	-566

6.39	-488	6.39	-565
6.40	-488	6.40	-565
6.41	-487	6.41	-565
6.42	-486	6.42	-565
6.43	-486	6.43	-565
6.43	-486	6.43	-564
6.44	-486	6.44	-564
6.45	-485	6.45	-564
6.46	-486	6.46	-563
6.47	-486	6.47	-563
6.48	-486	6.48	-563
6.48	-485	6.48	-562
6.49	-485	6.49	-562
6.50	-484	6.50	-561
6.51	-483	6.51	-561
6.52	-482	6.52	-562
6.53	-482	6.53	-561
6.53	-482	6.53	-561
6.54	-482	6.54	-560
6.55	-482	6.55	-560
6.56	-482	6.56	-559
6.57	-482	6.57	-560
6.58	-481	6.58	-560
6.58	-481	6.58	-560
6.59	-480	6.59	-560
6.60	-481	6.60	-560
6.61	-481	6.61	-560
6.62	-480	6.62	-560
6.63	-480	6.63	-559
6.63	-479	6.63	-560
6.64	-479	6.64	-559
6.65	-479	6.65	-559
6.66	-479	6.66	-559
6.67	-479	6.67	-560
6.68	-480	6.68	-560
6.68	-479	6.68	-559
6.69	-479	6.69	-558
6.70	-479	6.70	-558
6.71	-480	6.71	-558
6.72	-480	6.72	-558
6.73	-479	6.73	-559
6.73	-479	6.73	-559
6.74	-478	6.74	-559

6.75	-479	6.75	-558
6.76	-479	6.76	-557
6.77	-479	6.77	-556
6.78	-478	6.78	-555
6.78	-478	6.78	-555
6.79	-477	6.79	-555
6.80	-476	6.80	-555
6.81	-476	6.81	-555
6.82	-475	6.82	-555
6.83	-475	6.83	-555
6.83	-474	6.83	-554
6.84	-474	6.84	-553
6.85	-473	6.85	-553
6.86	-473	6.86	-553
6.87	-473	6.87	-553
6.88	-473	6.88	-553
6.88	-472	6.88	-553
6.89	-472	6.89	-552
6.90	-471	6.90	-552
6.91	-471	6.91	-552
6.92	-470	6.92	-552
6.93	-470	6.93	-552
6.93	-470	6.93	-552
6.94	-469	6.94	-552
6.95	-470	6.95	-552
6.96	-470	6.96	-552
6.97	-470	6.97	-552
6.98	-470	6.98	-552
6.98	-470	6.98	-553
6.99	-470	6.99	-552
7.00	-469	7.00	-552
7.01	-469	7.01	-552
7.02	-468	7.02	-551
7.03	-467	7.03	-551
7.03	-466	7.03	-550
7.04	-466	7.04	-550
7.05	-466	7.05	-550
7.06	-466	7.06	-550
7.07	-466	7.07	-549
7.08	-466	7.08	-549
7.08	-466	7.08	-549
7.09	-467	7.09	-548
7.10	-467	7.10	-548

7.11	-467	7.11	-548
7.12	-467	7.12	-547
7.13	-466	7.13	-547
7.13	-465	7.13	-547
7.14	-465	7.14	-548
7.15	-464	7.15	-548
7.16	-463	7.16	-548
7.17	-463	7.17	-548
7.18	-462	7.18	-548
7.18	-462	7.18	-548
7.19	-461	7.19	-548
7.20	-461	7.20	-548
7.21	-461	7.21	-547
7.22	-461	7.22	-546
7.23	-460	7.23	-545
7.23	-460	7.23	-545
7.24	-459	7.24	-545
7.25	-458	7.25	-544
7.26	-458	7.26	-544
7.27	-458	7.27	-544
7.28	-458	7.28	-543
7.28	-458	7.28	-543
7.29	-457	7.29	-543
7.30	-457	7.30	-542
7.31	-457	7.31	-542
7.32	-457	7.32	-541
7.33	-456	7.33	-541
7.33	-456	7.33	-541
7.34	-456	7.34	-541
7.35	-455	7.35	-541
7.36	-455	7.36	-540
7.37	-455	7.37	-540
7.38	-454	7.38	-540
7.38	-454	7.38	-540
7.39	-453	7.39	-540
7.40	-453	7.40	-540
7.41	-453	7.41	-540
7.42	-452	7.42	-539
7.43	-452	7.43	-539
7.43	-453	7.43	-539
7.44	-453	7.44	-539
7.45	-453	7.45	-539
7.46	-452	7.46	-538

7.47	-451	7.47	-538
7.48	-451	7.48	-537
7.48	-451	7.48	-537
7.49	-450	7.49	-537
7.50	-451	7.50	-537
7.51	-451	7.51	-538
7.52	-451	7.52	-538
7.53	-451	7.53	-538
7.53	-450	7.53	-538
7.54	-450	7.54	-538
7.55	-449	7.55	-537
7.56	-449	7.56	-536
7.57	-449	7.57	-536
7.58	-449	7.58	-536
7.58	-449	7.58	-535
7.59	-449	7.59	-535
7.60	-448	7.60	-534
7.61	-448	7.61	-533
7.62	-448	7.62	-533
7.63	-448	7.63	-533
7.63	-447	7.63	-533
7.64	-447	7.64	-533
7.65	-446	7.65	-533
7.66	-447	7.66	-533
7.67	-447	7.67	-532
7.68	-446	7.68	-531
7.68	-446	7.68	-531
7.69	-446	7.69	-531
7.70	-447	7.70	-531
7.71	-446	7.71	-530
7.72	-446	7.72	-530
7.73	-446	7.73	-530
7.73	-446	7.73	-530
7.74	-446	7.74	-530
7.75	-446	7.75	-529
7.76	-445	7.76	-529
7.77	-444	7.77	-528
7.78	-444	7.78	-527
7.78	-444	7.78	-527
7.79	-444	7.79	-526
7.80	-444	7.80	-525
7.81	-444	7.81	-524
7.82	-444	7.82	-524

7.83	-443	7.83	-524
7.83	-444	7.83	-524
7.84	-444	7.84	-523
7.85	-443	7.85	-524
7.86	-442	7.86	-524
7.87	-442	7.87	-524
7.88	-441	7.88	-524
7.88	-441	7.88	-523
7.89	-441	7.89	-522
7.90	-441	7.90	-522
7.91	-441	7.91	-521
7.92	-441	7.92	-521
7.93	-440	7.93	-521
7.93	-440	7.93	-521
7.94	-440	7.94	-521
7.95	-440	7.95	-521
7.96	-439	7.96	-521
7.97	-439	7.97	-521
7.98	-438	7.98	-521
7.98	-438	7.98	-520
7.99	-438	7.99	-518
8.00	-437	8.00	-517
8.01	-437	8.01	-517
8.02	-437	8.02	-517
8.03	-436	8.03	-517
8.03	-436	8.03	-517
8.04	-435	8.04	-517
8.05	-434	8.05	-517
8.06	-434	8.06	-517
8.07	-433	8.07	-518
8.08	-433	8.08	-518
8.08	-434	8.08	-518
8.09	-434	8.09	-518
8.10	-433	8.10	-518
8.11	-433	8.11	-518
8.12	-433	8.12	-518
8.13	-434	8.13	-519
8.13	-434	8.13	-519
8.14	-433	8.14	-520
8.15	-433	8.15	-520
8.16	-432	8.16	-520
8.17	-431	8.17	-520
8.18	-430	8.18	-520

8.18	-430	8.18	-519
8.19	-430	8.19	-518
8.20	-430	8.20	-518
8.21	-429	8.21	-518
8.22	-429	8.22	-517
8.23	-428	8.23	-517
8.23	-428	8.23	-516
8.24	-428	8.24	-515
8.25	-427	8.25	-515
8.26	-427	8.26	-515
8.27	-427	8.27	-515
8.28	-427	8.28	-515
8.28	-427	8.28	-515
8.29	-426	8.29	-515
8.30	-426	8.30	-516
8.31	-426	8.31	-516
8.32	-426	8.32	-515
8.33	-426	8.33	-515
8.33	-426	8.33	-514
8.34	-426	8.34	-514
8.35	-425	8.35	-514
8.36	-425	8.36	-514
8.37	-425	8.37	-514
8.38	-425	8.38	-514
8.38	-425	8.38	-514
8.39	-425	8.39	-514
8.40	-425	8.40	-513
8.41	-424	8.41	-512
8.42	-424	8.42	-512
8.43	-423	8.43	-511
8.43	-422	8.43	-510
8.44	-421	8.44	-510
8.45	-420	8.45	-509
8.46	-419	8.46	-508
8.47	-419	8.47	-507
8.48	-419	8.48	-506
8.48	-419	8.48	-506
8.49	-419	8.49	-506
8.50	-418	8.50	-506
8.51	-418	8.51	-505
8.52	-418	8.52	-505
8.53	-418	8.53	-505
8.53	-418	8.53	-505

8.54	-418	8.54	-504
8.55	-418	8.55	-503
8.56	-418	8.56	-503
8.57	-417	8.57	-503
8.58	-417	8.58	-503
8.58	-416	8.58	-502
8.59	-415	8.59	-502
8.60	-415	8.60	-503
8.61	-415	8.61	-503
8.62	-415	8.62	-502
8.63	-414	8.63	-502
8.63	-414	8.63	-501
8.64	-413	8.64	-500
8.65	-413	8.65	-500
8.66	-412	8.66	-499
8.67	-412	8.67	-499
8.68	-412	8.68	-499
8.68	-412	8.68	-499
8.69	-412	8.69	-498
8.70	-412	8.70	-498
8.71	-412	8.71	-498
8.72	-413	8.72	-498
8.73	-413	8.73	-498
8.73	-413	8.73	-498
8.74	-413	8.74	-498
8.75	-413	8.75	-497
8.76	-412	8.76	-497
8.77	-412	8.77	-496
8.78	-412	8.78	-496
8.78	-411	8.78	-496
8.79	-411	8.79	-496
8.80	-410	8.80	-495
8.81	-409	8.81	-495
8.82	-409	8.82	-496
8.83	-409	8.83	-496
8.83	-409	8.83	-496
8.84	-409	8.84	-496
8.85	-409	8.85	-496
8.86	-408	8.86	-496
8.87	-408	8.87	-496
8.88	-407	8.88	-495
8.88	-407	8.88	-495
8.89	-406	8.89	-494

8.90	-406	8.90	-493
8.91	-406	8.91	-492
8.92	-406	8.92	-491
8.93	-406	8.93	-492
8.93	-407	8.93	-492
8.94	-407	8.94	-492
8.95	-407	8.95	-493
8.96	-406	8.96	-493
8.97	-406	8.97	-493
8.98	-406	8.98	-493
8.98	-405	8.98	-493
8.99	-404	8.99	-492
9.00	-404	9.00	-492
9.01	-403	9.01	-492
9.02	-403	9.02	-492
9.03	-402	9.03	-492
9.03	-401	9.03	-492
9.04	-401	9.04	-492
9.05	-401	9.05	-491
9.06	-400	9.06	-492
9.07	-399	9.07	-492
9.08	-398	9.08	-492
9.08	-397	9.08	-491
9.09	-397	9.09	-491
9.10	-397	9.10	-491
9.11	-397	9.11	-491
9.12	-397	9.12	-491
9.13	-397	9.13	-490
9.13	-397	9.13	-490
9.14	-396	9.14	-489
9.15	-396	9.15	-489
9.16	-395	9.16	-489
9.17	-394	9.17	-489
9.18	-393	9.18	-489
9.18	-392	9.18	-489
9.19	-391	9.19	-489
9.20	-391	9.20	-489
9.21	-391	9.21	-488
9.22	-391	9.22	-488
9.23	-390	9.23	-488
9.23	-390	9.23	-488
9.24	-389	9.24	-488
9.25	-389	9.25	-488

9.26	-389	9.26	-488
9.27	-389	9.27	-488
9.28	-389	9.28	-488
9.28	-388	9.28	-487
9.29	-388	9.29	-487
9.30	-389	9.30	-487
9.31	-389	9.31	-487
9.32	-389	9.32	-486
9.33	-389	9.33	-486
9.33	-388	9.33	-485
9.34	-388	9.34	-485
9.35	-388	9.35	-485
9.36	-388	9.36	-485
9.37	-388	9.37	-485
9.38	-388	9.38	-484
9.38	-388	9.38	-483
9.39	-388	9.39	-483
9.40	-388	9.40	-483
9.41	-388	9.41	-482
9.42	-389	9.42	-482
9.43	-389	9.43	-482
9.43	-390	9.43	-481
9.44	-390	9.44	-481
9.45	-391	9.45	-480
9.46	-391	9.46	-479
9.47	-391	9.47	-478
9.48	-391	9.48	-478
9.48	-391	9.48	-477
9.49	-391	9.49	-477
9.50	-392	9.50	-476
9.51	-392	9.51	-476
9.52	-392	9.52	-475
9.53	-392	9.53	-474
9.53	-392	9.53	-474
9.54	-392	9.54	-474
9.55	-393	9.55	-473
9.56	-393	9.56	-473
9.57	-393	9.57	-473
9.58	-392	9.58	-473
9.58	-392	9.58	-472
9.59	-392	9.59	-472
9.60	-392	9.60	-472
9.61	-393	9.61	-472

9.62	-393	9.62	-472
9.63	-393	9.63	-471
9.63	-393	9.63	-470
9.64	-393	9.64	-469
9.65	-393	9.65	-469
9.66	-393	9.66	-469
9.67	-393	9.67	-469
9.68	-393	9.68	-469
9.68	-393	9.68	-469
9.69	-393	9.69	-468
9.70	-392	9.70	-468
9.71	-392	9.71	-468
9.72	-392	9.72	-468
9.73	-391	9.73	-468
9.73	-392	9.73	-467
9.74	-392	9.74	-467
9.75	-392	9.75	-467
9.76	-392	9.76	-466
9.77	-391	9.77	-466
9.78	-390	9.78	-465
9.78	-388	9.78	-464
9.79	-388	9.79	-463
9.80	-388	9.80	-463
9.81	-388	9.81	-463
9.82	-387	9.82	-464
9.83	-387	9.83	-464
9.83	-387	9.83	-463
9.84	-386	9.84	-463
9.85	-386	9.85	-463
9.86	-386	9.86	-463
9.87	-386	9.87	-463
9.88	-385	9.88	-463
9.88	-385	9.88	-463
9.89	-384	9.89	-462
9.90	-384	9.90	-462
9.91	-384	9.91	-462
9.92	-383	9.92	-462
9.93	-382	9.93	-462
9.93	-382	9.93	-462
9.94	-381	9.94	-462
9.95	-382	9.95	-462
9.96	-382	9.96	-462
9.97	-381	9.97	-461

9.98	-381	9.98	-461
9.98	-381	9.98	-461
9.99	-381	9.99	-460
10.00	-381	10.00	-460
10.01	-381	10.01	-459
10.02	-381	10.02	-459
10.03	-381	10.03	-459
10.03	-381	10.03	-459
10.04	-380	10.04	-459
10.05	-380	10.05	-459
10.06	-379	10.06	-458
10.07	-379	10.07	-458
10.08	-379	10.08	-458
10.08	-379	10.08	-457
10.09	-379	10.09	-457
10.10	-378	10.10	-456
10.11	-377	10.11	-456
10.12	-377	10.12	-455
10.13	-376	10.13	-454
10.13	-376	10.13	-454
10.14	-375	10.14	-454
10.15	-374	10.15	-454
10.16	-374	10.16	-454
10.17	-373	10.17	-454
10.18	-373	10.18	-454
10.18	-373	10.18	-453
10.19	-372	10.19	-454
10.20	-372	10.20	-454
10.21	-372	10.21	-453
10.22	-371	10.22	-453
10.23	-370	10.23	-453
10.23	-369	10.23	-453
10.24	-367	10.24	-453
10.25	-366	10.25	-452
10.26	-366	10.26	-451
10.27	-366	10.27	-450
10.28	-366	10.28	-450
10.28	-367	10.28	-449
10.29	-367	10.29	-448
10.30	-366	10.30	-448
10.31	-365	10.31	-448
10.32	-365	10.32	-447
10.33	-364	10.33	-447

10.33	-363	10.33	-447
10.34	-363	10.34	-447
10.35	-364	10.35	-447
10.36	-365	10.36	-447
10.37	-365	10.37	-447
10.38	-365	10.38	-446
10.38	-365	10.38	-446
10.39	-365	10.39	-445
10.40	-365	10.40	-445
10.41	-365	10.41	-445
10.42	-364	10.42	-444
10.43	-363	10.43	-444
10.43	-362	10.43	-443
10.44	-361	10.44	-443
10.45	-361	10.45	-443
10.46	-360	10.46	-443
10.47	-360	10.47	-443
10.48	-360	10.48	-442
10.48	-360	10.48	-442
10.49	-359	10.49	-442
10.50	-359	10.50	-442
10.51	-359	10.51	-443
10.52	-359	10.52	-442
10.53	-358	10.53	-442
10.53	-358	10.53	-442
10.54	-357	10.54	-441
10.55	-357	10.55	-441
10.56	-356	10.56	-441
10.57	-355	10.57	-440
10.58	-355	10.58	-440
10.58	-355	10.58	-440
10.59	-355	10.59	-439
10.60	-355	10.60	-439
10.61	-355	10.61	-439
10.62	-354	10.62	-438
10.63	-354	10.63	-438
10.63	-354	10.63	-438
10.64	-354	10.64	-438
10.65	-354	10.65	-438
10.66	-354	10.66	-438
10.67	-353	10.67	-438
10.68	-353	10.68	-438
10.68	-353	10.68	-438

10.69	-353	10.69	-437
10.70	-353	10.70	-436
10.71	-352	10.71	-435
10.72	-352	10.72	-434
10.73	-352	10.73	-434
10.73	-352	10.73	-434
10.74	-352	10.74	-434
10.75	-352	10.75	-435
10.76	-352	10.76	-435
10.77	-352	10.77	-435
10.78	-352	10.78	-435
10.78	-352	10.78	-434
10.79	-351	10.79	-433
10.80	-351	10.80	-433
10.81	-351	10.81	-432
10.82	-350	10.82	-432
10.83	-349	10.83	-431
10.83	-349	10.83	-430
10.84	-348	10.84	-429
10.85	-348	10.85	-429
10.86	-348	10.86	-429
10.87	-348	10.87	-428
10.88	-348	10.88	-428
10.88	-348	10.88	-428
10.89	-349	10.89	-428
10.90	-349	10.90	-428
10.91	-349	10.91	-428
10.92	-349	10.92	-429
10.93	-349	10.93	-429
10.93	-348	10.93	-429
10.94	-348	10.94	-428
10.95	-347	10.95	-427
10.96	-346	10.96	-427
10.97	-346	10.97	-426
10.98	-346	10.98	-425
10.98	-346	10.98	-424
10.99	-346	10.99	-424
11.00	-345	11.00	-424
11.01	-345	11.01	-424
11.02	-344	11.02	-425
11.03	-344	11.03	-424
11.03	-344	11.03	-424
11.04	-343	11.04	-423

11.05	-343	11.05	-423
11.06	-343	11.06	-423
11.07	-343	11.07	-422
11.08	-343	11.08	-421
11.08	-343	11.08	-420
11.09	-343	11.09	-419
11.10	-343	11.10	-419
11.11	-343	11.11	-418
11.12	-342	11.12	-418
11.13	-342	11.13	-418
11.13	-341	11.13	-419
11.14	-341	11.14	-419
11.15	-340	11.15	-419
11.16	-340	11.16	-419
11.17	-339	11.17	-419
11.18	-339	11.18	-419
11.18	-338	11.18	-419
11.19	-339	11.19	-419
11.20	-339	11.20	-419
11.21	-339	11.21	-420
11.22	-339	11.22	-419
11.23	-338	11.23	-419
11.23	-337	11.23	-418
11.24	-336	11.24	-418
11.25	-335	11.25	-417
11.26	-335	11.26	-416
11.27	-335	11.27	-416
11.28	-335	11.28	-416
11.28	-335	11.28	-415
11.29	-335	11.29	-415
11.30	-336	11.30	-415
11.31	-336	11.31	-415
11.32	-337	11.32	-415
11.33	-337	11.33	-415
11.33	-337	11.33	-415
11.34	-336	11.34	-415
11.35	-336	11.35	-414
11.36	-337	11.36	-414
11.37	-337	11.37	-414
11.38	-336	11.38	-414
11.38	-335	11.38	-414
11.39	-335	11.39	-414
11.40	-334	11.40	-413

11.41	-334	11.41	-413
11.42	-333	11.42	-413
11.43	-333	11.43	-413
11.43	-333	11.43	-412
11.44	-333	11.44	-411
11.45	-333	11.45	-410
11.46	-334	11.46	-410
11.47	-334	11.47	-410
11.48	-335	11.48	-410
11.48	-334	11.48	-410
11.49	-333	11.49	-411
11.50	-333	11.50	-410
11.51	-332	11.51	-410
11.52	-332	11.52	-410
11.53	-331	11.53	-410
11.53	-330	11.53	-409
11.54	-329	11.54	-408
11.55	-328	11.55	-408
11.56	-328	11.56	-408
11.57	-327	11.57	-408
11.58	-326	11.58	-408
11.58	-325	11.58	-408
11.59	-325	11.59	-409
11.60	-324	11.60	-408
11.61	-324	11.61	-407
11.62	-324	11.62	-407
11.63	-323	11.63	-406
11.63	-323	11.63	-406
11.64	-323	11.64	-406
11.65	-323	11.65	-405
11.66	-323	11.66	-405
11.67	-322	11.67	-405
11.68	-322	11.68	-404
11.68	-321	11.68	-404
11.69	-320	11.69	-404
11.70	-320	11.70	-404
11.71	-320	11.71	-404
11.72	-320	11.72	-404
11.73	-319	11.73	-404
11.73	-319	11.73	-403
11.74	-318	11.74	-403
11.75	-318	11.75	-403
11.76	-317	11.76	-402

11.77	-317	11.77	-402
11.78	-317	11.78	-402
11.78	-317	11.78	-402
11.79	-317	11.79	-401
11.80	-317	11.80	-401
11.81	-317	11.81	-401
11.82	-317	11.82	-400
11.83	-317	11.83	-400
11.83	-317	11.83	-400
11.84	-316	11.84	-400
11.85	-316	11.85	-401
11.86	-316	11.86	-400
11.87	-315	11.87	-400
11.88	-315	11.88	-399
11.88	-314	11.88	-399
11.89	-313	11.89	-399
11.90	-312	11.90	-398
11.91	-311	11.91	-398
11.92	-310	11.92	-398
11.93	-309	11.93	-398
11.93	-309	11.93	-397
11.94	-308	11.94	-396
11.95	-308	11.95	-395
11.96	-307	11.96	-395
11.97	-306	11.97	-395
11.98	-306	11.98	-395
11.98	-305	11.98	-394
11.99	-304	11.99	-394
12.00	-304	12.00	-394
12.01	-305	12.01	-394
12.02	-305	12.02	-394
12.03	-305	12.03	-394
12.03	-304	12.03	-394
12.04	-304	12.04	-394
12.05	-305	12.05	-393
12.06	-305	12.06	-393
12.07	-305	12.07	-393
12.08	-304	12.08	-392
12.08	-304	12.08	-391
12.09	-304	12.09	-390
12.10	-305	12.10	-390
12.11	-304	12.11	-389
12.12	-304	12.12	-389

12.13	-305	12.13	-388
12.13	-305	12.13	-389
12.14	-304	12.14	-388
12.15	-304	12.15	-388
12.16	-304	12.16	-387
12.17	-304	12.17	-386
12.18	-303	12.18	-385
12.18	-302	12.18	-385
12.19	-302	12.19	-385
12.20	-302	12.20	-384
12.21	-302	12.21	-384
12.22	-303	12.22	-384
12.23	-303	12.23	-384
12.23	-303	12.23	-383
12.24	-302	12.24	-383
12.25	-301	12.25	-383
12.26	-300	12.26	-384
12.27	-300	12.27	-385
12.28	-299	12.28	-384
12.28	-298	12.28	-383
12.29	-298	12.29	-383
12.30	-297	12.30	-383
12.31	-297	12.31	-383
12.32	-297	12.32	-383
12.33	-296	12.33	-383
12.33	-296	12.33	-383
12.34	-295	12.34	-383
12.35	-293	12.35	-383
12.36	-292	12.36	-382
12.37	-291	12.37	-382
12.38	-291	12.38	-382
12.38	-291	12.38	-382
12.39	-290	12.39	-381
12.40	-290	12.40	-380
12.41	-289	12.41	-379
12.42	-289	12.42	-379
12.43	-289	12.43	-379
12.43	-289	12.43	-380
12.44	-288	12.44	-380
12.45	-287	12.45	-380
12.46	-287	12.46	-380
12.47	-286	12.47	-380
12.48	-285	12.48	-381

12.48	-284	12.48	-381
12.49	-284	12.49	-381
12.50	-283	12.50	-381
12.51	-283	12.51	-381
12.52	-282	12.52	-380
12.53	-282	12.53	-379
12.53	-282	12.53	-378
12.54	-282	12.54	-377
12.55	-282	12.55	-376
12.56	-282	12.56	-375
12.57	-282	12.57	-374
12.58	-282	12.58	-373
12.58	-282	12.58	-373
12.59	-281	12.59	-373
12.60	-280	12.60	-373
12.61	-279	12.61	-373
12.62	-278	12.62	-372
12.63	-278	12.63	-372
12.63	-278	12.63	-372
12.64	-277	12.64	-372
12.65	-278	12.65	-373
12.66	-278	12.66	-373
12.67	-277	12.67	-373
12.68	-276	12.68	-374
12.68	-276	12.68	-374
12.69	-274	12.69	-373
12.70	-273	12.70	-373
12.71	-272	12.71	-373
12.72	-272	12.72	-373
12.73	-272	12.73	-372
12.73	-272	12.73	-371
12.74	-272	12.74	-371
12.75	-273	12.75	-372
12.76	-273	12.76	-372
12.77	-273	12.77	-373
12.78	-272	12.78	-373
12.78	-272	12.78	-372
12.79	-270	12.79	-372
12.80	-270	12.80	-371
12.81	-269	12.81	-371
12.82	-268	12.82	-370
12.83	-267	12.83	-370
12.83	-266	12.83	-369

12.84	-265	12.84	-368
12.85	-265	12.85	-368
12.86	-265	12.86	-368
12.87	-265	12.87	-369
12.88	-264	12.88	-369
12.88	-264	12.88	-369
12.89	-263	12.89	-368
12.90	-263	12.90	-368
12.91	-263	12.91	-367
12.92	-262	12.92	-367
12.93	-262	12.93	-366
12.93	-261	12.93	-365
12.94	-260	12.94	-365
12.95	-260	12.95	-364
12.96	-259	12.96	-364
12.97	-259	12.97	-364
12.98	-258	12.98	-364
12.98	-258	12.98	-363
12.99	-258	12.99	-362
13.00	-257	13.00	-361
13.01	-256	13.01	-361
13.02	-255	13.02	-361
13.03	-253	13.03	-361
13.03	-252	13.03	-361
13.04	-251	13.04	-360
13.05	-250	13.05	-359
13.06	-250	13.06	-359
13.07	-249	13.07	-358
13.08	-248	13.08	-357
13.08	-248	13.08	-357
13.09	-248	13.09	-357
13.10	-248	13.10	-356
13.11	-247	13.11	-356
13.12	-246	13.12	-356
13.13	-245	13.13	-356
13.13	-244	13.13	-357
13.14	-243	13.14	-357
13.15	-243	13.15	-357
13.16	-244	13.16	-357
13.17	-243	13.17	-356
13.18	-243	13.18	-356
13.18	-243	13.18	-356
13.19	-242	13.19	-355

13.20	-241	13.20	-355
13.21	-241	13.21	-354
13.22	-241	13.22	-354
13.23	-242	13.23	-354
13.23	-242	13.23	-354
13.24	-242	13.24	-354
13.25	-242	13.25	-354
13.26	-242	13.26	-354
13.27	-243	13.27	-354
13.28	-242	13.28	-354
13.28	-242	13.28	-353
13.29	-242	13.29	-352
13.30	-241	13.30	-351
13.31	-242	13.31	-350
13.32	-242	13.32	-350
13.33	-243	13.33	-350
13.33	-243	13.33	-350
13.34	-243	13.34	-350
13.35	-243	13.35	-350
13.36	-244	13.36	-350
13.37	-244	13.37	-350
13.38	-245	13.38	-350
13.38	-246	13.38	-350
13.39	-247	13.39	-350
13.40	-248	13.40	-350
13.41	-248	13.41	-350
13.42	-249	13.42	-351
13.43	-249	13.43	-350
13.43	-249	13.43	-350
13.44	-249	13.44	-350
13.45	-249	13.45	-349
13.46	-249	13.46	-348
13.47	-249	13.47	-348
13.48	-249	13.48	-347
13.48	-250	13.48	-347
13.49	-251	13.49	-346
13.50	-251	13.50	-346
13.51	-251	13.51	-346
13.52	-250	13.52	-346
13.53	-250	13.53	-346
13.53	-249	13.53	-346
13.54	-249	13.54	-345
13.55	-248	13.55	-344

13.56	-248	13.56	-343
13.57	-247	13.57	-343
13.58	-247	13.58	-343
13.58	-247	13.58	-344
13.59	-247	13.59	-345
13.60	-247	13.60	-345
13.61	-247	13.61	-344
13.62	-247	13.62	-344
13.63	-247	13.63	-343
13.63	-248	13.63	-342
13.64	-248	13.64	-342
13.65	-249	13.65	-341
13.66	-249	13.66	-341
13.67	-249	13.67	-340
13.68	-248	13.68	-340
13.68	-248	13.68	-338
13.69	-247	13.69	-338
13.70	-247	13.70	-337
13.71	-247	13.71	-337
13.72	-246	13.72	-337
13.73	-246	13.73	-336
13.73	-245	13.73	-336
13.74	-244	13.74	-336
13.75	-244	13.75	-335
13.76	-244	13.76	-335
13.77	-243	13.77	-335
13.78	-243	13.78	-335
13.78	-242	13.78	-335
13.79	-242	13.79	-334
13.80	-242	13.80	-334
13.81	-241	13.81	-333
13.82	-241	13.82	-333
13.83	-241	13.83	-333
13.83	-241	13.83	-333
13.84	-241	13.84	-334
13.85	-240	13.85	-333
13.86	-240	13.86	-332
13.87	-239	13.87	-332
13.88	-239	13.88	-331
13.88	-239	13.88	-330
13.89	-239	13.89	-329
13.90	-240	13.90	-329
13.91	-240	13.91	-328

13.92	-241	13.92	-328
13.93	-241	13.93	-327
13.93	-241	13.93	-327
13.94	-240	13.94	-327
13.95	-241	13.95	-327
13.96	-241	13.96	-327
13.97	-241	13.97	-327
13.98	-241	13.98	-328
13.98	-241	13.98	-327
13.99	-241	13.99	-327
14.00	-242	14.00	-326
14.01	-242	14.01	-325
14.02	-243	14.02	-325
14.03	-243	14.03	-325
14.03	-243	14.03	-325
14.04	-244	14.04	-324
14.05	-243	14.05	-324
14.06	-243	14.06	-324
14.07	-244	14.07	-324
14.08	-244	14.08	-324
14.08	-243	14.08	-324
14.09	-243	14.09	-324
14.10	-243	14.10	-324
14.11	-243	14.11	-324
14.12	-242	14.12	-323
14.13	-242	14.13	-322
14.13	-241	14.13	-322
14.14	-241	14.14	-321
14.15	-242	14.15	-321
14.16	-242	14.16	-322
14.17	-242	14.17	-322
14.18	-242	14.18	-322
14.18	-241	14.18	-321
14.19	-240	14.19	-321
14.20	-239	14.20	-320
14.21	-239	14.21	-320
14.22	-238	14.22	-319
14.23	-238	14.23	-319
14.23	-238	14.23	-319
14.24	-238	14.24	-318
14.25	-238	14.25	-317
14.26	-238	14.26	-316
14.27	-238	14.27	-315

14.28	-238	14.28	-314
14.28	-237	14.28	-313
14.29	-237	14.29	-313
14.30	-236	14.30	-314
14.31	-236	14.31	-314
14.32	-236	14.32	-313
14.33	-236	14.33	-313
14.33	-236	14.33	-313
14.34	-235	14.34	-312
14.35	-234	14.35	-312
14.36	-233	14.36	-312
14.37	-231	14.37	-312
14.38	-230	14.38	-312
14.38	-229	14.38	-312
14.39	-228	14.39	-312
14.40	-227	14.40	-312
14.41	-226	14.41	-312
14.42	-225	14.42	-312
14.43	-225	14.43	-312
14.43	-225	14.43	-312
14.44	-225	14.44	-311
14.45	-225	14.45	-310
14.46	-225	14.46	-310
14.47	-225	14.47	-310
14.48	-224	14.48	-309
14.48	-224	14.48	-309
14.49	-223	14.49	-309
14.50	-222	14.50	-309
14.51	-221	14.51	-310
14.52	-220	14.52	-310
14.53	-219	14.53	-310
14.53	-219	14.53	-310
14.54	-220	14.54	-310
14.55	-220	14.55	-309
14.56	-220	14.56	-309
14.57	-220	14.57	-308
14.58	-219	14.58	-308
14.58	-218	14.58	-307
14.59	-217	14.59	-307
14.60	-217	14.60	-307
14.61	-217	14.61	-306
14.62	-217	14.62	-306
14.63	-216	14.63	-305

14.63	-216	14.63	-304
14.64	-216	14.64	-304
14.65	-216	14.65	-303
14.66	-216	14.66	-303
14.67	-216	14.67	-301
14.68	-216	14.68	-300
14.68	-216	14.68	-300
14.69	-215	14.69	-299
14.70	-215	14.70	-299
14.71	-215	14.71	-299
14.72	-214	14.72	-300
14.73	-214	14.73	-299
14.73	-213	14.73	-300
14.74	-213	14.74	-300
14.75	-212	14.75	-300
14.76	-212	14.76	-300
14.77	-212	14.77	-300
14.78	-213	14.78	-300
14.78	-213	14.78	-300
14.79	-213	14.79	-300
14.80	-212	14.80	-300
14.81	-213	14.81	-300
14.82	-213	14.82	-299
14.83	-213	14.83	-299
14.83	-212	14.83	-298
14.84	-213	14.84	-298
14.85	-213	14.85	-297
14.86	-212	14.86	-297
14.87	-211	14.87	-297
14.88	-211	14.88	-296
14.88	-210	14.88	-295
14.89	-210	14.89	-295
14.90	-210	14.90	-295
14.91	-209	14.91	-294
14.92	-209	14.92	-294
14.93	-209	14.93	-294
14.93	-209	14.93	-293
14.94	-209	14.94	-292
14.95	-209	14.95	-292
14.96	-209	14.96	-293
14.97	-208	14.97	-293
14.98	-208	14.98	-293
14.98	-207	14.98	-293

14.99	-206	14.99	-293
15.00	-206	15.00	-294
15.01	-206	15.01	-293
15.02	-206	15.02	-292
15.03	-206	15.03	-292
15.03	-206	15.03	-292
15.04	-205	15.04	-291
15.05	-205	15.05	-290
15.06	-204	15.06	-290
15.07	-204	15.07	-290
15.08	-203	15.08	-290
15.08	-203	15.08	-290
15.09	-202	15.09	-289
15.10	-202	15.10	-289
15.11	-202	15.11	-289
15.12	-203	15.12	-288
15.13	-203	15.13	-289
15.13	-203	15.13	-289
15.14	-202	15.14	-289
15.15	-202	15.15	-289
15.16	-202	15.16	-290
15.17	-202	15.17	-289
15.18	-201	15.18	-289
15.18	-201	15.18	-288
15.19	-201	15.19	-287
15.20	-201	15.20	-286
15.21	-200	15.21	-285
15.22	-200	15.22	-285
15.23	-199	15.23	-285
15.23	-199	15.23	-285
15.24	-198	15.24	-285
15.25	-198	15.25	-285
15.26	-198	15.26	-285
15.27	-198	15.27	-285
15.28	-198	15.28	-284
15.28	-198	15.28	-283
15.29	-199	15.29	-283
15.30	-199	15.30	-283
15.31	-200	15.31	-283
15.32	-200	15.32	-283
15.33	-199	15.33	-283
15.33	-199	15.33	-282
15.34	-200	15.34	-281

15.35	-200	15.35	-280
15.36	-200	15.36	-279
15.37	-200	15.37	-279
15.38	-200	15.38	-278
15.38	-200	15.38	-277
15.39	-200	15.39	-277
15.40	-199	15.40	-277
15.41	-199	15.41	-277
15.42	-198	15.42	-277
15.43	-198	15.43	-277
15.43	-198	15.43	-277
15.44	-199	15.44	-277
15.45	-199	15.45	-276
15.46	-200	15.46	-276
15.47	-200	15.47	-276
15.48	-199	15.48	-276
15.48	-200	15.48	-275
15.49	-200	15.49	-275
15.50	-199	15.50	-275
15.51	-199	15.51	-275
15.52	-199	15.52	-275
15.53	-198	15.53	-275
15.53	-198	15.53	-275
15.54	-198	15.54	-275
15.55	-198	15.55	-275
15.56	-197	15.56	-274
15.57	-197	15.57	-274
15.58	-197	15.58	-274
15.58	-197	15.58	-273
15.59	-197	15.59	-273
15.60	-198	15.60	-272
15.61	-198	15.61	-272
15.62	-198	15.62	-271
15.63	-198	15.63	-271
15.63	-197	15.63	-271
15.64	-196	15.64	-270
15.65	-195	15.65	-269
15.66	-195	15.66	-268
15.67	-195	15.67	-268
15.68	-195	15.68	-267
15.68	-194	15.68	-267
15.69	-194	15.69	-266
15.70	-193	15.70	-266

15.71	-193	15.71	-265
15.72	-192	15.72	-265
15.73	-191	15.73	-265
15.73	-191	15.73	-264
15.74	-190	15.74	-264
15.75	-189	15.75	-264
15.76	-189	15.76	-264
15.77	-189	15.77	-264
15.78	-189	15.78	-264
15.78	-189	15.78	-265
15.79	-189	15.79	-264
15.80	-189	15.80	-264
15.81	-189	15.81	-263
15.82	-188	15.82	-262
15.83	-188	15.83	-261
15.83	-188	15.83	-261
15.84	-188	15.84	-260
15.85	-187	15.85	-260
15.86	-187	15.86	-260
15.87	-187	15.87	-260
15.88	-186	15.88	-259
15.88	-186	15.88	-259
15.89	-185	15.89	-259
15.90	-185	15.90	-259
15.91	-185	15.91	-259
15.92	-185	15.92	-258
15.93	-185	15.93	-258
15.93	-185	15.93	-258
15.94	-185	15.94	-257
15.95	-184	15.95	-257
15.96	-183	15.96	-257
15.97	-183	15.97	-257
15.98	-183	15.98	-256
15.98	-183	15.98	-256
15.99	-183	15.99	-255
16.00	-183	16.00	-255
16.01	-183	16.01	-255
16.02	-183	16.02	-256
16.03	-183	16.03	-256
16.03	-183	16.03	-257
16.04	-183	16.04	-257
16.05	-183	16.05	-258
16.06	-183	16.06	-258

16.07	-183	16.07	-258
16.08	-183	16.08	-258
16.08	-183	16.08	-257
16.09	-182	16.09	-256
16.10	-182	16.10	-255
16.11	-182	16.11	-255
16.12	-182	16.12	-255
16.13	-182	16.13	-255
16.13	-182	16.13	-254
16.14	-181	16.14	-254
16.15	-181	16.15	-254
16.16	-181	16.16	-253
16.17	-181	16.17	-253
16.18	-181	16.18	-252
16.18	-182	16.18	-252
16.19	-181	16.19	-251
16.20	-180	16.20	-251
16.21	-179	16.21	-251
16.22	-179	16.22	-250
16.23	-178	16.23	-250
16.23	-177	16.23	-250
16.24	-176	16.24	-250
16.25	-175	16.25	-250
16.26	-175	16.26	-249
16.27	-175	16.27	-248
16.28	-175	16.28	-248
16.28	-175	16.28	-247
16.29	-175	16.29	-247
16.30	-175	16.30	-247
16.31	-174	16.31	-247
16.32	-175	16.32	-247
16.33	-175	16.33	-248
16.33	-174	16.33	-248
16.34	-173	16.34	-248
16.35	-173	16.35	-247
16.36	-173	16.36	-247
16.37	-172	16.37	-247
16.38	-171	16.38	-247
16.38	-170	16.38	-247
16.39	-171	16.39	-246
16.40	-170	16.40	-245
16.41	-170	16.41	-245
16.42	-170	16.42	-245

16.43	-170	16.43	-245
16.43	-170	16.43	-244
16.44	-169	16.44	-243
16.45	-169	16.45	-241
16.46	-168	16.46	-241
16.47	-168	16.47	-240
16.48	-167	16.48	-239
16.48	-167	16.48	-239
16.49	-167	16.49	-239
16.50	-167	16.50	-240
16.51	-167	16.51	-240
16.52	-166	16.52	-240
16.53	-166	16.53	-240
16.53	-165	16.53	-240
16.54	-166	16.54	-240
16.55	-166	16.55	-239
16.56	-166	16.56	-239
16.57	-166	16.57	-239
16.58	-166	16.58	-239
16.58	-166	16.58	-239
16.59	-166	16.59	-239
16.60	-165	16.60	-239
16.61	-164	16.61	-239
16.62	-164	16.62	-238
16.63	-164	16.63	-238
16.63	-163	16.63	-237
16.64	-163	16.64	-237
16.65	-162	16.65	-236
16.66	-162	16.66	-236
16.67	-162	16.67	-235
16.68	-162	16.68	-235
16.68	-162	16.68	-235
16.69	-163	16.69	-234
16.70	-163	16.70	-234
16.71	-163	16.71	-234
16.72	-163	16.72	-234
16.73	-162	16.73	-234
16.73	-162	16.73	-234
16.74	-162	16.74	-234
16.75	-162	16.75	-234
16.76	-163	16.76	-234
16.77	-163	16.77	-233
16.78	-162	16.78	-233

16.78	-161	16.78	-232
16.79	-160	16.79	-232
16.80	-159	16.80	-232
16.81	-158	16.81	-231
16.82	-156	16.82	-231
16.83	-156	16.83	-231
16.83	-156	16.83	-232
16.84	-155	16.84	-232
16.85	-155	16.85	-232
16.86	-155	16.86	-232
16.87	-155	16.87	-232
16.88	-155	16.88	-232
16.88	-155	16.88	-232
16.89	-155	16.89	-233
16.90	-155	16.90	-232
16.91	-154	16.91	-233
16.92	-154	16.92	-233
16.93	-154	16.93	-233
16.93	-154	16.93	-233
16.94	-154	16.94	-232
16.95	-153	16.95	-232
16.96	-153	16.96	-231
16.97	-153	16.97	-230
16.98	-153	16.98	-229
16.98	-153	16.98	-228
16.99	-153	16.99	-227
17.00	-153	17.00	-227
17.01	-152	17.01	-227
17.02	-152	17.02	-227
17.03	-151	17.03	-227
17.03	-151	17.03	-227
17.04	-151	17.04	-227
17.05	-151	17.05	-226
17.06	-151	17.06	-226
17.07	-151	17.07	-225
17.08	-150	17.08	-225
17.08	-151	17.08	-224
17.09	-150	17.09	-224
17.10	-150	17.10	-224
17.11	-150	17.11	-223
17.12	-148	17.12	-223
17.13	-147	17.13	-222
17.13	-146	17.13	-222

17.14	-146	17.14	-222
17.15	-146	17.15	-222
17.16	-146	17.16	-222
17.17	-146	17.17	-222
17.18	-146	17.18	-221
17.18	-146	17.18	-221
17.19	-146	17.19	-220
17.20	-146	17.20	-220
17.21	-146	17.21	-220
17.22	-146	17.22	-220
17.23	-145	17.23	-219
17.23	-146	17.23	-220
17.24	-146	17.24	-219
17.25	-145	17.25	-219
17.26	-145	17.26	-218
17.27	-145	17.27	-218
17.28	-145	17.28	-217
17.28	-144	17.28	-217
17.29	-144	17.29	-217
17.30	-143	17.30	-218
17.31	-143	17.31	-218
17.32	-143	17.32	-218
17.33	-142	17.33	-218
17.33	-142	17.33	-218
17.34	-142	17.34	-218
17.35	-143	17.35	-218
17.36	-143	17.36	-218
17.37	-143	17.37	-218
17.38	-143	17.38	-218
17.38	-142	17.38	-218
17.39	-142	17.39	-217
17.40	-142	17.40	-217
17.41	-142	17.41	-216
17.42	-141	17.42	-215
17.43	-140	17.43	-215
17.43	-140	17.43	-214
17.44	-139	17.44	-214
17.45	-139	17.45	-213
17.46	-139	17.46	-212
17.47	-138	17.47	-212
17.48	-138	17.48	-212
17.48	-138	17.48	-212
17.49	-137	17.49	-212

17.50	-136	17.50	-211
17.51	-136	17.51	-211
17.52	-135	17.52	-211
17.53	-135	17.53	-210
17.53	-135	17.53	-209
17.54	-136	17.54	-209
17.55	-137	17.55	-208
17.56	-137	17.56	-208
17.57	-137	17.57	-208
17.58	-136	17.58	-208
17.58	-136	17.58	-208
17.59	-135	17.59	-208
17.60	-135	17.60	-208
17.61	-134	17.61	-207
17.62	-133	17.62	-207
17.63	-132	17.63	-207
17.63	-132	17.63	-207
17.64	-131	17.64	-207
17.65	-130	17.65	-208
17.66	-130	17.66	-208
17.67	-131	17.67	-207
17.68	-130	17.68	-207
17.68	-129	17.68	-207
17.69	-129	17.69	-206
17.70	-129	17.70	-206
17.71	-128	17.71	-205
17.72	-128	17.72	-205
17.73	-128	17.73	-204
17.73	-128	17.73	-204
17.74	-128	17.74	-203
17.75	-128	17.75	-203
17.76	-129	17.76	-203
17.77	-129	17.77	-203
17.78	-129	17.78	-203
17.78	-129	17.78	-202
17.79	-128	17.79	-202
17.80	-128	17.80	-202
17.81	-127	17.81	-201
17.82	-127	17.82	-201
17.83	-126	17.83	-201
17.83	-126	17.83	-200
17.84	-125	17.84	-200
17.85	-124	17.85	-200

17.86	-124	17.86	-199
17.87	-123	17.87	-198
17.88	-123	17.88	-197
17.88	-123	17.88	-197
17.89	-124	17.89	-196
17.90	-124	17.90	-196
17.91	-124	17.91	-195
17.92	-123	17.92	-194
17.93	-123	17.93	-193
17.93	-123	17.93	-193
17.94	-123	17.94	-192
17.95	-123	17.95	-191
17.96	-122	17.96	-191
17.97	-122	17.97	-191
17.98	-122	17.98	-190
17.98	-121	17.98	-190
17.99	-121	17.99	-190
18.00	-120	18.00	-189
18.01	-120	18.01	-189
18.02	-119	18.02	-188
18.03	-119	18.03	-188
18.03	-119	18.03	-188
18.04	-120	18.04	-188
18.05	-120	18.05	-188
18.06	-120	18.06	-189
18.07	-119	18.07	-190
18.08	-118	18.08	-189
18.08	-118	18.08	-188
18.09	-117	18.09	-188
18.10	-117	18.10	-188
18.11	-116	18.11	-187
18.12	-116	18.12	-187
18.13	-116	18.13	-187
18.13	-116	18.13	-187
18.14	-116	18.14	-187
18.15	-116	18.15	-186
18.16	-116	18.16	-185
18.17	-116	18.17	-185
18.18	-116	18.18	-185
18.18	-116	18.18	-185
18.19	-115	18.19	-185
18.20	-115	18.20	-184
18.21	-114	18.21	-183

18.22	-114	18.22	-182
18.23	-114	18.23	-181
18.23	-113	18.23	-181
18.24	-113	18.24	-181
18.25	-112	18.25	-181
18.26	-111	18.26	-181
18.27	-110	18.27	-181
18.28	-110	18.28	-182
18.28	-110	18.28	-181
18.29	-111	18.29	-181
18.30	-111	18.30	-181
18.31	-111	18.31	-180
18.32	-111	18.32	-180
18.33	-110	18.33	-180
18.33	-109	18.33	-179
18.34	-109	18.34	-179
18.35	-109	18.35	-178
18.36	-109	18.36	-177
18.37	-109	18.37	-176
18.38	-108	18.38	-175
18.38	-107	18.38	-175
18.39	-106	18.39	-174
18.40	-106	18.40	-174
18.41	-105	18.41	-174
18.42	-105	18.42	-174
18.43	-104	18.43	-174
18.43	-104	18.43	-173
18.44	-104	18.44	-173
18.45	-104	18.45	-174
18.46	-104	18.46	-174
18.47	-104	18.47	-174
18.48	-104	18.48	-173
18.48	-104	18.48	-173
18.49	-104	18.49	-172
18.50	-104	18.50	-172
18.51	-105	18.51	-171
18.52	-105	18.52	-171
18.53	-105	18.53	-171
18.53	-105	18.53	-171
18.54	-104	18.54	-171
18.55	-104	18.55	-170
18.56	-103	18.56	-170
18.57	-104	18.57	-170

18.58	-104	18.58	-170
18.58	-104	18.58	-170
18.59	-103	18.59	-170
18.60	-103	18.60	-170
18.61	-102	18.61	-170
18.62	-103	18.62	-169
18.63	-103	18.63	-169
18.63	-103	18.63	-169
18.64	-103	18.64	-168
18.65	-103	18.65	-168
18.66	-103	18.66	-168
18.67	-102	18.67	-167
18.68	-101	18.68	-166
18.68	-100	18.68	-166
18.69	-99	18.69	-166
18.70	-98	18.70	-165
18.71	-98	18.71	-165
18.72	-98	18.72	-165
18.73	-99	18.73	-165
18.73	-99	18.73	-164
18.74	-99	18.74	-164
18.75	-99	18.75	-164
18.76	-98	18.76	-163
18.77	-97	18.77	-162
18.78	-96	18.78	-162
18.78	-95	18.78	-161
18.79	-95	18.79	-161
18.80	-94	18.80	-161
18.81	-94	18.81	-161
18.82	-94	18.82	-160
18.83	-94	18.83	-160
18.83	-94	18.83	-161
18.84	-94	18.84	-160
18.85	-94	18.85	-160
18.86	-94	18.86	-159
18.87	-95	18.87	-159
18.88	-94	18.88	-159
18.88	-94	18.88	-159
18.89	-93	18.89	-159
18.90	-92	18.90	-158
18.91	-92	18.91	-158
18.92	-92	18.92	-157
18.93	-92	18.93	-157

18.93	-92	18.93	-157
18.94	-92	18.94	-157
18.95	-91	18.95	-157
18.96	-91	18.96	-157
18.97	-90	18.97	-156
18.98	-90	18.98	-155
18.98	-90	18.98	-155
18.99	-90	18.99	-154
19.00	-89	19.00	-153
19.01	-88	19.01	-153
19.02	-88	19.02	-153
19.03	-88	19.03	-153
19.03	-87	19.03	-153
19.04	-86	19.04	-153
19.05	-85	19.05	-154
19.06	-85	19.06	-154
19.07	-85	19.07	-154
19.08	-85	19.08	-154
19.08	-85	19.08	-154
19.09	-85	19.09	-153
19.10	-85	19.10	-153
19.11	-85	19.11	-152
19.12	-85	19.12	-151
19.13	-85	19.13	-151
19.13	-85	19.13	-151
19.14	-84	19.14	-151
19.15	-84	19.15	-151
19.16	-84	19.16	-151
19.17	-83	19.17	-151
19.18	-83	19.18	-150
19.18	-83	19.18	-150
19.19	-83	19.19	-149
19.20	-82	19.20	-148
19.21	-82	19.21	-148
19.22	-81	19.22	-147
19.23	-80	19.23	-147
19.23	-79	19.23	-146
19.24	-78	19.24	-146
19.25	-78	19.25	-145
19.26	-78	19.26	-144
19.27	-78	19.27	-144
19.28	-77	19.28	-144
19.28	-77	19.28	-144

19.29	-77	19.29	-144
19.30	-77	19.30	-144
19.31	-77	19.31	-144
19.32	-78	19.32	-145
19.33	-79	19.33	-144
19.33	-78	19.33	-144
19.34	-78	19.34	-143
19.35	-78	19.35	-143
19.36	-77	19.36	-143
19.37	-77	19.37	-142
19.38	-77	19.38	-142
19.38	-76	19.38	-142
19.39	-76	19.39	-141
19.40	-75	19.40	-141
19.41	-74	19.41	-141
19.42	-73	19.42	-141
19.43	-72	19.43	-141
19.43	-72	19.43	-141
19.44	-72	19.44	-141
19.45	-72	19.45	-141
19.46	-71	19.46	-141
19.47	-71	19.47	-141
19.48	-71	19.48	-141
19.48	-71	19.48	-141
19.49	-72	19.49	-141
19.50	-72	19.50	-141
19.51	-73	19.51	-140
19.52	-73	19.52	-139
19.53	-72	19.53	-138
19.53	-71	19.53	-138
19.54	-71	19.54	-138
19.55	-70	19.55	-137
19.56	-70	19.56	-136
19.57	-70	19.57	-135
19.58	-70	19.58	-134
19.58	-71	19.58	-133
19.59	-71	19.59	-133
19.60	-71	19.60	-134
19.61	-70	19.61	-133
19.62	-70	19.62	-133
19.63	-69	19.63	-133
19.63	-69	19.63	-132
19.64	-69	19.64	-132

19.65	-69	19.65	-131
19.66	-68	19.66	-131
19.67	-68	19.67	-130
19.68	-67	19.68	-130
19.68	-67	19.68	-130
19.69	-67	19.69	-131
19.70	-67	19.70	-131
19.71	-67	19.71	-130
19.72	-67	19.72	-130
19.73	-67	19.73	-129
19.73	-67	19.73	-129
19.74	-67	19.74	-128
19.75	-66	19.75	-128
19.76	-66	19.76	-127
19.77	-65	19.77	-127
19.78	-65	19.78	-127
19.78	-65	19.78	-127
19.79	-64	19.79	-127
19.80	-64	19.80	-127
19.81	-64	19.81	-127
19.82	-64	19.82	-126
19.83	-64	19.83	-126
19.83	-64	19.83	-125
19.84	-63	19.84	-125
19.85	-63	19.85	-125
19.86	-63	19.86	-125
19.87	-64	19.87	-125
19.88	-64	19.88	-125
19.88	-64	19.88	-125
19.89	-64	19.89	-125
19.90	-63	19.90	-124
19.91	-63	19.91	-124
19.92	-63	19.92	-123
19.93	-63	19.93	-123
19.93	-64	19.93	-123
19.94	-64	19.94	-123
19.95	-64	19.95	-123
19.96	-63	19.96	-122
19.97	-63	19.97	-122
19.98	-62	19.98	-122
19.98	-62	19.98	-122
19.99	-62	19.99	-121
20.00	-62	20.00	-121

20.01	-61	20.01	-121
20.02	-61	20.02	-120
20.03	-61	20.03	-119
20.03	-61	20.03	-119
20.04	-60	20.04	-118
20.05	-59	20.05	-118
20.06	-59	20.06	-118
20.07	-59	20.07	-117
20.08	-59	20.08	-117
20.08	-59	20.08	-116
20.09	-59	20.09	-116
20.10	-58	20.10	-116
20.11	-57	20.11	-115
20.12	-56	20.12	-116
20.13	-56	20.13	-116
20.13	-55	20.13	-116
20.14	-54	20.14	-116
20.15	-53	20.15	-116
20.16	-52	20.16	-117
20.17	-52	20.17	-116
20.18	-52	20.18	-116
20.18	-52	20.18	-114
20.19	-52	20.19	-113
20.20	-51	20.20	-112
20.21	-52	20.21	-112
20.22	-52	20.22	-111
20.23	-52	20.23	-111
20.23	-52	20.23	-111
20.24	-53	20.24	-111
20.25	-53	20.25	-111
20.26	-53	20.26	-110
20.27	-52	20.27	-109
20.28	-52	20.28	-108
20.28	-51	20.28	-108
20.29	-51	20.29	-109
20.30	-50	20.30	-109
20.31	-49	20.31	-109
20.32	-48	20.32	-109
20.33	-47	20.33	-108
20.33	-47	20.33	-108
20.34	-47	20.34	-107
20.35	-47	20.35	-106
20.36	-47	20.36	-105

20.37	-47	20.37	-105
20.38	-47	20.38	-104
20.38	-46	20.38	-104
20.39	-46	20.39	-104
20.40	-46	20.40	-103
20.41	-46	20.41	-103
20.42	-45	20.42	-103
20.43	-44	20.43	-103
20.43	-44	20.43	-102
20.44	-43	20.44	-101
20.45	-43	20.45	-101
20.46	-43	20.46	-101
20.47	-43	20.47	-101
20.48	-43	20.48	-102
20.48	-42	20.48	-102
20.49	-42	20.49	-101
20.50	-41	20.50	-101
20.51	-41	20.51	-100
20.52	-40	20.52	-99
20.53	-41	20.53	-99
20.53	-40	20.53	-99
20.54	-40	20.54	-99
20.55	-40	20.55	-99
20.56	-40	20.56	-98
20.57	-40	20.57	-97
20.58	-39	20.58	-97
20.58	-39	20.58	-97
20.59	-38	20.59	-97
20.60	-38	20.60	-96
20.61	-37	20.61	-96
20.62	-36	20.62	-95
20.63	-35	20.63	-95
20.63	-35	20.63	-95
20.64	-34	20.64	-96
20.65	-34	20.65	-96
20.66	-33	20.66	-95
20.67	-33	20.67	-94
20.68	-33	20.68	-94
20.68	-32	20.68	-93
20.69	-32	20.69	-94
20.70	-31	20.70	-94
20.71	-30	20.71	-93
20.72	-29	20.72	-92

20.73	-28	20.73	-91
20.73	-28	20.73	-91
20.74	-28	20.74	-91
20.75	-28	20.75	-91
20.76	-28	20.76	-91
20.77	-27	20.77	-90
20.78	-27	20.78	-90
20.78	-26	20.78	-89
20.79	-24	20.79	-88
20.80	-23	20.80	-87
20.81	-22	20.81	-87
20.82	-21	20.82	-87
20.83	-20	20.83	-87
20.83	-19	20.83	-87
20.84	-19	20.84	-87
20.85	-18	20.85	-87
20.86	-17	20.86	-87
20.87	-16	20.87	-86
20.88	-15	20.88	-85
20.88	-14	20.88	-84
20.89	-14	20.89	-83
20.90	-14	20.90	-82
20.91	-12	20.91	-82
20.92	-11	20.92	-82
20.93	-9	20.93	-82
20.93	-9	20.93	-82
20.94	-8	20.94	-82
20.95	-7	20.95	-82
20.96	-6	20.96	-82
20.97	-4	20.97	-81
20.98	-3	20.98	-80
20.98	-2	20.98	-79
20.99	-1	20.99	-78
21.00	0	21.00	-77
21.01	2	21.01	-76
21.02	3	21.02	-75
21.03	4	21.03	-74
21.03	5	21.03	-73
21.04	7	21.04	-72
21.05	8	21.05	-71
21.06	10	21.06	-70
21.07	11	21.07	-69
21.08	13	21.08	-69

21.08	14	21.08	-69
21.09	15	21.09	-69
21.10	16	21.10	-69
21.11	16	21.11	-68
21.12	17	21.12	-68
21.13	18	21.13	-67
21.13	18	21.13	-67
21.14	19	21.14	-66
21.15	20	21.15	-65
21.16	21	21.16	-65
21.17	22	21.17	-64
21.18	24	21.18	-64
21.18	25	21.18	-63
21.19	27	21.19	-63
21.20	28	21.20	-62
21.21	29	21.21	-62
21.22	31	21.22	-61
21.23	33	21.23	-60
21.23	34	21.23	-59
21.24	36	21.24	-58
21.25	37	21.25	-57
21.26	38	21.26	-57
21.27	40	21.27	-56
21.28	42	21.28	-55
21.28	43	21.28	-54
21.29	45	21.29	-53
21.30	46	21.30	-52
21.31	47	21.31	-51
21.32	48	21.32	-51
21.33	50	21.33	-50
21.33	51	21.33	-50
21.34	53	21.34	-50
21.35	55	21.35	-49
21.36	57	21.36	-49
21.37	59	21.37	-49
21.38	61	21.38	-48
21.38	63	21.38	-48
21.39	65	21.39	-48
21.40	66	21.40	-48
21.41	68	21.41	-47
21.42	70	21.42	-46
21.43	72	21.43	-45
21.43	74	21.43	-44

21.44	75	21.44	-43
21.45	77	21.45	-42
21.46	79	21.46	-41
21.47	81	21.47	-40
21.48	82	21.48	-39
21.48	83	21.48	-39
21.49	85	21.49	-38
21.50	86	21.50	-37
21.51	88	21.51	-37
21.52	90	21.52	-36
21.53	93	21.53	-35
21.53	94	21.53	-35
21.54	95	21.54	-34
21.55	96	21.55	-34
21.56	97	21.56	-33
21.57	98	21.57	-32
21.58	100	21.58	-31
21.58	102	21.58	-29
21.59	104	21.59	-28
21.60	105	21.60	-27
21.61	106	21.61	-26
21.62	107	21.62	-25
21.63	109	21.63	-23
21.63	111	21.63	-22
21.64	112	21.64	-21
21.65	114	21.65	-20
21.66	115	21.66	-19
21.67	117	21.67	-18
21.68	119	21.68	-17
21.68	122	21.68	-16
21.69	124	21.69	-15
21.70	125	21.70	-15
21.71	127	21.71	-14
21.72	129	21.72	-14
21.73	131	21.73	-13
21.73	133	21.73	-12
21.74	135	21.74	-11
21.75	136	21.75	-10
21.76	138	21.76	-9
21.77	139	21.77	-8
21.78	140	21.78	-7
21.78	140	21.78	-7
21.79	141	21.79	-6

21.80	143	21.80	-5
21.81	144	21.81	-5
21.82	146	21.82	-4
21.83	148	21.83	-3
21.83	150	21.83	-2
21.84	151	21.84	-1
21.85	152	21.85	0
21.86	154	21.86	1
21.87	155	21.87	2
21.88	157	21.88	4
21.88	159	21.88	5
21.89	160	21.89	6
21.90	161	21.90	7
21.91	162	21.91	8
21.92	163	21.92	8
21.93	165	21.93	9
21.93	167	21.93	10
21.94	169	21.94	11
21.95	170	21.95	12
21.96	171	21.96	13
21.97	173	21.97	14
21.98	173	21.98	15
21.98	174	21.98	16
21.99	176	21.99	16
22.00	177	22.00	17
22.01	179	22.01	19
22.02	180	22.02	20
22.03	181	22.03	21
22.03	183	22.03	22
22.04	184	22.04	23
22.05	186	22.05	25
22.06	187	22.06	26
22.07	189	22.07	27
22.08	192	22.08	28
22.08	194	22.08	28
22.09	195	22.09	29
22.10	197	22.10	29
22.11	199	22.11	30
22.12	200	22.12	31
22.13	201	22.13	32
22.13	203	22.13	32
22.14	204	22.14	33
22.15	206	22.15	34

22.16	207	22.16	35
22.17	208	22.17	36
22.18	209	22.18	38
22.18	210	22.18	38
22.19	211	22.19	40
22.20	213	22.20	41
22.21	215	22.21	42
22.22	217	22.22	44
22.23	218	22.23	45
22.23	220	22.23	46
22.24	221	22.24	47
22.25	223	22.25	48
22.26	224	22.26	48
22.27	225	22.27	48
22.28	226	22.28	49
22.28	227	22.28	51
22.29	229	22.29	53
22.30	230	22.30	54
22.31	231	22.31	56
22.32	232	22.32	57
22.33	233	22.33	58
22.33	234	22.33	60
22.34	235	22.34	62
22.35	237	22.35	63
22.36	239	22.36	64
22.37	241	22.37	65
22.38	243	22.38	66
22.38	245	22.38	67
22.39	246	22.39	68
22.40	247	22.40	69
22.41	248	22.41	71
22.42	249	22.42	72
22.43	250	22.43	73
22.43	252	22.43	74
22.44	254	22.44	76
22.45	256	22.45	78
22.46	257	22.46	80
22.47	258	22.47	82
22.48	260	22.48	83
22.48	261	22.48	85
22.49	262	22.49	85
22.50	263	22.50	86
22.51	264	22.51	87

22.52	265	22.52	88
22.53	266	22.53	89
22.53	267	22.53	91
22.54	268	22.54	91
22.55	270	22.55	92
22.56	270	22.56	93
22.57	271	22.57	95
22.58	273	22.58	96
22.58	275	22.58	98
22.59	276	22.59	99
22.60	277	22.60	100
22.61	279	22.61	102
22.62	280	22.62	103
22.63	281	22.63	105
22.63	282	22.63	107
22.64	283	22.64	108
22.65	284	22.65	109
22.66	285	22.66	111
22.67	287	22.67	112
22.68	289	22.68	113
22.68	290	22.68	114
22.69	292	22.69	116
22.70	293	22.70	118
22.71	295	22.71	119
22.72	296	22.72	120
22.73	297	22.73	122
22.73	299	22.73	124
22.74	300	22.74	125
22.75	301	22.75	126
22.76	303	22.76	127
22.77	304	22.77	127
22.78	305	22.78	128
22.78	305	22.78	130
22.79	306	22.79	131
22.80	307	22.80	133
22.81	309	22.81	134
22.82	311	22.82	136
22.83	312	22.83	137
22.83	314	22.83	139
22.84	316	22.84	140
22.85	318	22.85	142
22.86	320	22.86	144
22.87	322	22.87	146

22.88	323	22.88	148
22.88	325	22.88	150
22.89	326	22.89	151
22.90	328	22.90	152
22.91	330	22.91	154
22.92	332	22.92	156
22.93	334	22.93	158
22.93	336	22.93	160
22.94	337	22.94	162
22.95	339	22.95	163
22.96	340	22.96	165
22.97	342	22.97	167
22.98	343	22.98	169
22.98	345	22.98	171
22.99	348	22.99	172
23.00	350	23.00	173
23.01	351	23.01	175
23.02	353	23.02	176
23.03	355	23.03	177
23.03	357	23.03	179
23.04	359	23.04	181
23.05	361	23.05	184
23.06	363	23.06	186
23.07	365	23.07	188
23.08	367	23.08	190
23.08	369	23.08	192
23.09	371	23.09	194
23.10	373	23.10	196
23.11	374	23.11	197
23.12	376	23.12	198
23.13	377	23.13	200
23.13	379	23.13	202
23.14	382	23.14	203
23.15	384	23.15	206
23.16	387	23.16	208
23.17	390	23.17	210
23.18	392	23.18	212
23.18	394	23.18	215
23.19	396	23.19	217
23.20	397	23.20	219
23.21	399	23.21	221
23.22	400	23.22	223
23.23	403	23.23	225

23.23	404	23.23	226
23.24	406	23.24	228
23.25	408	23.25	230
23.26	411	23.26	232
23.27	413	23.27	234
23.28	416	23.28	236
23.28	419	23.28	238
23.29	422	23.29	240
23.30	424	23.30	243
23.31	426	23.31	245
23.32	429	23.32	247
23.33	432	23.33	249
23.33	434	23.33	250
23.34	436	23.34	252
23.35	439	23.35	253
23.36	441	23.36	255
23.37	443	23.37	257
23.38	446	23.38	259
23.38	448	23.38	261
23.39	450	23.39	264
23.40	453	23.40	266
23.41	455	23.41	268
23.42	457	23.42	270
23.43	459	23.43	271
23.43	461	23.43	272
23.44	463	23.44	274
23.45	464	23.45	276
23.46	466	23.46	279
23.47	468	23.47	281
23.48	470	23.48	283
23.48	473	23.48	285
23.49	475	23.49	287
23.50	477	23.50	289
23.51	480	23.51	291
23.52	483	23.52	294
23.53	486	23.53	297
23.53	488	23.53	299
23.54	490	23.54	301
23.55	492	23.55	303
23.56	494	23.56	304
23.57	497	23.57	306
23.58	500	23.58	308
23.58	503	23.58	310

23.59	506	23.59	312
23.60	509	23.60	313
23.61	511	23.61	315
23.62	514	23.62	317
23.63	516	23.63	319
23.63	518	23.63	321
23.64	520	23.64	323
23.65	522	23.65	325
23.66	525	23.66	327
23.67	527	23.67	329
23.68	530	23.68	331
23.68	531	23.68	333
23.69	533	23.69	335
23.70	535	23.70	337
23.71	537	23.71	340
23.72	540	23.72	342
23.73	542	23.73	344
23.73	544	23.73	346
23.74	546	23.74	348
23.75	548	23.75	350
23.76	550	23.76	352
23.77	552	23.77	354
23.78	554	23.78	356
23.78	556	23.78	358
23.79	558	23.79	360
23.80	560	23.80	362
23.81	562	23.81	364
23.82	564	23.82	367
23.83	565	23.83	369
23.83	567	23.83	371
23.84	569	23.84	373
23.85	571	23.85	375
23.86	573	23.86	377
23.87	576	23.87	380
23.88	578	23.88	382
23.88	580	23.88	384
23.89	582	23.89	387
23.90	584	23.90	389
23.91	586	23.91	391
23.92	588	23.92	392
23.93	591	23.93	395
23.93	593	23.93	397
23.94	594	23.94	399

23.95	595	23.95	402
23.96	597	23.96	404
23.97	599	23.97	406
23.98	602	23.98	408
23.98	604	23.98	410
23.99	607	23.99	412
24.00	609	24.00	415
24.01	611	24.01	418
24.02	612	24.02	420
24.03	615	24.03	422
24.03	616	24.03	423
24.04	619	24.04	425
24.05	621	24.05	426
24.06	623	24.06	428
24.07	625	24.07	431
24.08	626	24.08	433
24.08	627	24.08	436
24.09	628	24.09	437
24.10	629	24.10	440
24.11	630	24.11	442
24.12	632	24.12	444
24.13	634	24.13	446
24.13	636	24.13	448
24.14	638	24.14	449
24.15	639	24.15	451
24.16	641	24.16	452
24.17	642	24.17	454
24.18	643	24.18	455
24.18	645	24.18	457
24.19	647	24.19	459
24.20	649	24.20	460
24.21	650	24.21	462
24.22	652	24.22	463
24.23	653	24.23	465
24.23	656	24.23	466
24.24	657	24.24	468
24.25	659	24.25	470
24.26	660	24.26	472
24.27	661	24.27	475
24.28	662	24.28	476
24.28	664	24.28	478
24.29	666	24.29	480
24.30	667	24.30	481

24.31	669	24.31	482
24.32	671	24.32	483
24.33	672	24.33	485
24.33	673	24.33	486
24.34	675	24.34	488
24.35	676	24.35	490
24.36	678	24.36	492
24.37	680	24.37	494
24.38	681	24.38	496
24.38	682	24.38	497
24.39	684	24.39	499
24.40	685	24.40	501
24.41	688	24.41	503
24.42	690	24.42	504
24.43	691	24.43	506
24.43	693	24.43	508
24.44	694	24.44	510
24.45	696	24.45	512
24.46	698	24.46	513
24.47	699	24.47	515
24.48	700	24.48	516
24.48	701	24.48	517
24.49	703	24.49	519
24.50	704	24.50	520
24.51	705	24.51	521
24.52	706	24.52	523
24.53	707	24.53	524
24.53	707	24.53	525
24.54	707	24.54	527
24.55	708	24.55	529
24.56	709	24.56	531
24.57	710	24.57	533
24.58	711	24.58	536
24.58	712	24.58	538
24.59	714	24.59	539
24.60	716	24.60	541
24.61	717	24.61	543
24.62	719	24.62	544
24.63	720	24.63	545
24.63	721	24.63	546
24.64	722	24.64	547
24.65	724	24.65	548
24.66	725	24.66	549

24.67	727	24.67	550
24.68	727	24.68	552
24.68	728	24.68	553
24.69	728	24.69	553
24.70	729	24.70	555
24.71	729	24.71	557
24.72	730	24.72	558
24.73	731	24.73	560
24.73	732	24.73	561
24.74	733	24.74	561
24.75	733	24.75	563
24.76	734	24.76	564
24.77	735	24.77	565
24.78	736	24.78	566
24.78	736	24.78	566
24.79	737	24.79	567
24.80	737	24.80	567
24.81	737	24.81	568
24.82	737	24.82	569
24.83	737	24.83	570
24.83	738	24.83	572
24.84	738	24.84	573
24.85	739	24.85	574
24.86	739	24.86	575
24.87	739	24.87	577
24.88	739	24.88	578
24.88	739	24.88	579
24.89	739	24.89	580
24.90	740	24.90	580
24.91	740	24.91	581
24.92	741	24.92	582
24.93	742	24.93	584
24.93	742	24.93	585
24.94	742	24.94	586
24.95	742	24.95	586
24.96	743	24.96	587
24.97	743	24.97	588
24.98	743	24.98	588
24.98	743	24.98	589
24.99	744	24.99	590
25.00	744	25.00	592
25.01	744	25.01	593
25.02	744	25.02	594

25.03	744	25.03	594
25.03	744	25.03	595
25.04	745	25.04	594
25.05	744	25.05	595
25.06	744	25.06	596
25.07	744	25.07	597
25.08	744	25.08	598
25.08	744	25.08	598
25.09	745	25.09	598
25.10	744	25.10	599
25.11	744	25.11	599
25.12	743	25.12	600
25.13	743	25.13	601
25.13	743	25.13	602
25.14	743	25.14	602
25.15	743	25.15	603
25.16	743	25.16	603
25.17	743	25.17	604
25.18	743	25.18	604
25.18	742	25.18	606
25.19	742	25.19	607
25.20	742	25.20	608
25.21	742	25.21	609
25.22	741	25.22	609
25.23	742	25.23	609
25.23	742	25.23	609
25.24	743	25.24	609
25.25	743	25.25	609
25.26	743	25.26	609
25.27	743	25.27	610
25.28	743	25.28	611
25.28	742	25.28	612
25.29	742	25.29	614
25.30	742	25.30	615
25.31	742	25.31	615
25.32	743	25.32	614
25.33	743	25.33	615
25.33	742	25.33	616
25.34	742	25.34	616
25.35	741	25.35	617
25.36	741	25.36	618
25.37	741	25.37	620
25.38	742	25.38	621

25.38	742	25.38	621
25.39	742	25.39	622
25.40	743	25.40	623
25.41	743	25.41	624
25.42	744	25.42	624
25.43	745	25.43	625
25.43	746	25.43	626
25.44	746	25.44	627
25.45	747	25.45	628
25.46	748	25.46	629
25.47	749	25.47	630
25.48	750	25.48	630
25.48	751	25.48	631
25.49	751	25.49	632
25.50	752	25.50	633
25.51	753	25.51	635
25.52	754	25.52	636
25.53	755	25.53	636
25.53	756	25.53	637
25.54	757	25.54	638
25.55	758	25.55	639
25.56	759	25.56	640
25.57	759	25.57	640
25.58	759	25.58	641
25.58	760	25.58	641
25.59	762	25.59	642
25.60	763	25.60	644
25.61	764	25.61	645
25.62	764	25.62	647
25.63	765	25.63	648
25.63	766	25.63	649
25.64	767	25.64	650
25.65	768	25.65	651
25.66	769	25.66	652
25.67	770	25.67	654
25.68	771	25.68	656
25.68	773	25.68	657
25.69	774	25.69	657
25.70	776	25.70	658
25.71	777	25.71	658
25.72	778	25.72	660
25.73	779	25.73	661
25.73	779	25.73	663

25.74	780	25.74	665
25.75	781	25.75	666
25.76	782	25.76	669
25.77	784	25.77	670
25.78	785	25.78	671
25.78	786	25.78	672
25.79	786	25.79	673
25.80	787	25.80	674
25.81	787	25.81	676
25.82	788	25.82	678
25.83	788	25.83	680
25.83	789	25.83	681
25.84	790	25.84	682
25.85	791	25.85	684
25.86	793	25.86	686
25.87	794	25.87	687
25.88	795	25.88	688
25.88	795	25.88	689
25.89	796	25.89	689
25.90	797	25.90	690
25.91	797	25.91	691
25.92	798	25.92	691
25.93	798	25.93	692
25.93	798	25.93	692
25.94	798	25.94	693
25.95	799	25.95	694
25.96	799	25.96	695
25.97	800	25.97	696
25.98	800	25.98	697
25.98	800	25.98	698
25.99	800	25.99	699
26.00	800	26.00	701
26.01	799	26.01	702
26.02	800	26.02	704
26.03	800	26.03	705
26.03	799	26.03	706
26.04	800	26.04	706
26.05	800	26.05	707
26.06	800	26.06	706
26.07	799	26.07	706
26.08	798	26.08	706
26.08	798	26.08	707
26.09	798	26.09	708

26.10	798	26.10	709
26.11	798	26.11	710
26.12	798	26.12	711
26.13	797	26.13	711
26.13	797	26.13	711
26.14	796	26.14	712
26.15	795	26.15	712
26.16	794	26.16	712
26.17	793	26.17	712
26.18	793	26.18	712
26.18	793	26.18	712
26.19	793	26.19	712
26.20	793	26.20	712
26.21	793	26.21	711
26.22	792	26.22	712
26.23	792	26.23	712
26.23	791	26.23	711
26.24	790	26.24	711
26.25	789	26.25	711
26.26	788	26.26	711
26.27	788	26.27	711
26.28	787	26.28	711
26.28	786	26.28	711
26.29	785	26.29	711
26.30	784	26.30	710
26.31	782	26.31	710
26.32	781	26.32	710
26.33	780	26.33	710
26.33	779	26.33	709
26.34	778	26.34	709
26.35	777	26.35	708
26.36	776	26.36	708
26.37	775	26.37	709
26.38	775	26.38	710
26.38	774	26.38	710
26.39	774	26.39	710
26.40	773	26.40	710
26.41	772	26.41	710
26.42	771	26.42	710
26.43	769	26.43	710
26.43	768	26.43	709
26.44	766	26.44	708
26.45	765	26.45	707

26.46	765	26.46	706
26.47	764	26.47	705
26.48	763	26.48	705
26.48	761	26.48	705
26.49	760	26.49	704
26.50	759	26.50	703
26.51	758	26.51	702
26.52	757	26.52	702
26.53	756	26.53	701
26.53	755	26.53	699
26.54	754	26.54	698
26.55	754	26.55	698
26.56	752	26.56	697
26.57	751	26.57	697
26.58	751	26.58	697
26.58	750	26.58	696
26.59	749	26.59	696
26.60	748	26.60	696
26.61	746	26.61	695
26.62	745	26.62	694
26.63	744	26.63	694
26.63	743	26.63	693
26.64	742	26.64	692
26.65	742	26.65	692
26.66	740	26.66	691
26.67	739	26.67	690
26.68	737	26.68	689
26.68	736	26.68	688
26.69	734	26.69	687
26.70	733	26.70	686
26.71	731	26.71	685
26.72	730	26.72	684
26.73	728	26.73	684
26.73	727	26.73	684
26.74	726	26.74	683
26.75	725	26.75	682
26.76	723	26.76	682
26.77	721	26.77	681
26.78	718	26.78	680
26.78	716	26.78	679
26.79	714	26.79	678
26.80	712	26.80	677
26.81	711	26.81	676

26.82	709	26.82	675
26.83	708	26.83	674
26.83	706	26.83	674
26.84	705	26.84	673
26.85	703	26.85	671
26.86	702	26.86	670
26.87	701	26.87	669
26.88	700	26.88	668
26.88	699	26.88	667
26.89	698	26.89	666
26.90	697	26.90	665
26.91	696	26.91	664
26.92	694	26.92	663
26.93	692	26.93	663
26.93	691	26.93	662
26.94	690	26.94	662
26.95	689	26.95	661
26.96	688	26.96	660
26.97	686	26.97	659
26.98	684	26.98	659
26.98	682	26.98	658
26.99	680	26.99	656
27.00	679	27.00	655
27.01	678	27.01	653
27.02	676	27.02	652
27.03	675	27.03	652
27.03	673	27.03	651
27.04	671	27.04	650
27.05	669	27.05	649
27.06	668	27.06	647
27.07	667	27.07	646
27.08	665	27.08	644
27.08	664	27.08	642
27.09	663	27.09	641
27.10	662	27.10	640
27.11	661	27.11	639
27.12	660	27.12	637
27.13	659	27.13	636
27.13	657	27.13	635
27.14	656	27.14	634
27.15	654	27.15	633
27.16	652	27.16	632
27.17	650	27.17	632

27.18	649	27.18	630
27.18	647	27.18	629
27.19	645	27.19	628
27.20	643	27.20	627
27.21	641	27.21	626
27.22	640	27.22	625
27.23	638	27.23	625
27.23	636	27.23	624
27.24	635	27.24	623
27.25	634	27.25	622
27.26	633	27.26	620
27.27	632	27.27	619
27.28	631	27.28	618
27.28	629	27.28	617
27.29	628	27.29	615
27.30	626	27.30	614
27.31	623	27.31	613
27.32	621	27.32	612
27.33	618	27.33	610
27.33	616	27.33	609
27.34	615	27.34	608
27.35	614	27.35	607
27.36	612	27.36	606
27.37	611	27.37	604
27.38	610	27.38	604
27.38	608	27.38	602
27.39	607	27.39	601
27.40	605	27.40	600
27.41	603	27.41	598
27.42	601	27.42	597
27.43	599	27.43	596
27.43	597	27.43	595
27.44	596	27.44	595
27.45	596	27.45	595
27.46	595	27.46	595
27.47	594	27.47	594
27.48	593	27.48	593
27.48	592	27.48	592
27.49	591	27.49	591
27.50	590	27.50	590
27.51	588	27.51	588
27.52	587	27.52	587
27.53	585	27.53	585

27.53	583	27.53	584
27.54	581	27.54	582
27.55	580	27.55	580
27.56	579	27.56	579
27.57	577	27.57	577
27.58	575	27.58	576
27.58	574	27.58	574
27.59	572	27.59	574
27.60	570	27.60	573
27.61	568	27.61	571
27.62	567	27.62	570
27.63	565	27.63	569
27.63	563	27.63	568
27.64	562	27.64	567
27.65	561	27.65	566
27.66	560	27.66	565
27.67	559	27.67	564
27.68	558	27.68	562
27.68	557	27.68	561
27.69	556	27.69	561
27.70	554	27.70	560
27.71	552	27.71	558
27.72	550	27.72	557
27.73	548	27.73	555
27.73	546	27.73	553
27.74	544	27.74	552
27.75	543	27.75	550
27.76	542	27.76	549
27.77	541	27.77	547
27.78	539	27.78	546
27.78	538	27.78	544
27.79	537	27.79	544
27.80	536	27.80	543
27.81	536	27.81	542
27.82	535	27.82	541
27.83	534	27.83	539
27.83	533	27.83	537
27.84	531	27.84	536
27.85	529	27.85	534
27.86	527	27.86	534
27.87	525	27.87	533
27.88	523	27.88	531
27.88	521	27.88	529

27.89	519	27.89	528
27.90	518	27.90	526
27.91	517	27.91	525
27.92	516	27.92	523
27.93	515	27.93	522
27.93	513	27.93	521
27.94	511	27.94	520
27.95	510	27.95	519
27.96	509	27.96	518
27.97	508	27.97	517
27.98	506	27.98	516
27.98	505	27.98	514
27.99	503	27.99	513
28.00	502	28.00	512
28.01	501	28.01	511
28.02	500	28.02	509
28.03	500	28.03	508
28.03	499	28.03	507
28.04	498	28.04	505
28.05	496	28.05	504
28.06	495	28.06	503
28.07	494	28.07	502
28.08	493	28.08	501
28.08	493	28.08	501
28.09	491	28.09	500
28.10	490	28.10	498
28.11	489	28.11	497
28.12	488	28.12	496
28.13	486	28.13	495
28.13	485	28.13	495
28.14	484	28.14	494
28.15	483	28.15	493
28.16	482	28.16	492
28.17	482	28.17	491
28.18	481	28.18	490
28.18	480	28.18	490
28.19	478	28.19	489
28.20	477	28.20	488
28.21	476	28.21	487
28.22	475	28.22	486
28.23	474	28.23	485
28.23	473	28.23	484
28.24	473	28.24	483

28.25	473	28.25	482
28.26	472	28.26	481
28.27	470	28.27	480
28.28	470	28.28	480
28.28	470	28.28	479
28.29	469	28.29	479
28.30	468	28.30	478
28.31	467	28.31	478
28.32	466	28.32	477
28.33	465	28.33	476
28.33	464	28.33	476
28.34	464	28.34	475
28.35	463	28.35	475
28.36	462	28.36	474
28.37	461	28.37	474
28.38	460	28.38	473
28.38	460	28.38	472
28.39	459	28.39	472
28.40	459	28.40	472
28.41	459	28.41	471
28.42	460	28.42	471
28.43	460	28.43	469
28.43	460	28.43	468
28.44	459	28.44	467
28.45	459	28.45	466
28.46	457	28.46	465
28.47	455	28.47	464
28.48	454	28.48	463
28.48	453	28.48	463
28.49	452	28.49	463
28.50	452	28.50	462
28.51	451	28.51	461
28.52	451	28.52	460
28.53	451	28.53	459
28.53	451	28.53	458
28.54	450	28.54	458
28.55	450	28.55	458
28.56	449	28.56	457
28.57	448	28.57	457
28.58	448	28.58	456
28.58	447	28.58	455
28.59	446	28.59	454
28.60	445	28.60	454

28.61	444	28.61	454
28.62	443	28.62	454
28.63	443	28.63	454
28.63	442	28.63	453
28.64	441	28.64	453
28.65	440	28.65	452
28.66	440	28.66	452
28.67	439	28.67	451
28.68	439	28.68	449
28.68	438	28.68	448
28.69	438	28.69	448
28.70	438	28.70	447
28.71	438	28.71	446
28.72	437	28.72	446
28.73	437	28.73	446
28.73	436	28.73	446
28.74	435	28.74	446
28.75	435	28.75	445
28.76	434	28.76	444
28.77	433	28.77	443
28.78	433	28.78	442
28.78	432	28.78	441
28.79	431	28.79	440
28.80	430	28.80	440
28.81	430	28.81	439
28.82	430	28.82	439
28.83	429	28.83	438
28.83	428	28.83	438
28.84	427	28.84	437
28.85	426	28.85	436
28.86	425	28.86	436
28.87	424	28.87	436
28.88	424	28.88	435
28.88	424	28.88	434
28.89	423	28.89	433
28.90	423	28.90	431
28.91	423	28.91	430
28.92	423	28.92	429
28.93	422	28.93	428
28.93	421	28.93	427
28.94	420	28.94	426
28.95	420	28.95	425
28.96	419	28.96	424

28.97	419	28.97	423
28.98	418	28.98	423
28.98	417	28.98	422
28.99	416	28.99	422
29.00	415	29.00	420
29.01	413	29.01	418
29.02	412	29.02	417
29.03	411	29.03	416
29.03	410	29.03	414
29.04	409	29.04	414
29.05	408	29.05	412
29.06	407	29.06	411
29.07	405	29.07	409
29.08	404	29.08	409
29.08	403	29.08	408
29.09	402	29.09	407
29.10	400	29.10	405
29.11	397	29.11	404
29.12	394	29.12	403
29.13	392	29.13	401
29.13	390	29.13	399
29.14	388	29.14	397
29.15	386	29.15	395
29.16	383	29.16	394
29.17	380	29.17	392
29.18	377	29.18	389
29.18	374	29.18	387
29.19	371	29.19	384
29.20	368	29.20	381
29.21	364	29.21	379
29.22	360	29.22	376
29.23	355	29.23	373
29.23	350	29.23	369
29.24	345	29.24	365
29.25	340	29.25	361
29.26	334	29.26	356
29.27	328	29.27	350
29.28	321	29.28	344
29.28	314	29.28	337
29.29	305	29.29	330
29.30	295	29.30	322
29.31	285	29.31	313
29.32	273	29.32	304

29.33	261	29.33	294
29.33	248	29.33	282
29.34	233	29.34	270
29.35	217	29.35	256
29.36	200	29.36	241
29.37	181	29.37	225
29.38	160	29.38	206
29.38	137	29.38	185
29.39	112	29.39	162
29.40	85	29.40	138
29.41	55	29.41	111
29.42	23	29.42	81
29.43	-12	29.43	49
29.43	-50	29.43	14
29.44	-92	29.44	-24
29.45	-137	29.45	-65
29.46	-187	29.46	-109
29.47	-240	29.47	-157
29.48	-297	29.48	-208
29.48	-358	29.48	-264
29.49	-424	29.49	-325
29.50	-494	29.50	-389
29.51	-569	29.51	-457
29.52	-649	29.52	-531
29.53	-733	29.53	-609
29.53	-824	29.53	-693
29.54	-919	29.54	-781
29.55	-1021	29.55	-875
29.56	-1127	29.56	-974
29.57	-1239	29.57	-1079
29.58	-1358	29.58	-1191
29.58	-1482	29.58	-1307
29.59	-1611	29.59	-1429
29.60	-1746	29.60	-1556
29.61	-1887	29.61	-1690
29.62	-2032	29.62	-1829
29.63	-2183	29.63	-1973
29.63	-2340	29.63	-2122
29.64	-2501	29.64	-2277
29.65	-2666	29.65	-2436
29.66	-2836	29.66	-2598
29.67	-3009	29.67	-2765
29.68	-3185	29.68	-2935

29.68	-3364	29.68	-3108
29.69	-3546	29.69	-3284
29.70	-3729	29.70	-3463
29.71	-3914	29.71	-3643
29.72	-4099	29.72	-3825
29.73	-4285	29.73	-4008
29.73	-4469	29.73	-4192
29.74	-4654	29.74	-4375
29.75	-4836	29.75	-4557
29.76	-5015	29.76	-4736
29.77	-5192	29.77	-4913
29.78	-5364	29.78	-5088
29.78	-5532	29.78	-5259
29.79	-5696	29.79	-5425
29.80	-5853	29.80	-5586
29.81	-6005	29.81	-5742
29.82	-6149	29.82	-5891
29.83	-6287	29.83	-6034
29.83	-6416	29.83	-6170
29.84	-6537	29.84	-6297
29.85	-6649	29.85	-6417
29.86	-6752	29.86	-6529
29.87	-6846	29.87	-6630
29.88	-6930	29.88	-6722
29.88	-7004	29.88	-6804
29.89	-7068	29.89	-6878
29.90	-7122	29.90	-6941
29.91	-7165	29.91	-6995
29.92	-7198	29.92	-7038
29.93	-7221	29.93	-7070
29.93	-7234	29.93	-7092
29.94	-7236	29.94	-7103
29.95	-7228	29.95	-7104
29.96	-7210	29.96	-7095
29.97	-7182	29.97	-7076
29.98	-7145	29.98	-7048
29.98	-7099	29.98	-7010
29.99	-7043	29.99	-6964
30.00	-6979	30.00	-6910
30.01	-6907	30.01	-6846
30.02	-6826	30.02	-6774
30.03	-6740	30.03	-6695
30.03	-6646	30.03	-6608

30.04	-6546	30.04	-6516
30.05	-6440	30.05	-6417
30.06	-6329	30.06	-6312
30.07	-6213	30.07	-6203
30.08	-6092	30.08	-6089
30.08	-5967	30.08	-5970
30.09	-5839	30.09	-5848
30.10	-5708	30.10	-5722
30.11	-5573	30.11	-5595
30.12	-5438	30.12	-5464
30.13	-5302	30.13	-5332
30.13	-5166	30.13	-5199
30.14	-5029	30.14	-5064
30.15	-4891	30.15	-4929
30.16	-4753	30.16	-4793
30.17	-4615	30.17	-4658
30.18	-4478	30.18	-4523
30.18	-4342	30.18	-4388
30.19	-4208	30.19	-4255
30.20	-4074	30.20	-4124
30.21	-3943	30.21	-3994
30.22	-3814	30.22	-3865
30.23	-3687	30.23	-3738
30.23	-3563	30.23	-3614
30.24	-3441	30.24	-3493
30.25	-3322	30.25	-3374
30.26	-3207	30.26	-3258
30.27	-3094	30.27	-3144
30.28	-2984	30.28	-3033
30.28	-2878	30.28	-2925
30.29	-2774	30.29	-2820
30.30	-2673	30.30	-2717
30.31	-2575	30.31	-2619
30.32	-2480	30.32	-2523
30.33	-2388	30.33	-2430
30.33	-2299	30.33	-2340
30.34	-2213	30.34	-2253
30.35	-2130	30.35	-2169
30.36	-2049	30.36	-2087
30.37	-1972	30.37	-2009
30.38	-1897	30.38	-1933
30.38	-1825	30.38	-1860
30.39	-1756	30.39	-1789

30.40	-1689	30.40	-1721
30.41	-1624	30.41	-1656
30.42	-1562	30.42	-1593
30.43	-1502	30.43	-1532
30.43	-1444	30.43	-1474
30.44	-1389	30.44	-1417
30.45	-1335	30.45	-1364
30.46	-1284	30.46	-1312
30.47	-1235	30.47	-1262
30.48	-1188	30.48	-1214
30.48	-1143	30.48	-1167
30.49	-1100	30.49	-1123
30.50	-1058	30.50	-1080
30.51	-1018	30.51	-1039
30.52	-979	30.52	-1000
30.53	-942	30.53	-962
30.53	-906	30.53	-925
30.54	-871	30.54	-889
30.55	-838	30.55	-855
30.56	-806	30.56	-822
30.57	-774	30.57	-789
30.58	-745	30.58	-758
30.58	-717	30.58	-728
30.59	-689	30.59	-699
30.60	-662	30.60	-672
30.61	-636	30.61	-646
30.62	-610	30.62	-620
30.63	-584	30.63	-594
30.63	-560	30.63	-570
30.64	-536	30.64	-546
30.65	-513	30.65	-524
30.66	-491	30.66	-501
30.67	-470	30.67	-480
30.68	-450	30.68	-459
30.68	-430	30.68	-438
30.69	-411	30.69	-418
30.70	-393	30.70	-399
30.71	-376	30.71	-380
30.72	-359	30.72	-362
30.73	-342	30.73	-345
30.73	-326	30.73	-328
30.74	-310	30.74	-312
30.75	-295	30.75	-296

30.76	-280	30.76	-280
30.77	-266	30.77	-265
30.78	-252	30.78	-251
30.78	-238	30.78	-237
30.79	-225	30.79	-224
30.80	-212	30.80	-209
30.81	-199	30.81	-195
30.82	-186	30.82	-181
30.83	-174	30.83	-168
30.83	-162	30.83	-156
30.84	-152	30.84	-144
30.85	-141	30.85	-133
30.86	-130	30.86	-122
30.87	-120	30.87	-111
30.88	-109	30.88	-101
30.88	-98	30.88	-91
30.89	-87	30.89	-81
30.90	-77	30.90	-72
30.91	-67	30.91	-62
30.92	-59	30.92	-53
30.93	-50	30.93	-44
30.93	-42	30.93	-35
30.94	-34	30.94	-26
30.95	-25	30.95	-18
30.96	-17	30.96	-9
30.97	-10	30.97	-1
30.98	-3	30.98	7
30.98	4	30.98	15
30.99	12	30.99	23
31.00	19	31.00	31
31.01	26	31.01	39
31.02	33	31.02	46
31.03	40	31.03	53
31.03	46	31.03	60
31.04	52	31.04	67
31.05	59	31.05	74
31.06	66	31.06	80
31.07	73	31.07	86
31.08	79	31.08	93
31.08	85	31.08	99
31.09	91	31.09	104
31.10	97	31.10	110
31.11	104	31.11	116

31.12	109	31.12	121
31.13	114	31.13	126
31.13	119	31.13	130
31.14	124	31.14	136
31.15	129	31.15	141
31.16	134	31.16	145
31.17	139	31.17	150
31.18	144	31.18	155
31.18	149	31.18	160
31.19	153	31.19	166
31.20	157	31.20	171
31.21	161	31.21	175
31.22	165	31.22	179
31.23	169	31.23	183
31.23	172	31.23	188
31.24	176	31.24	192
31.25	180	31.25	196
31.26	184	31.26	200
31.27	188	31.27	204
31.28	192	31.28	207
31.28	195	31.28	211
31.29	199	31.29	215
31.30	202	31.30	219
31.31	205	31.31	222
31.32	208	31.32	225
31.33	211	31.33	229
31.33	214	31.33	232
31.34	217	31.34	235
31.35	220	31.35	238
31.36	223	31.36	241
31.37	225	31.37	243
31.38	228	31.38	246
31.38	231	31.38	248
31.39	233	31.39	250
31.40	236	31.40	253
31.41	240	31.41	255
31.42	243	31.42	258
31.43	246	31.43	261
31.43	248	31.43	263
31.44	251	31.44	266
31.45	252	31.45	268
31.46	254	31.46	269
31.47	255	31.47	271

31.48	256	31.48	273
31.48	258	31.48	275
31.49	259	31.49	277
31.50	261	31.50	279
31.51	264	31.51	280
31.52	266	31.52	281
31.53	268	31.53	283
31.53	270	31.53	284
31.54	272	31.54	285
31.55	274	31.55	287
31.56	275	31.56	288
31.57	276	31.57	290
31.58	273	31.58	291
31.58	270	31.58	292
31.59	270	31.59	294
31.60	272	31.60	295
31.61	274	31.61	297
31.62	276	31.62	298
31.63	278	31.63	299
31.63	281	31.63	300
31.64	283	31.64	301
31.65	285	31.65	302
31.66	287	31.66	303
31.67	288	31.67	303
31.68	289	31.68	303
31.68	290	31.68	304
31.69	291	31.69	304
31.70	292	31.70	305
31.71	292	31.71	305
31.72	293	31.72	306
31.73	293	31.73	308
31.73	294	31.73	309
31.74	295	31.74	309
31.75	295	31.75	310
31.76	296	31.76	310
31.77	297	31.77	310
31.78	297	31.78	311
31.78	297	31.78	311
31.79	298	31.79	311
31.80	299	31.80	312
31.81	300	31.81	312
31.82	300	31.82	312
31.83	301	31.83	313

31.83	302	31.83	314
31.84	303	31.84	314
31.85	303	31.85	315
31.86	304	31.86	316
31.87	305	31.87	316
31.88	305	31.88	317
31.88	306	31.88	318
31.89	306	31.89	318
31.90	307	31.90	318
31.91	307	31.91	318
31.92	307	31.92	319
31.93	308	31.93	319
31.93	308	31.93	319
31.94	309	31.94	319
31.95	309	31.95	320
31.96	308	31.96	320
31.97	308	31.97	320
31.98	308	31.98	319
31.98	309	31.98	319
31.99	310	31.99	319
32.00	311	32.00	320
32.01	311	32.01	321
32.02	311	32.02	321
32.03	312	32.03	321
32.03	312	32.03	322
32.04	313	32.04	323
32.05	314	32.05	323
32.06	315	32.06	323
32.07	316	32.07	323
32.08	317	32.08	323
32.08	318	32.08	324
32.09	318	32.09	324
32.10	318	32.10	324
32.11	319	32.11	324
32.12	320	32.12	325
32.13	321	32.13	325
32.13	321	32.13	326
32.14	321	32.14	326
32.15	322	32.15	326
32.16	322	32.16	327
32.17	322	32.17	328
32.18	322	32.18	328
32.18	322	32.18	328

32.19	322	32.19	329
32.20	321	32.20	329
32.21	322	32.21	329
32.22	323	32.22	330
32.23	323	32.23	330
32.23	323	32.23	330
32.24	323	32.24	330
32.25	324	32.25	330
32.26	325	32.26	330
32.27	325	32.27	330
32.28	327	32.28	331
32.28	327	32.28	331
32.29	328	32.29	332
32.30	329	32.30	333
32.31	330	32.31	333
32.32	331	32.32	333
32.33	331	32.33	333
32.33	331	32.33	333
32.34	331	32.34	333
32.35	332	32.35	333
32.36	332	32.36	333
32.37	332	32.37	332
32.38	332	32.38	332
32.38	333	32.38	333
32.39	333	32.39	333
32.40	333	32.40	333
32.41	333	32.41	333
32.42	333	32.42	332
32.43	333	32.43	332
32.43	334	32.43	332
32.44	334	32.44	332
32.45	335	32.45	332
32.46	335	32.46	332
32.47	336	32.47	332
32.48	335	32.48	333
32.48	335	32.48	333
32.49	334	32.49	333
32.50	334	32.50	334
32.51	334	32.51	334
32.52	333	32.52	334
32.53	334	32.53	335
32.53	334	32.53	335
32.54	334	32.54	334

32.55	334	32.55	334
32.56	333	32.56	334
32.57	332	32.57	333
32.58	332	32.58	332
32.58	331	32.58	331
32.59	330	32.59	329
32.60	329	32.60	328
32.61	328	32.61	327
32.62	327	32.62	326
32.63	327	32.63	325
32.63	326	32.63	324
32.64	325	32.64	324
32.65	323	32.65	323
32.66	320	32.66	323
32.67	319	32.67	321
32.68	317	32.68	320
32.68	316	32.68	318
32.69	314	32.69	317
32.70	312	32.70	316
32.71	310	32.71	314
32.72	308	32.72	312
32.73	306	32.73	310
32.73	304	32.73	308
32.74	301	32.74	306
32.75	299	32.75	303
32.76	297	32.76	299
32.77	294	32.77	296
32.78	291	32.78	293
32.78	287	32.78	290
32.79	282	32.79	286
32.80	277	32.80	283
32.81	272	32.81	278
32.82	266	32.82	274
32.83	262	32.83	269
32.83	257	32.83	264
32.84	251	32.84	259
32.85	245	32.85	253
32.86	239	32.86	248
32.87	233	32.87	243
32.88	226	32.88	237
32.88	219	32.88	232
32.89	212	32.89	226
32.90	205	32.90	219

32.91	198	32.91	213
32.92	190	32.92	206
32.93	183	32.93	198
32.93	174	32.93	191
32.94	166	32.94	183
32.95	158	32.95	175
32.96	150	32.96	168
32.97	141	32.97	160
32.98	133	32.98	152
32.98	125	32.98	143
32.99	117	32.99	135
33.00	108	33.00	127
33.01	99	33.01	118
33.02	89	33.02	108
33.03	80	33.03	98
33.03	71	33.03	89
33.04	62	33.04	80
33.05	52	33.05	71
33.06	43	33.06	62
33.07	34	33.07	52
33.08	24	33.08	43
33.08	15	33.08	34
33.09	5	33.09	24
33.10	-4	33.10	16
33.11	-13	33.11	7
33.12	-22	33.12	-2
33.13	-31	33.13	-12
33.13	-40	33.13	-21
33.14	-49	33.14	-31
33.15	-58	33.15	-40
33.16	-67	33.16	-49
33.17	-75	33.17	-57
33.18	-83	33.18	-65
33.18	-90	33.18	-72
33.19	-97	33.19	-79
33.20	-105	33.20	-86
33.21	-111	33.21	-93
33.22	-117	33.22	-99
33.23	-123	33.23	-105
33.23	-129	33.23	-111
33.24	-134	33.24	-116
33.25	-138	33.25	-122
33.26	-141	33.26	-127

33.27	-144	33.27	-132
33.28	-147	33.28	-137
33.28	-150	33.28	-141
33.29	-154	33.29	-144
33.30	-157	33.30	-147
33.31	-159	33.31	-150
33.32	-161	33.32	-153
33.33	-162	33.33	-155
33.33	-163	33.33	-157
33.34	-163	33.34	-158
33.35	-162	33.35	-158
33.36	-162	33.36	-159
33.37	-162	33.37	-159
33.38	-161	33.38	-158
33.38	-160	33.38	-158
33.39	-160	33.39	-156
33.40	-158	33.40	-154
33.41	-154	33.41	-152
33.42	-151	33.42	-150
33.43	-148	33.43	-147
33.43	-144	33.43	-145
33.44	-140	33.44	-142
33.45	-135	33.45	-139
33.46	-131	33.46	-136
33.47	-127	33.47	-133
33.48	-122	33.48	-129
33.48	-117	33.48	-126
33.49	-112	33.49	-121
33.50	-106	33.50	-117
33.51	-101	33.51	-112
33.52	-95	33.52	-108
33.53	-89	33.53	-103
33.53	-83	33.53	-97
33.54	-78	33.54	-91
33.55	-73	33.55	-86
33.56	-66	33.56	-80
33.57	-60	33.57	-74
33.58	-53	33.58	-67
33.58	-47	33.58	-60
33.59	-39	33.59	-54
33.60	-32	33.60	-47
33.61	-26	33.61	-40
33.62	-18	33.62	-33

33.63	-11	33.63	-26
33.63	-3	33.63	-19
33.64	4	33.64	-12
33.65	10	33.65	-5
33.66	17	33.66	3
33.67	23	33.67	11
33.68	30	33.68	18
33.68	36	33.68	24
33.69	43	33.69	30
33.70	49	33.70	36
33.71	56	33.71	42
33.72	63	33.72	48
33.73	70	33.73	54
33.73	77	33.73	60
33.74	82	33.74	66
33.75	88	33.75	72
33.76	93	33.76	78
33.77	99	33.77	83
33.78	106	33.78	89
33.78	112	33.78	96
33.79	118	33.79	102
33.80	124	33.80	108
33.81	130	33.81	114
33.82	135	33.82	119
33.83	140	33.83	125
33.83	145	33.83	130
33.84	150	33.84	135
33.85	156	33.85	140
33.86	162	33.86	144
33.87	168	33.87	149
33.88	173	33.88	153
33.88	177	33.88	157
33.89	182	33.89	161
33.90	186	33.90	166
33.91	191	33.91	171
33.92	195	33.92	176
33.93	199	33.93	179
33.93	204	33.93	183
33.94	208	33.94	187
33.95	213	33.95	191
33.96	217	33.96	195
33.97	220	33.97	199
33.98	224	33.98	203

33.98	227	33.98	206
33.99	231	33.99	210
34.00	234	34.00	213
34.01	237	34.01	216
34.02	240	34.02	219
34.03	244	34.03	222
34.03	247	34.03	224
34.04	249	34.04	228
34.05	252	34.05	232
34.06	255	34.06	235
34.07	258	34.07	238
34.08	260	34.08	240
34.08	263	34.08	242
34.09	265	34.09	244
34.10	267	34.10	246
34.11	270	34.11	248
34.12	273	34.12	250
34.13	276	34.13	253
34.13	278	34.13	256
34.14	281	34.14	258
34.15	284	34.15	261
34.16	286	34.16	263
34.17	289	34.17	265
34.18	290	34.18	268
34.18	292	34.18	269
34.19	294	34.19	271
34.20	297	34.20	273
34.21	299	34.21	275
34.22	301	34.22	276
34.23	303	34.23	278
34.23	305	34.23	280
34.24	307	34.24	282
34.25	310	34.25	284
34.26	312	34.26	286
34.27	314	34.27	287
34.28	316	34.28	290
34.28	317	34.28	292
34.29	319	34.29	295
34.30	320	34.30	297
34.31	322	34.31	298
34.32	323	34.32	300
34.33	325	34.33	302
34.33	327	34.33	304

34.34	328	34.34	306
34.35	330	34.35	307
34.36	331	34.36	308
34.37	332	34.37	309
34.38	334	34.38	309
34.38	336	34.38	310
34.39	337	34.39	311
34.40	338	34.40	312
34.41	340	34.41	313
34.42	341	34.42	315
34.43	342	34.43	316
34.43	343	34.43	317
34.44	345	34.44	317
34.45	347	34.45	318
34.46	348	34.46	318
34.47	349	34.47	319
34.48	350	34.48	321
34.48	351	34.48	322
34.49	352	34.49	324
34.50	354	34.50	325
34.51	356	34.51	327
34.52	357	34.52	328
34.53	358	34.53	329
34.53	359	34.53	330
34.54	360	34.54	331
34.55	361	34.55	332
34.56	362	34.56	334
34.57	363	34.57	334
34.58	363	34.58	335
34.58	364	34.58	336
34.59	365	34.59	337
34.60	366	34.60	339
34.61	366	34.61	340
34.62	367	34.62	341
34.63	368	34.63	342
34.63	369	34.63	343
34.64	370	34.64	343
34.65	371	34.65	344
34.66	372	34.66	345
34.67	373	34.67	346
34.68	373	34.68	346
34.68	373	34.68	347
34.69	374	34.69	348

34.70	374	34.70	350
34.71	375	34.71	351
34.72	376	34.72	352
34.73	377	34.73	353
34.73	377	34.73	354
34.74	378	34.74	356
34.75	379	34.75	356
34.76	380	34.76	357
34.77	381	34.77	358
34.78	382	34.78	358
34.78	382	34.78	359
34.79	383	34.79	359
34.80	384	34.80	360
34.81	384	34.81	361
34.82	385	34.82	361
34.83	385	34.83	362
34.83	386	34.83	363
34.84	387	34.84	363
34.85	388	34.85	364
34.86	388	34.86	364
34.87	389	34.87	365
34.88	390	34.88	366
34.88	390	34.88	366
34.89	390	34.89	366
34.90	391	34.90	367
34.91	392	34.91	367
34.92	393	34.92	368
34.93	394	34.93	368
34.93	394	34.93	369
34.94	395	34.94	369
34.95	395	34.95	369
34.96	395	34.96	369
34.97	395	34.97	369
34.98	395	34.98	369
34.98	396	34.98	370
34.99	397	34.99	371
35.00	398	35.00	372

Table S4. Experimental data for Figure 3.

ΔTMP/Δt (mbar/min)										
**Ster. 0**	**Ster. 1**	**Ster. 2**	**Ster. 3**	**Ster. 4**	**Ster. 5**	**Ster. 6**	**Ster. 7**	**Ster. 8**	**Ster. 9**	**Ster. 10**
-11.568	-7.243	-4.012	-6.566	-8.754	-4.963	-4.299	-5.002	-10.630	-3.635	-5.940
-10.005	-6.462	-1.837	-5.003	-6.722	-3.088	-3.752	-2.423	-2.501	-2.579	-1.368
-9.797	-5.784	-1.798	-1.290	-4.377	-3.087	-2.619	-1.876	-2.423	-2.501	-1.290
-9.692	-4.898	-1.251	-1.251	-4.338	-2.501	-1.876	-1.876	-2.345	-2.501	-1.212
-9.015	-4.117	-1.251	-1.251	-4.260	-2.423	-1.876	-1.876	-1.876	-1.915	-0.743
-8.963	-4.012	-1.172	-1.251	-3.713	-2.423	-1.837	-1.759	-1.251	-1.876	-0.664
-8.233	-3.335	-1.133	-1.172	-3.713	-2.345	-1.837	-1.212	-1.251	-1.876	-0.664
-8.233	-3.283	-0.664	-0.625	-3.127	-2.306	-1.798	-1.172	-1.212	-1.837	-0.664
-8.181	-3.283	-0.625	-0.625	-3.087	-0.664	-1.251	-0.625	-1.172	-1.837	-0.664
-8.077	-3.231	-0.625	-0.625	-3.048	-0.664	-1.251	-0.625	-1.172	-1.798	-0.625
-8.077	-2.553	-0.625	-0.625	-2.970	-0.625	-0.782	-0.586	-0.664	-1.798	-0.625
-7.973	-2.501	-0.625	-0.586	-2.540	-0.625	-0.703	-0.547	-0.625	-1.798	-0.625
-7.504	-2.501	-0.586	-0.078	-2.501	-0.625	-0.625	-0.508	-0.625	-1.329	-0.547
-7.295	-2.501	-0.586	-0.078	-2.501	-0.625	-0.625	-0.039	-0.625	-1.329	-0.039
-7.191	-2.449	-0.078	0.000	-2.462	-0.625	-0.625	-0.039	-0.625	-1.290	-0.039
-6.618	-2.293	-0.078	0.000	-2.462	-0.117	-0.625	0.000	-0.586	-1.212	-0.039
-6.462	-1.668	-0.074	0.000	-2.423	-0.117	-0.586	0.000	-0.547	-0.782	-0.039
-6.409	-1.667	-0.039	0.000	-2.384	-0.039	-0.586	0.000	-0.078	-0.703	0.039
-5.862	-1.615	-0.039	0.000	-2.384	-0.039	-0.508	0.000	-0.078	-0.703	0.039
-5.680	-1.563	0.000	0.000	-2.384	0.000	-0.117	0.000	-0.039	-0.664	0.039
-5.002	-1.511	0.000	0.078	-1.212	0.000	-0.039	0.000	-0.039	-0.664	0.039
-4.898	-1.459	0.000	0.078	-0.664	0.000	-0.039	0.000	0.000	-0.625	0.078
-4.846	-0.938	0.039	0.078	-0.625	0.625	-0.039	0.039	0.000	-0.625	0.078
-4.794	-0.938	0.039	0.625	-0.078		-0.039	0.039	0.000	-0.039	0.547
-4.117	-0.834	0.117	0.625			0.000	0.078	0.000	0.000	0.625
-4.064	-0.782		0.625			0.000	0.117	0.039	0.000	1.172
-3.596	-0.104					0.000	0.508	0.039	0.000	
-2.501						0.039	1.094	0.039	0.000	
								0.039	0.000	
								0.625	0.039	
								1.329	0.039	
								1.368		

Table S5. Experimental data for Figure 4.

membranes

MDPI

(a)

Ster. 0		Ster. 5		Ster. 10	
TMP (mbar)	Time (min)	TMP (mbar)	Time (min)	TMP (mbar)	Time (min)
0.00	0.00	0.00	0.00	0.00	0.00
0.63	0.17	0.63	0.17	-6.25	0.00
-0.63	0.47	0.00	0.35	-6.25	0.25
-6.88	0.65	-4.38	0.52	-6.25	0.50
-11.88	0.82	-8.75	0.70	-7.50	0.77
-12.51	0.98	-7.50	0.87	-6.25	1.02
-11.88	1.17	-7.50	1.03	-8.13	1.27
-11.88	1.33	-8.75	1.20	-8.13	1.53
-11.88	1.50	-8.75	1.38	-7.50	1.78
-12.51	1.68	-9.38	1.55	-8.13	2.03
-12.51	1.85	-8.75	1.73	-8.13	2.30
-12.51	2.02	-7.50	1.90	-7.50	2.55
-12.51	2.18	-9.38	2.07	-8.75	2.82
-12.51	2.37	-8.75	2.23	-7.50	3.07
-11.88	2.53	-9.38	2.42	-8.13	3.32
-12.51	2.70	-7.50	2.58	-8.75	3.58
-11.88	2.88	-8.75	2.75	-8.13	3.83
-13.13	3.05	-8.75	2.93	-8.13	4.10
-12.51	3.23	-7.50	3.10	-8.75	4.35
-13.13	3.42	-9.38	3.27	-8.13	4.60
-12.51	3.58	-8.75	3.45	-8.75	4.87

(b)

Ster. 0		Ster. 5		Ster. 10	
TMP (mbar)	Time (min)	TMP (mbar)	Time (min)	TMP (mbar)	Time (min)
0.00	0.00	0.00	0.00	0.00	0.00
0.63	0.18	0.00	0.17	-1.88	0.25
-15.01	0.35	-13.13	0.35	-15.01	0.52
-18.76	0.52	-13.13	0.52	-14.38	0.77
-18.76	0.70	-13.76	0.68	-15.01	1.02
-18.13	0.87	-13.76	0.87	-15.01	1.27
-18.76	1.03	-13.13	1.03	-15.01	1.53
-18.76	1.22	-13.13	1.20	-15.01	1.78
-18.76	1.38	-13.76	1.37	-14.38	2.03
-18.76	1.55	-13.76	1.55	-15.01	2.30
-18.76	1.72	-13.76	1.72	-15.01	2.55
-18.13	1.92	-13.76	1.88	-15.63	2.80
-18.76	2.08	-13.76	2.07	-15.01	3.07
-18.76	2.25	-13.76	2.23	-15.01	3.32
-18.76	2.43	-15.01	2.40	-15.01	3.58
-18.13	2.60	-15.01	2.57	-15.01	3.83
-18.76	2.77	-15.01	2.75	-15.01	4.10
-19.38	2.95	-13.76	2.92	-15.01	4.35
-18.76	3.12	-13.76	3.08	-15.01	4.60
-19.38	3.28	-13.76	3.27	-15.01	4.85
-19.38	3.45	-13.76	3.43	-15.63	5.12

-12.51	3.77	-8.75	3.62	-8.75	5.12	-18.76	3.63	-13.76	3.60	-15.01	5.37
-13.13	3.93	-9.38	3.78	-8.75	5.37	-18.76	3.80	-13.76	3.78	-15.01	5.62
-12.51	4.12	-9.38	3.95	-8.75	5.63	-18.76	3.97	-13.76	3.95	-15.01	5.88
-13.13	4.28	-9.38	4.13	-7.50	5.88	-18.76	4.15	-15.01	4.12	-15.01	6.13
-13.13	4.47	-9.38	4.30	-8.13	6.13	-18.76	4.32	-15.01	4.30	-15.01	6.38
-13.13	4.63	-10.01	4.47	-8.75	6.40	-18.76	4.48	-13.76	4.47	-15.01	6.65
-13.13	4.82	-9.38	4.65	-8.13	6.65	-18.76	4.65	-15.01	4.63	-15.63	6.90
-12.51	4.98	-8.75	4.82	-8.75	6.92	-18.76	4.83	-13.76	4.80	-15.63	7.15
-12.51	5.17	-9.38	4.98	-8.13	7.17	-19.38	5.00	-15.01	4.98	-15.63	7.42
-13.13	5.33	-9.38	5.17	-8.75	7.42	-18.76	5.18	-13.76	5.15	-15.01	7.67
-13.13	5.50	-9.38	5.33	-8.13	7.67	-18.76	5.35	-13.76	5.32	-15.63	7.92
-12.51	5.68	-9.38	5.50	-8.75	7.93	-18.76	5.52	-13.76	5.50	-15.63	8.18
-12.51	5.85	-9.38	5.68	-8.13	8.18	-18.76	5.70	-13.76	5.67	-9.38	8.43
-12.51	6.03	-9.38	5.85	-8.75	8.45	-18.76	5.87	-15.01	5.83	0.00	8.70
-12.51	6.20	-9.38	6.03	-8.75	8.70	-18.76	6.03	-13.76	6.02	0.63	8.95
-13.13	6.37	-9.38	6.20	-8.75	8.95	-18.13	6.22	-15.01	6.18	0.63	9.22
-13.13	6.55	-9.38	6.37	-8.75	9.22	-18.13	6.38	-15.63	6.35	0.00	9.47
-13.13	6.72	-9.38	6.55	-8.13	9.47	-19.38	6.55	-13.76	6.53	0.63	9.72
-12.51	6.88	-10.01	6.72	-8.13	9.72	-18.76	6.73	-13.76	6.70	0.00	9.98
-12.51	7.07	-9.38	6.90	-8.75	9.98	-18.76	6.90	-15.01	6.87	0.63	10.23
-12.51	7.25	-10.01	7.07	-8.75	10.23	-18.76	7.07	-15.01	7.05	1.25	10.50
-13.13	7.42	-10.01	7.23	-8.75	10.50	-18.76	7.23	-13.76	7.22	-1.25	10.75
-13.13	7.60	-8.75	7.42	0.00	10.75	-18.76	7.42	-15.63	7.38	-15.63	11.00
-12.51	7.77	-10.01	7.58	0.00	11.00	-19.38	7.58	-15.63	7.57	-15.01	11.27
-12.51	7.95	-9.38	7.75	0.63	11.27	-18.76	7.77	-15.01	7.73	-15.01	11.52
-11.88	8.12	-9.38	7.93	0.63	11.52	-19.38	7.93	-15.01	7.92	-15.01	11.77
-13.13	8.30	-9.38	8.10	0.63	11.77	-18.76	8.10	-15.01	8.08	-16.88	12.03
-13.13	8.48	-8.75	8.28	0.63	12.02	-18.76	8.28	-13.76	8.25	-15.63	12.28
-12.51	8.65	-8.75	8.45	0.63	12.28	-3.75	8.45	-15.01	8.42	-15.63	12.53

-13.13	8.82	-9.38	8.62	0.00	12.53	0.63	8.63	-0.63	8.60	-15.63	12.80
-13.13	9.00	-10.01	8.80	0.00	12.78	0.63	8.80	0.00	8.77	-15.63	13.05
-13.13	9.17	-10.01	8.97	0.00	13.03	0.00	8.97	0.00	8.93	-15.63	13.30
-12.51	9.33	-9.38	9.13	0.63	13.30	0.63	9.15	0.00	9.13	-15.01	13.57
-13.13	9.52	-8.75	9.30	-8.75	13.55	0.00	9.32	0.00	9.30	-15.01	13.82
-13.13	9.68	-9.38	9.48	-8.75	13.80	0.00	9.48	0.00	9.47	-15.63	14.07
-13.13	9.85	-10.01	9.65	-8.75	14.07	0.63	9.67	0.00	9.65	-15.63	14.32
-13.13	10.03	-9.38	9.82	-8.13	14.32	0.00	9.83	0.63	9.82	-15.63	14.58
-12.51	10.22	-9.38	10.00	-8.13	14.57	0.63	10.00	0.63	9.98	-15.63	14.83
-3.75	10.38	-9.38	10.17	-8.75	14.83	0.00	10.18	-0.63	10.15	-15.63	15.08
-0.63	10.55	-9.38	10.33	-8.75	15.08	0.63	10.35	0.00	10.33	-15.63	15.35
0.00	10.73	-4.38	10.52	-9.38	15.33	0.00	10.53	0.00	10.50	-16.88	15.60
-0.63	10.90	-0.63	10.68	-8.13	15.58	0.63	10.70	-0.63	10.68	-16.88	15.85
0.00	11.07	-0.63	10.85	-8.13	15.85	0.00	10.87	-9.38	10.87	-16.88	16.12
0.00	11.25	0.00	11.03	-8.75	16.10	-15.63	11.03	-13.76	11.03	-16.88	16.37
-0.63	11.42	0.00	11.20	-8.75	16.35	-18.13	11.22	-15.63	11.22	-15.63	16.62
-0.63	11.58	0.00	11.37	-8.75	16.60	-18.76	11.38	-13.76	11.38	-15.63	16.88
-0.63	11.77	0.63	11.53	-8.13	16.85	-18.76	11.55	-15.01	11.55	-15.63	17.13
0.00	11.93	0.00	11.72	-8.13	17.12	-18.76	11.73	-13.76	11.73	-16.88	17.38
-0.63	12.10	0.00	11.88	-8.75	17.37	-18.76	11.90	-15.01	11.90	-15.63	17.63
0.00	12.27	0.00	12.05	-8.75	17.63	-18.13	12.07	-15.01	12.07	-16.88	17.90
0.00	12.45	0.00	12.22	-8.75	17.88	-18.76	12.25	-15.01	12.25	-16.88	18.15
-0.63	12.63	-0.63	12.40	-8.75	18.13	-18.76	12.42	-15.01	12.42	-16.88	18.40
-11.88	12.82	-0.63	12.57	-8.75	18.40	-19.38	12.60	-15.01	12.58	-16.88	18.65
-13.13	12.98	-0.63	12.75	-9.38	18.65	-18.76	12.77	-13.76	12.77	-11.88	18.92
-13.13	13.17	-8.75	12.92	-9.38	18.90	-18.76	12.93	-15.63	12.93	0.00	19.17
-13.13	13.33	-8.75	13.08	-8.13	19.17	-18.76	13.12	-15.63	13.10	0.63	19.42
-13.13	13.50	-9.38	13.27	-9.38	19.42	-18.76	13.28	-15.01	13.27	0.63	19.68
-12.51	13.68	-9.38	13.43	-9.38	19.67	-18.76	13.45	-15.63	13.45	0.63	19.93

-13.13	13.85	-9.38	13.62	-8.75	19.93	-18.76	13.63	-15.01	13.62	0.00	20.20
-13.13	14.03	-8.75	13.78	-8.75	20.18	-18.76	13.80	-15.01	13.80	0.00	20.45
-12.51	14.20	-10.01	13.95	-8.75	20.43	-19.38	13.97	-15.01	13.97	0.63	20.70
-13.13	14.37	-9.38	14.13	-8.75	20.70	-18.76	14.15	-15.63	14.13	0.63	20.97
-13.13	14.53	-10.01	14.30	-8.75	20.95	-18.76	14.32	-15.01	14.32	-3.13	21.22
-13.13	14.72	-9.38	14.47	-8.75	21.20	-18.76	14.48	-15.63	14.48	-15.63	21.47
-13.13	14.88	-9.38	14.65	-3.13	21.45	-18.76	14.67	-15.01	14.65	-16.88	21.72
-13.13	15.05	-9.38	14.82	0.63	21.72	-18.76	14.83	-15.63	14.83	-15.63	21.98
-13.13	15.23	-10.01	14.98	0.63	21.97	-18.76	15.00	-15.01	15.00	-16.88	22.23
-13.13	15.40	-8.75	15.17	0.63	22.23	-18.76	15.17	-15.01	15.17	-16.88	22.48
-13.13	15.57	-8.75	15.33	0.63	22.48	-18.76	15.35	-15.63	15.35	-16.88	22.75
-13.13	15.75	-10.01	15.50	0.63	22.73	-18.76	15.52	-15.63	15.52	-15.63	23.00
-12.51	15.92	-8.75	15.68	0.63	23.00	-18.76	15.68	-15.63	15.68	-16.88	23.25
-12.51	16.08	-10.01	15.85	0.63	23.25	-18.76	15.87	-15.63	15.87	-15.63	23.52
-13.13	16.27	-9.38	16.03	-5.00	23.50	-18.76	16.03	-15.63	16.03	-15.63	23.77
-12.51	16.43	-9.38	16.20	-9.38	23.77	-19.38	16.20	-15.63	16.20	-17.51	24.03
-13.13	16.60	-8.75	16.37	-9.38	24.02	-19.38	16.38	-15.01	16.38	-16.88	24.28
-13.13	16.78	-10.01	16.55	-9.38	24.27	-18.76	16.55	-15.63	16.55	-16.88	24.53
-12.51	16.95	-10.01	16.72	-8.75	24.53	-18.76	16.73	-15.63	16.72	-16.88	24.80
-13.13	17.12	-9.38	16.88	-9.38	24.78	-18.76	16.90	-15.01	16.90	-16.88	25.05
-13.13	17.30	-10.01	17.07	-9.38	25.03	-19.38	17.07	-15.63	17.07	-16.88	25.30
-12.51	17.47	-8.75	17.23	-9.38	25.30	-18.76	17.25	-15.63	17.23	-16.88	25.55
-13.13	17.63	-9.38	17.40	-9.38	25.55	-19.38	17.42	-15.63	17.42	-16.88	25.82
-13.13	17.82	-9.38	17.57	-9.38	25.80	-18.76	17.58	-13.76	17.58	-17.51	26.07
-13.13	17.98	-10.01	17.75	-8.75	26.05	-19.38	17.75	-15.63	17.75	-17.51	26.33
-13.13	18.15	-10.01	17.92	-9.38	26.32	-18.76	17.93	-15.63	17.93	-16.88	26.58
-13.13	18.33	-10.01	18.08	-9.38	26.57	-18.76	18.10	-15.01	18.10	-17.51	26.83
-12.51	18.50	-9.38	18.27	-9.38	26.82	-19.38	18.28	-15.01	18.27	-17.51	27.08
-13.13	18.67	-10.01	18.43	-10.63	27.08	-18.76	18.45	-15.63	18.45	-18.13	27.35

-13.13	18.85	-9.38	18.60	-10.63	27.33	-18.76	18.62	-15.63	18.62	-18.13	27.60
-13.76	19.02	-10.01	18.77	-9.38	27.58	-19.38	18.80	-3.13	18.78	-18.13	27.85
-13.13	19.18	-9.38	18.95	-10.63	27.83	-19.38	18.97	0.63	18.97	-18.13	28.12
-13.13	19.35	-10.01	19.12	-9.38	28.10	-14.38	19.15	0.00	19.13	-16.88	28.37
-12.51	19.53	-10.01	19.28	-9.38	28.35	-1.88	19.32	0.63	19.30	-18.13	28.62
-13.13	19.70	-10.01	19.47	-10.63	28.62	0.00	19.48	0.63	19.47	-18.13	28.88
-13.13	19.88	-9.38	19.63	-10.63	28.87	0.63	19.67	-0.63	19.65	-17.51	29.13
-13.13	20.05	-9.38	19.80	-9.38	29.12	0.63	19.83	0.63	19.82	-1.25	29.40
-13.76	20.22	-9.38	19.98	-8.75	29.38	0.63	20.02	-0.63	19.98	0.63	29.65
-13.13	20.40	-10.01	20.15	-9.38	29.63	0.63	20.18	0.00	20.15	0.00	29.90
-13.13	20.57	-9.38	20.32	-10.63	29.88	0.00	20.35	0.00	20.33	0.00	30.17
-13.13	20.73	-9.38	20.48	-9.38	30.13	0.63	20.53	0.00	20.50	0.63	30.42
-3.75	20.92	-9.38	20.67	-9.38	30.40	0.63	20.70	0.00	20.67	0.63	30.67
-1.25	21.08	-10.01	20.83	-9.38	30.65	0.63	20.87	0.00	20.85	0.63	30.93
0.00	21.25	-9.38	21.02	-9.38	30.90	0.00	21.05	0.63	21.02	0.00	31.18
0.00	21.43	-2.50	21.20	-10.63	31.17	0.00	21.22	0.63	21.18	0.00	31.43
0.00	21.60	0.00	21.37	-9.38	31.42	0.63	21.38	-13.13	21.35	0.63	31.70
0.00	21.77	0.00	21.55	-9.38	31.67	-10.00	21.57	-15.01	21.53	0.00	31.95
0.00	21.95	-0.63	21.72	0.00	31.93	-18.76	21.73	-15.63	21.70	-15.63	32.20
-0.63	22.12	0.00	21.90	0.63	32.18	-18.76	21.92	-15.01	21.88	-16.88	32.47
0.00	22.30	0.00	22.07	0.63	32.43	-19.38	22.08	-15.01	22.05	-16.88	32.72
0.00	22.47	0.00	22.25	0.00	32.70	-19.38	22.25	-15.01	22.22	-16.88	32.97
-0.63	22.63	0.00	22.42	0.63	32.95	-19.38	22.43	-15.63	22.40	-17.51	33.23
0.00	22.82	0.63	22.60	0.63	33.20	-18.76	22.60	-15.01	22.57	-16.88	33.48
-0.63	22.98	0.63	22.77	0.63	33.47	-18.76	22.77	-15.01	22.73	-17.51	33.75
0.00	23.15	0.00	22.95	0.00	33.72	-18.76	22.95	-15.01	22.92	-18.13	34.00
-11.88	23.33	0.00	23.12	-7.50	33.98	-18.76	23.12	-15.01	23.08	-16.88	34.25
-13.13	23.50	0.00	23.28	-9.38	34.23	-18.76	23.28	-15.01	23.25	-18.13	34.52
-13.76	23.67	-3.75	23.47	-9.38	34.48	-19.38	23.47	-15.63	23.43	-18.13	34.77

-13.13	23.85	-9.38	23.63	-9.38	34.75	-18.76	23.63	-15.01	23.60	-17.51	35.03
-13.76	24.02	-9.38	23.80	-9.38	35.00	-18.76	23.80	-15.63	23.77	-17.51	35.28
-13.13	24.18	-10.01	23.98	-9.38	35.25	-18.76	23.97	-15.63	23.95	-18.13	35.53
-13.13	24.37	-8.75	24.15	-8.75	35.52	-19.38	24.15	-15.63	24.12	-17.51	35.80
-13.76	24.53	-9.38	24.32	-9.38	35.77	-18.76	24.32	-15.63	24.28	-18.13	36.05
-13.13	24.70	-9.38	24.50	-9.38	36.02	-19.38	24.48	-15.01	24.47	-17.51	36.30
-13.76	24.88	-9.38	24.67	-9.38	36.28	-18.76	24.65	-15.63	24.63	-17.51	36.55
-13.76	25.05	-9.38	24.83	-9.38	36.53	-19.38	24.83	-15.63	24.80	-18.13	36.82
-13.13	25.23	-9.38	25.00	-9.38	36.78	-18.76	25.00	-15.01	24.98	-17.51	37.07
-13.13	25.40	-9.38	25.18	-9.38	37.05	-18.76	25.17	-15.63	25.15	-18.13	37.33
-13.13	25.57	-9.38	25.35	-9.38	37.30	-18.76	25.35	-15.63	25.32	-18.13	37.58
-13.13	25.75	-9.38	25.53	-10.63	37.60	-19.38	25.52	-15.63	25.50	-18.13	37.85
-13.13	25.92	-9.38	25.70	-10.63	37.87	-19.38	25.68	-15.01	25.67	-17.51	38.10
-13.76	26.10	-10.01	25.88	-9.38	38.13	-19.38	25.87	-15.63	25.83	-18.13	38.35
-13.13	26.27	-10.01	26.05	-10.63	38.38	-18.76	26.03	-15.63	26.02	-18.13	38.62
-13.13	26.43	-9.38	26.22	-9.38	38.63	-18.76	26.20	-15.01	26.18	-18.76	38.87
-12.51	26.62	-10.01	26.40	-8.75	38.88	-19.38	26.38	-15.01	26.35	-18.13	39.12
-13.13	26.78	-9.38	26.57	-9.38	39.15	-18.76	26.55	-15.01	26.52	-18.13	39.38
-13.13	26.95	-9.38	26.73	-9.38	39.40	-19.38	26.72	-15.63	26.70	-18.76	39.63
-13.13	27.12	-10.01	26.92	-9.38	39.65	-18.76	26.90	-16.26	26.87	-18.13	39.88
-13.13	27.30	-10.01	27.08	-9.38	39.92	-19.38	27.07	-16.26	27.03	-18.13	40.15
-13.13	27.47	-10.01	27.25	-10.63	40.17	-19.38	27.25	-15.01	27.20	-1.25	40.40
-13.13	27.65	-9.38	27.43	-10.63	40.42	-18.76	27.42	-16.26	27.38	0.00	40.65
-13.13	27.82	-10.01	27.60	-10.63	40.67	-19.38	27.58	-16.26	27.55	0.00	40.90
-13.13	27.98	-9.38	27.77	-9.38	40.93	-19.38	27.75	-16.26	27.72	0.63	41.17
-13.13	28.15	-9.38	27.93	-10.63	41.18	-19.38	27.93	-15.63	27.88	0.00	41.42
-13.13	28.33	-9.38	28.12	-9.38	41.45	-19.38	28.10	-16.26	28.07	0.63	41.67
-13.13	28.52	-9.38	28.28	-9.38	41.70	-19.38	28.28	-15.63	28.23	0.63	41.93
-13.13	28.68	-10.01	28.47	-9.38	41.95	-18.76	28.45	-15.63	28.42	0.63	42.18

-13.13	28.87	-9.38	28.63	-3.13	42.22	-18.76	28.62	-15.63	28.58	-3.13	42.43
-13.13	29.03	-9.38	28.80	0.00	42.47	-19.38	28.80	-16.26	28.75		
-13.13	29.20	-9.38	28.98			-18.76	28.97	-15.63	28.92		
-13.13	29.38	-10.01	29.17			-18.76	29.13	-15.63	29.10		
-13.13	29.55	-10.01	29.33			-18.76	29.32	-7.50	29.27		
-13.13	29.73	-10.01	29.50			-18.76	29.48	0.00	29.43		
-13.13	29.90	-9.38	29.67			-18.76	29.65	-0.63	29.60		
-13.13	30.08	-10.01	29.85			-3.75	29.83	0.00	29.78		
-13.13	30.25	-9.38	30.02			0.63	30.00	0.63	29.95		
-13.76	30.42	-10.01	30.18			0.63	30.17	0.00	30.12		
-13.13	30.60	-10.01	30.37			0.00	30.35	-0.63	30.30		
-13.13	30.77	-10.01	30.53			0.00	30.52	0.00	30.47		
-13.13	30.93	-8.75	30.70			0.63	30.68	0.00	30.63		
-12.51	31.12	-9.38	30.87			0.00	30.87	0.63	30.82		
-13.13	31.28	-10.01	31.05			0.00	31.03	0.00	30.98		
-6.88	31.47	-10.01	31.22			0.00	31.22	0.00	31.15		
-0.63	31.63	-10.01	31.38			0.63	31.38	0.00	31.33		
-0.63	31.80	-10.01	31.58			0.63	31.55	-13.76	31.50		
0.00	31.97	-1.25	31.75			0.63	31.73	-15.01	31.67		
-0.63	32.15	0.00	31.93			0.00	31.90	-15.63	31.85		
0.00	32.32	-0.63	32.10			-13.13	32.07	-15.01	32.02		
0.00	32.48	0.63	32.28			-18.76	32.25	-15.63	32.20		
-0.63	32.67	0.00	32.45			-18.76	32.42	-15.63	32.37		
-0.63	32.83	-0.63	32.62			-19.38	32.58	-15.63	32.53		
0.00	33.00	-0.63	32.80			-19.38	32.77	-16.26	32.72		
0.00	33.18	0.63	32.97			-18.76	32.93	-16.26	32.88		
-0.63	33.35	0.00	33.13			-19.38	33.12	-16.26	33.05		
0.00	33.53	0.00	33.32			-19.38	33.28	-15.63	33.23		
-6.88	33.70	0.00	33.48			-19.38	33.45	-16.26	33.40		

-12.51	33.87	0.00	33.67	-19.38	33.63	-16.26	33.58
-13.13	34.03	0.63	33.83	-19.38	33.80	-16.26	33.75
-13.13	34.22	-8.75	34.00	-18.76	33.97	-15.63	33.92
-13.13	34.38	-9.38	34.18	-19.38	34.13	-15.63	34.08
-13.13	34.55	-9.38	34.35	-19.38	34.32	-15.63	34.27
-13.13	34.73	-9.38	34.52	-19.38	34.48	-15.63	34.43
-13.13	34.90	-9.38	34.70	-19.38	34.65	-15.63	34.60
-13.13	35.08	-10.01	34.87	-19.38	34.83	-15.63	34.77
-13.13	35.25	-10.01	35.03	-19.38	35.00	-15.63	34.95
-13.13	35.43	-10.01	35.22	-19.38	35.17	-15.63	35.12
-13.13	35.60	-9.38	35.38	-19.38	35.35	-16.26	35.28
-13.13	35.78	-9.38	35.55	-19.38	35.52	-16.26	35.47
-12.51	35.95	-10.01	35.73	-19.38	35.68	-16.26	35.63
-13.13	36.12	-9.38	35.90	-18.76	35.87	-15.63	35.80
-13.13	36.30	-10.01	36.07	-18.76	36.03	-16.26	35.97
-13.13	36.47	-10.01	36.23	-19.38	36.20	-15.63	36.15
-13.13	36.63	-9.38	36.42	-18.76	36.37	-16.26	36.32
-13.13	36.82	-9.38	36.58	-19.38	36.55	-16.26	36.50
-13.13	36.98	-9.38	36.77	-18.76	36.72	-16.26	36.67
-13.76	37.17	-8.75	36.93	-19.38	36.88	-15.63	36.83
-13.13	37.33	-10.01	37.10	-19.38	37.05	-16.26	37.02
-13.13	37.50	-9.38	37.27	-19.38	37.23	-16.26	37.18
-13.76	37.67	-10.01	37.45	-19.38	37.40	-16.26	37.35
-13.13	37.85	-10.01	37.62	-19.38	37.58	-16.26	37.53
-13.13	38.02	-9.38	37.78	-19.38	37.75	-16.88	37.70
-13.13	38.18	-9.38	37.97	-18.76	37.92	-16.26	37.87
-13.13	38.37	-10.01	38.13	-19.38	38.10	-16.26	38.05
-13.76	38.53	-9.38	38.30	-19.38	38.27	-16.26	38.22
-13.13	38.70	-9.38	38.48	-19.38	38.43	-16.88	38.38

-13.13	38.88	-10.01	38.65
-13.76	39.05	-9.38	38.82
-13.13	39.22	-9.38	39.00
-13.13	39.40	-10.01	39.17
-13.13	39.57	-10.01	39.33
-13.13	39.75	-10.01	39.52
-13.13	39.92	-9.38	39.68
-13.13	40.08	-8.75	39.85
-13.13	40.27	-10.01	40.03
-13.13	40.43	-10.01	40.20
-13.76	40.60	-10.01	40.37
-13.76	40.77	-10.01	40.55
-13.13	40.95	-10.01	40.72
-13.76	41.12	-10.01	40.88
-13.13	41.28	-10.01	41.07
-13.76	41.47	-9.38	41.23
-13.13	41.63	-9.38	41.40
-13.13	41.80	-10.01	41.57
-0.63	41.97	-10.01	41.75
-0.63	42.15	-9.38	41.92
0.00	42.32	0.00	42.10

-19.38	38.62	-16.26	38.57
-19.38	38.78	-16.26	38.73
-19.38	38.95	-16.26	38.92
-19.38	39.13	-16.88	39.08
-18.76	39.30	-16.26	39.25
-19.38	39.47	-16.26	39.43
-19.38	39.65	-15.63	39.62
-19.38	39.82	-1.25	39.78
-19.38	39.98	-0.63	39.97
-19.38	40.17	0.00	40.13
-6.25	40.33	0.00	40.30
0.00	40.50		

Table S6. Experimental data for Figure 5.
